# IL13Rα2 as a crucial receptor for Chi3l1 in osteoclast differentiation and bone resorption through the MAPK/AKT pathway

**DOI:** 10.1186/s12964-023-01423-7

**Published:** 2024-01-30

**Authors:** Weifeng Xu, Rui Chao, Xinru Xie, Yi Mao, Xinwei Chen, Xuzhuo Chen, Shanyong Zhang

**Affiliations:** Department of Oral Surgery, Shanghai Ninth People’s Hospital, Shanghai Jiao Tong University School of Medicine; College of Stomatology, Shanghai Jiao Tong University; National Center for Stomatology; National Clinical Research Center for Oral Diseases; Shanghai Key Laboratory of Stomatology, Shanghai, People’s Republic of China

**Keywords:** Chi3l1, IL13Rα2, Osteoclast differentiation, MAPK, AKT

## Abstract

**Background:**

Previous research has revealed that the 18 glycoside hydrolase gene family (GH18) member Chitinase 3-like 1 (Chi3l1) can regulate osteoclast differentiation and bone resorption. However, its downstream receptors and molecular mechanisms during osteoclastogenesis have yet to be elucidated.

**Methods:**

Initially, we conducted a comprehensive investigation to evaluate the effects of recombinant Chi3l1 protein or Chi3l1 siRNA on osteoclast differentiation and the RANKL-induced MAPK/AKT signaling pathways. Moreover, we used immunofluorescence and immunoprecipitation assays to identify IL13Rα2 as the downstream receptor of Chi3l1. Subsequently, we investigated the impact of IL13Rα2 recombinant protein or IL13Rα2-siRNA on osteoclast differentiation and the associated signaling pathways. Finally, we performed in vivo experiments to examine the effect of recombinant IL13Rα2 protein in an LPS-induced mouse model of cranial osteolysis.

**Results:**

Our findings highlight that the administration of recombinant Chi3l1 protein increased the formation of osteoclasts and bolstered the expression of several osteoclast-specific genes (TRAP, NFATC1, CTR, CTSK, V-ATPase d2, and Dc-STAMP). Additionally, Chi3l1 significantly promoted the RANKL-induced MAPK (ERK/P38/JNK) and AKT pathway activation, whereas Chi3l1 silencing inhibited this process. Next, using immunofluorescence and co-immunoprecipitation assays, we identified IL13Rα2 as the binding partner of Chi3l1 during osteoclastogenesis. IL13Rα2 recombinant protein or IL13Rα2-siRNA also inhibited osteoclast differentiation, and IL13Rα2-siRNA attenuated the RANKL-induced activation of the MAPK (ERK/P38/JNK) and AKT pathways, similar to the effects observed upon silencing of Chi3l1. Moreover, the promoting effect of recombinant Chi3l1 protein on osteoclastogenesis and the activation of the MAPK and AKT pathways was reversed by IL13Rα2 siRNA. Finally, recombinant LI13Rα2 protein significantly attenuated the LPS-induced cranial osteolysis and the number of osteoclasts in vivo.

**Conclusions:**

Our findings suggested that IL13Rα2 served as a crucial receptor for Chi3l1, enhancing RANKL-induced MAPK and AKT activation to promote osteoclast differentiation. These findings provide valuable insights into the molecular mechanisms of Chi3l1 in osteoclastogenesis, with potential therapeutic implications for osteoclast-related diseases.

Video Abstract

**Supplementary Information:**

The online version contains supplementary material available at 10.1186/s12964-023-01423-7.

## Background

Multinucleated giant cells navigate osteoclast differentiation and maturation through macrophage colony-stimulating factor (M-CSF)/colony-stimulating factor 1 receptor (CSF-1R) and receptor activator of nuclear factor kappa B ligand (RANKL)/receptor activator of nuclear factor kappa-B (RANK) [1, 2]. For example, the M-CSF/CSF-1R pathway can facilitate osteoclast precursor growth and mature osteoclast survival. Meanwhile, the RANKL/RANK pathway acts as an essential choreographer to gracefully orchestrate the dance of osteoclast differentiation and maturation [3]. RANKL, primarily emanating from osteoblasts and osteocytes, interacts with its partner RANK on osteoclast progenitors and mature osteoclasts. This interaction forms forming a synergistic complex. As osteoclast differentiation begins, the RANKL-RANK duet triggers several signal transduction pathways. These include the mitogen-activated protein kinase (MAPK), phosphoinositide-3-kinase/protein kinase B (PI3K/AKT), and nuclear factor kappa-B (NF-ΚB), which amplify the activity of many pivotal factors like nuclear factor of activated T-cells cytoplasmic 1 (NFATC1) and its downstream genes, including V-ATPase d2 (Atp6v0d2), cathepsin K (CTSK), calcitonin receptor (CTR), and tartrate-resistant acid phosphatase (TRAP) [4–7]. However, the overzealous indulgence of the osteoclasts (escalating numbers or increased activity) can induce the progression of many osteoclast-related diseases like osteoarthritis, periodontitis, and rheumatoid arthritis.

The glycoside hydrolase gene family 18 (GH18) includes chitinase 3-like 1 (Chi3l1) and chitinases and their cousins the chitinase-like proteins (CLPs). Chi3l1 is also known by several other names, including 38-kDa heparin-binding glycoprotein (gp38k) [8], Chondrex [9], human cartilage glycoprotein-39 (HC gp-39) [10], and YKL40 (based on its molecular weight (~ 40 kDa) and the first 3 N terminal amino acids -tyrosine(Y), lysine(K), and leucine(L)) [11]. The Chi3l1 gene on chromosome 1 (1q32.1) comprises 10 exons, generating a 383-amino acid protein [12]. It has been reported that Chi3l1 can bind to chitin, but not catalyze it, mainly because glutamic and aspartic acids in its chitinase active site are replaced by alanine and leucine, resulting in the loss of ability to catalyze chitin [13]. Furthermore, Chi3l1 can also bind heparin [12]. Chi3l1 has been extensively studied in various tumors (breast, colon, and prostate cancer) and inflammatory diseases. It has been observed that Chi3l1 is strongly associated with greater tumor aggressiveness, worse outcomes, and shorter survival rates. Additionally, Chi3l1 expression has also been demonstrated to correlate strongly with the severity of various inflammatory diseases, including asthma, rheumatoid arthritis, and arthrosis, among others [14]. Recent studies have suggested that Chi3l1 may be potentially involved in bone resorption. Mylin and colleagues have found that myeloma patients with increased Chi3l1 levels showed enhanced bone resorption activity, leading to faster and more severe bone destruction [15]. Chen’s work supports this, showing that inhibition of Chi3l1 expression attenuated bone destruction in a staphylococcus aureus-induced osteomyelitis mice model [16]. Di Rosa and colleagues contributed further by reporting that the Chi3l1 expression was significantly raised in the early osteoclast differentiation but inhibited in the later stage. They also found that silencing Chi3l1 with siRNA significantly impaired the bone resorption capacity of osteoclasts [17]. These studies indicated that Chi3l1 potentially assumes a vital function in osteoclast vitality. However, the specific receptor of Chi3l1 on osteoclast progenitor cell surfaces and the downstream molecular mechanisms underpinning this process still demand extensive elucidation.

IL-13 receptor α-chain 2 (IL13Rα2) was first isolated from the Caki-1 human renal cell carcinoma cell line in 1996 by Caput et al. [18]. Along with IL-13 receptor α-chain 1 (IL13Rα1), IL13Rα2 is one of the two receptors for IL13. Early studies have demonstrated that IL13Rα2 has a high affinity for IL13 and can competitively inhibit the IL13Rα1/IL4α receptor complex from binding to IL13, ultimately suppressing the function of IL13 [19]. Therefore, this receptor was considered a “decoy” for IL13. However, Fichtner-Feigl [20] in 2006 found that IL-13 promoted the expression of transforming growth factor β1 (TGF-β1) in macrophages via the IL-13Rα2 receptor, suggesting IL13Rα2 is not merely a “decoy” receptor. Subsequently, He and colleagues in 2013 identified IL13Rα2 as the first receptor for Chi3l1 in the monocyte-macrophage cell line THP-1. Through IL13Rα2, Chi3l1 activates the MAPK (ERK) and AKT signaling pathways to regulate a variety of cellular processes, including apoptosis, pyroptosis, inflammasome activation, oxidative damage, melanoma metastasis, and TGF-β1 production [21]. Xu and colleagues [22] also found that Chi3l1 promoted macrophage M2 polarisation through the IL13Rα2 receptor. Given that osteoclasts are derived from the monocyte-macrophage lineage, it has been suggested that Chi3l1 might influence osteoclast differentiation through IL13Rα2.

In our current study, we found a marked increase in the binding of Chi3l1 to IL13Rα2 during the initial phase of osteoclast differentiation of bone marrow-derived macrophages (BMMs). Furthermore, we also discerned that Chi3l1 significantly activated RANKL-induced MAPK (ERK/P38/JNK) and AKT signaling pathways through the IL13Rα2 receptor, facilitating the formation of osteoclasts and the expression of osteoclast biomarkers in vitro. Lastly, we demonstrated that recombinant IL13Rα2 (rIL13Rα2) protein significantly attenuated mouse cranial osteolysis induced by lipopolysaccharides (LPS).

## Materials and methods

### Reagents

Recombinant mouse proteins (Chi3l1, rIL13Rα2, RANKL, and M-CSF) were provided by R&D systems (USA). The Gibco BRL (USA) provided minimal essential medium alpha (α-MEM), fetal bovine serum (FBS), 0.25% EDTA-trypsin, and penicillin-streptomycin. RiboFECTTM transfection reagent, Chi3l1-siRNA, and IL13Rα2-siRNA came from RiboBio company (China). Primary antibodies targeting p-Akt (Ser473), Akt, p-ERK, ERK, p-JNK, JNK, p-P38, P38, p-P65, P65, and GAPDH were obtained from Cell Signaling Technology (CST, USA). The Abcam (USA) provided the primary antibody recognizing Chi3l1, which was used for immunofluorescence (IF) and western blot (WB). The primary antibody that recognized Chi3l1 used for co-immunoprecipitation (IP) and IL13Rα2 for IF and WB came from R&D systems (USA). Santa Cruz (USA) offered the primary antibody that recognized IL13Rα2 for IP.

### Mouse bone marrow macrophages (BMMs) preparation

As previously detailed, BMMs, the osteoclast precursor cells, were harvested and cultivated according to established protocols [5]. The delicate femurs and tibias of C57/BL6 male mice aged 4–6 weeks provided primary BMMs, and subsequently nurtured them in the α-MEM medium with 10% fetal bovine serum (FBS), 30 ng/mL M-CSF, and 100 U/mL penicillin/streptomycin in 5% CO2 at 37 °C. Following the three-day cultivation, half of the culture medium was replenished with fresh medium to maintain cell viability. On day 5, nonadherent cells and medium were gently taken out, and the remaining adherent cells were digested with 0.25% trypsin-EDTA.

### CCK-8 assay

To assess the effect of Chi3l1/rIL13Rα2, Chi3l1-siRNA/IL13Rα2-siRNA on the proliferative activity of BMMs. The 10^4^ BMMs per well were seeded into 96-well plates and cultivated in the α-MEM complete medium with 30 ng/ml M-CSF for 48 and 96 h. In this experiment, the concentrations of Chi3l1/rIL13Rα2 were 0, 125, 250, 500, 1000 ng/ml, and Chi3l1/IL13Rα2 siRNA or corresponding control were transfected into BMMs with riboFECT transfection buffer. According to the CCK-8 kit’s instructions, each well was gently rinsed thrice using 1× phosphate buffer solution (PBS) and then incubated with 110 μl of detection solution for 2 hours. The data were analyzed by measuring absorbance at 450 nm.

### Osteoclastogenesis and TRAP staining

To elucidate the impact of Chi3l1/rIL13Rα2, Chi3l1-siRNA/IL13Rα2-siRNA on osteoclastogenesis. The 1 × 10^4^ BMMs per well were seeded into 96-well plates and induced by the α-MEM complete medium with 30 ng/mL M-CSF and 100 ng/mL RANKL with the addition of Chi3l1/rIL13Rα2 or Chi3l1/IL13Rα2 siRNA transfection. The culture medium was replaced every 2 days, and mature multinuclear osteoclasts were observed on the fifth day. The osteoclasts that tested positive for TRAP and had at least three nuclei were captured by light microscopy and analyzed using the Image J software (NIH, Bethesda, MD).

### Elisa

Under the manufacturer’s esteemed instruction, the culture supernatant concentration of Chi3l1 in BMMs undergoing osteoclast differentiation was clarified with a mouse Chi3l1 ELISA kit (R&D, USA). The 50 μl assay dilution and 50 μl of culture supernatant or standard protein were added into each well (coated with Chi3l1 antibody) and cultivated for 2 h under room temperature conditions. Post incubation, each well was washed using wash buffer for 5 cycles, and 100 μl of horseradish peroxidase-labeled polyclonal Chi3l1 antibody detection solution was introduced and cultivated for another 2 hours at the same condition. Subsequently, after aspiration and yet another round of five meticulous washes, cells were inoculated with 100 μL substrate solution for 30 minutes without the influence of light. Lastly, 100 μl of the stop solution was applied to terminate the experiment. The Chi3l1 concentration was measured at the absorbance of 450 nm and calculated using ELISAcalc software.

### Quantitative PCR analysis

To assess the expression of Chi3l1, IL13Rα2, and osteoclastic-specific gene markers during osteoclastogenesis, quantitative polymerase chain reaction (PCR) was employed. RNA was isolated by utilizing the TRIzol reagent (Invitrogen). After determining the concentration of total RNA by NanoDrop 2000/2000c spectrophotometer at 260 nm and 280 nm, complementary DNA (cDNA) was reversely transcribed from total RNA according to the PrimeScript RT Reagent Kit’s instruction (TaKaRa, Japan). Finally, the quantitative real-time PCR was done through a StepOnePlus system (SYBR Premix Ex Taq, TaKaRa). Primers were as below: gapdh Forward:5′-GGTGAAGGTCGGTGTGAACG-3′Reverse:5′-CTCGCTC CTGGAAGATGGTG-3′ Chi3l1 Forward:5′-CGAGATT GCCTCCAAC ACT-3′ Reverse:5′-CATAAGAACGCAGGAACGG-3′ IL13Rα2 Forward:5′-GAAATGGAGCACACCTGGAGGAC-3′ Reverse:5′-GTGGCAG ACTCCCAGGAAATATCG-3′ TRAP Forward:5′-CTGGAGTGCACGATGCC AGCGACA-3′ Reverse:5′- TCCGTGCTCGGCGATG GACCAGA-3′ NFATC1 Forward:5′-TGCTCCTCCTCCTGCTGCTC-3′ Reverse:5′-GCAGAAGGTGGAGG TGCAGC-3’CTR Forward:5′-TGCAGA CAACTCTTGGTTGG-3′ Reverse:5′-TCGGTTTCTTCTCCTCTGGA-3′ CTSK Forward:5′-GGGAGAAAAACCTGA AGC-3′ Reverse:5′-ATTCTGGGGACTCAGA GC-3′ V-ATPase d2 Forward:5′-AAGCCTTTGTTTGACGCTGT-3′ Reverse: 5′-TTCGATGCCTCTGTGAGATG-3′ DC-STAMP Forward:5′-AAAACCCTTGGGCTGTTCTT-3′ Reverse:5′-AATCAT GGACGACTCCTTGG-3′. The gene expression levels were identified using the comparative threshold cycle (2^-ΔΔCt^) method [23].

### Western blot

To examine the Chi3l1 and its receptor IL13Rα2 expression levels, during osteoclast differentiation, there were 2 × 10^5^ BMMs per well seeded into 6-well plates and mixed with 30 ng/mL M-CSF and 100 ng/mL RANKL for 0, 1, 3, and 5 days. Next, the impact of Chi3l1 and IL13Rα2 on classical RANKL-induced pathways (MAPK, PI3K/AKT, NF-κB) in BMMs was analyzed. There were 2 × 10^5^ BMMs per well seeded into 6-well plates and cultured with 30 ng/mL M-CSF overnight. Then the cells were induced with 100 ng/mL RANKL for 0, 10, 20, 30, and 60 mins in the presence of elevated levels of Chi3l1 by adding recombinant protein or decreased expression of Chi3l1/IL13Rα2 by siRNA transfection. In addition, BMMs were also treated with recombinant chi3l1 protein for 0, 10, 20, 30, and 60 mins without RANKL. Following washing three times in PBS, the cells were processed with sodium dodecyl sulfate (SDS) lysis buffer containing protease and phosphatase inhibitors for 30 minutes on ice. The protein was quantified with bicinchoninic acid assay (BCA) (Beyotime Biotechnology, China), subsequently, the protein of each group was dissolved with 1x SDS-sample loading buffer and heated for 10 minutes. The gel electrophoresis was applied to separate 20 μg of total protein on 4–12% SurePAGE™ gels (GenScript, China), and then the separated proteins were transferred into 0.22 μm polyvinylidene difluoride (PVDF) membranes (Millipore, Germany). Next, they were blocked with 5% (w/v) skim milk for 1 hour. Following washing three times in PBS, the membranes were inoculated with primary antibodies (GAPDH, Chi3l1, IL13Rα2, p-AKT, AKT, p-ERK1/2, ERK, p-JNK, JNK, p-P38, P38, p-P65, P65) for overnight under 4 °C. Then the membranes were rinsed using 1x Tris-buffered saline with Tween (TBST) and exposed to the appropriate fluorescent secondary antibodies at the same temperature for 1 hour. The objective bands were obtained using Odyssey V3.0 image scanning (Li-COR. Inc., Lincoln, NE).

### Immunofluorescence

To determine the expression and localization of Chi3l1 and IL13Rα2 during the osteoclast differentiation of BMMs, we performed immunofluorescence analysis. 5 × 10^4^ BMMs per well were carefully seeded on confocal dishes and cultured overnight. Cells were then stimulated to undergo osteoclast formation with the addition of 30 ng/mL M-CSF and 100 ng/mL RANKL for 0, 1, 3, and 5 days. These cells were fixed with 4% paraformaldehyde at room temperature for 15 minutes. Then, they were washed with 1x PBS to remove the non-adherent cells. Cells were permeabilized through 0.5% Triton X-100 for 5 minutes and blocked with the immunofluorescence-blocking solution for 1 hour. Next, each sample was mixed with the Chi3l1 primary antibody (dilution ratio 1:100) and allowed to incubate overnight at 4 °C. After a PBS rinse, the corresponding fluorescent secondary antibody was applied and incubated at room temperature for 1 h. This sequence was reiterated with the IL13Rα2 primary antibody (dilution ratio 1:500) and fluorescent secondary antibody (dilution ratio 1:500). Finally, a DAPI solution (5 μg/mL) was served to illuminate the cell nuclei. All images were obtained from a fluorescence microscope.

### Co-immunoprecipitation (IP)

To determine whether Chi3l1 binds to the IL13Rα2 receptor during osteoclast differentiation, a co-immunoprecipitation (co-IP) assay was performed. There were 5 × 10^6^ BMMs seeded in a 10 cm dish for 1 day and incubated for 24 h with or without 100 ng/mL RANKL. The co-IP of Chi3l1 to IL13Rα2 or IL13Rα2 to Chi3l1 was then carried out following the instructions of the Immunoprecipitation Kit (Beyotime Biotechnology, China). After rinsing in PBS, the BMMs were lysed with the provided IP lysis buffer. Protein concentrations were accurately determined using BCA assay, with all samples normalized to a concentration of 1 μg/μl. Magnetic beads (20 μl) were sequentially washed three times with 500 μl 1x TBST to ensure purity. Antibodies against Chi3l1 (sheep polyclonal antibody, AF2649, R&D) and IL13Rα2 (mouse monoclonal antibody, sc-134,363, Santa Cruz) were diluted to the manufacturer’s recommended concentrations in TBS. At the same time, species-matched IgG controls were prepared at comparable concentrations. Protein A + G magnetic beads were conjugated to the primary antibodies or control IgG at room temperature with continuous rotation for 1 hour, followed by three washes in 1 x TBST to remove non-specifically bound antibodies, then the antibody-conjugated beads or control IgG-conjugated beads were added into 500 μl of protein sample and incubated overnight at 4 °C on a rotary shaker. After this incubation, the bead-protein complexes were washed three times with 1x TBST and resuspended in 100 μl of 1 x SDS-PAGE Loading Buffer. The samples were then denatured at 95 °C for 5 minutes and subjected to magnetic separation to isolate the target proteins. The expression levels of IL13Rα2 and Chi3l1 were sequentially assessed by Western blot analysis as described above.

### LPS-induced calvarial osteolysis mice model

Experiments on the animal were approved by the Animal Ethics Committee from the Ninth People’s Hospital of Shanghai Jiao Tong University School of Medicine. The experiment was conducted under the principles for the Ethical Conduct in the Care and Use of Nonhuman Animals in Research by the American Psychological Association. To investigate the function of rIL13Rα2 in LPS-induced mouse calvarial osteolysis, we established a mouse model using a previously described protocol [24]. Briefly, twenty 6–8 week-old male C57BL/6 mice, obtained from Shanghai Biocay Biotechnology Co., Ltd., were housed in a sterile animal room of SPF grade at the Shanghai Laboratory Animal Center. The mice were randomly divided into four groups: control, LPS (10 mg/kg body weight), 0.5μg/ml- rIL13Rα2 + LPS, and 1 μg/ml- rIL13Rα2 + LPS. The specific procedures were as follows: After soaking with LPS and/or rIL13Rα2 (100 μl), a gelatin sponge (4 mm × 4 mm × 2 mm) was introduced into the sagittal midline of the skull with general anesthesia. Subcutaneous injections were then given every 2 days. On day 14, the calvarial bones were harvested and fixed in 4% paraformaldehyde for the next experiments.

### Micro-computed tomography (CT) scanning

To scrutinize the extent of calvarial osteolysis in each group, high-resolution micro-CT scanning was performed (μCT-100, SCANCO Medical AG, Switzerland; scan resolution-10 μm, scan energy-70 kV/200 μA, 300 ms). The region of interest (ROI) referred to a square surrounding the area of resorption. The Bone Volume/Tissue Volume (BV/TV) ratio was automatically calculated. Pore counts and percent porosity were measured for each following previously established methods [25].

### Histological staining

To histologically evaluate the degree of calvarial osteolysis, the 10% EDTA solution was applied to decalcify specimens at room temperature for 2 weeks. After decalcification, specimens were rinsed with running water overnight, followed by dehydration, transparent procession, wax immersion, paraffin embedding, and section. Then hematoxylin and eosin (HE) and TRAP staining were conducted, and high-quality images were obtained from a Digital Scanner (Aperio CS20, Leica, Germany). TRAP-positive cells were quantified using the ImageJ software (NIH).

### Statistics

The obtained values were presented as mean ± standard deviation (SD). Data were processed with SPSS 13.0 (Chicago). Following a test for homogeneity of variance, the Student’s t-test was conducted to clarify the statistical significance between the experimental and control groups. The one-way analysis of variance (ANOVA) was employed for comparisons among multiple groups. A *P*-value below 0.05 was deemed a statistical difference.

## Results

### Chi3l1 recombinant protein promotes but chi31l-siRNA inhibits osteoclast differentiation of BMMs

**Fig. 1:**
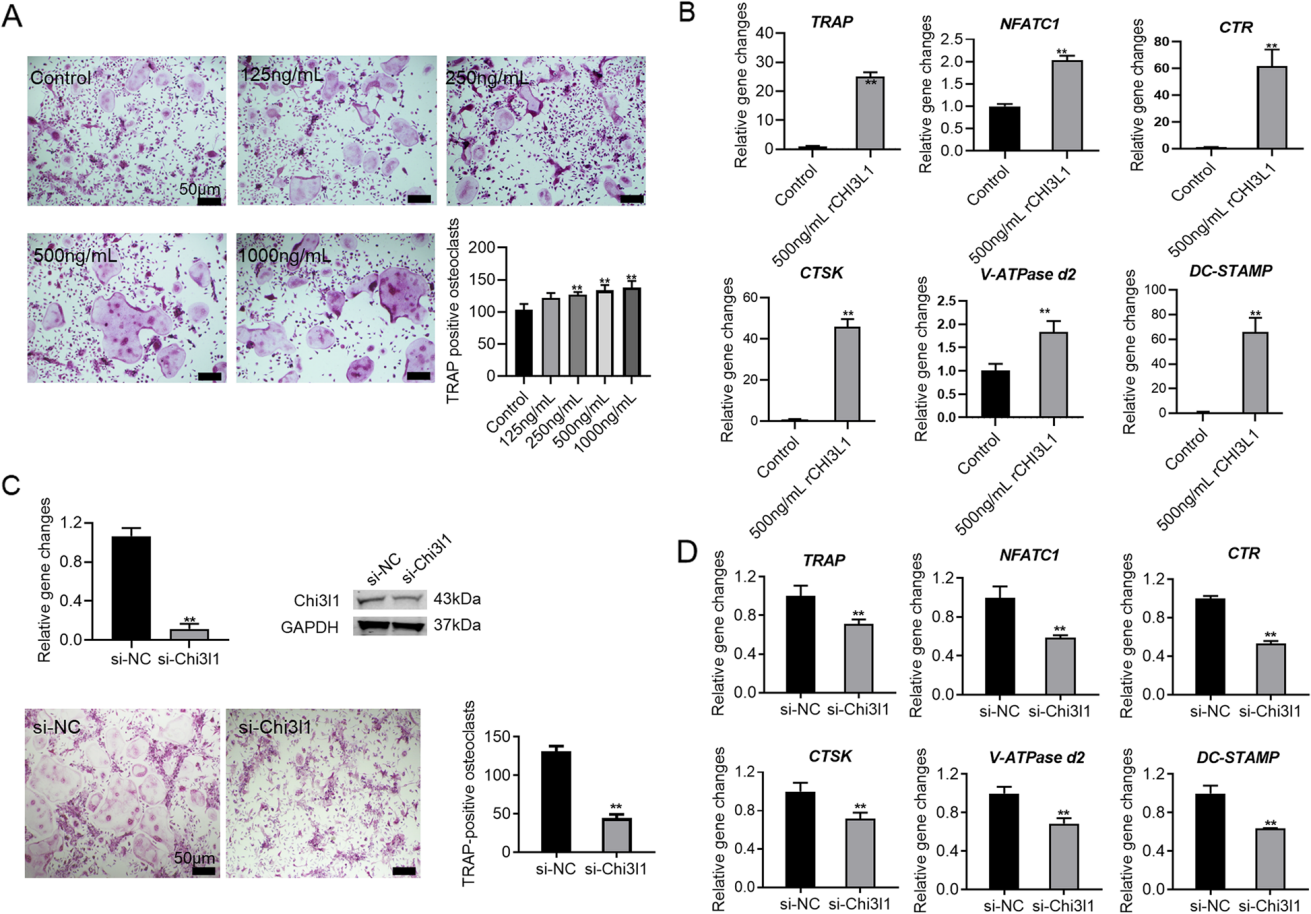
Chi3l1 recombinant protein promotes but Chi3l1-siRNA inhibits osteoclast differentiation of BMMs. **A** M-CSF, RANKL, and various concentrations of Chi3l1 recombinant protein (0, 125, 250, 500, 1000 ng/ml) were applied to treat BMMs for 5 days. TRAP staining showed that recombinant Chi3l1 protein dose-dependently induced mature multinucleated osteoclast formation. **B** PCR analysis showed that the mRNA expression level of osteoclast differentiation-related genes was significantly upregulated by 500 ng/ml Chi3l1 recombinant protein. **C** After silencing Chi3l1 with Chi3l1 siRNA, M-CSF and RANKL were applied to treat BMMs for 5 days to induce osteoclast differentiation. TRAP staining showed that mature multinucleated osteoclasts were significantly inhibited by Chi3l1 siRNA. **D** PCR analysis also revealed that the mRNA expression of the osteoclast differentiation-related genes was significantly down-regulated by Chi3l1 siRNA. **P < 0.01. Data are presented as the means ± SD

To investigate the impact of Chi3l1 on osteoclast differentiation in BMMs, we induced osteoclast formation in BMMs with M-CSF and RANKL and exposed the cells to varying concentrations of recombinant Chi3l1 protein (0, 125, 250, 500, 1000 ng/ml). The control group exhibited multinucleated giant osteoclasts after induction with M-CSF and RANKL for 5 days. Nevertheless, the recombinant Chi3l1 protein prompted larger and numerous mature multinucleated osteoclasts compared to the control, and it exerted a dose-dependent promoting effect. Moreover, the promoting effect of 500 ng/ml Chi3l1 recombinant protein was approximately the same as that of 1000 ng/ml (Fig. 1A). The results of PCR experiments also confirmed that Chi3l1 recombinant protein (500 ng/ml) significantly enhanced the expression of *TRAP*, *NFATC1*, *CTR*, *CTSK*, *V-ATPase d2*, and *Dc-STAMP.* which was consistent with the TRAP staining finding (Fig. 1B). On the contrary, when the expression of Chi3l1 was silenced with Chi3l1-siRNA, as confirmed by PCR and Western blot analysis (Fig. 1C), there was a marked decrease in the number and size of generated osteoclasts, and the expression of osteoclast-specific marker genes was significantly inhibited (Fig. 1C-D). Additionally, the CCK-8 experiment revealed that neither treatment with recombinant Chi3l1 protein nor downregulation of Chi3l1 expression using Chi3l1 siRNA had any effect on the proliferative ability of BMMs (Supplemental Fig. S1A-B).

### Chi31l regulates RANKL-induced activation of the MAPK/AKT signaling pathway

**Fig. 2:**
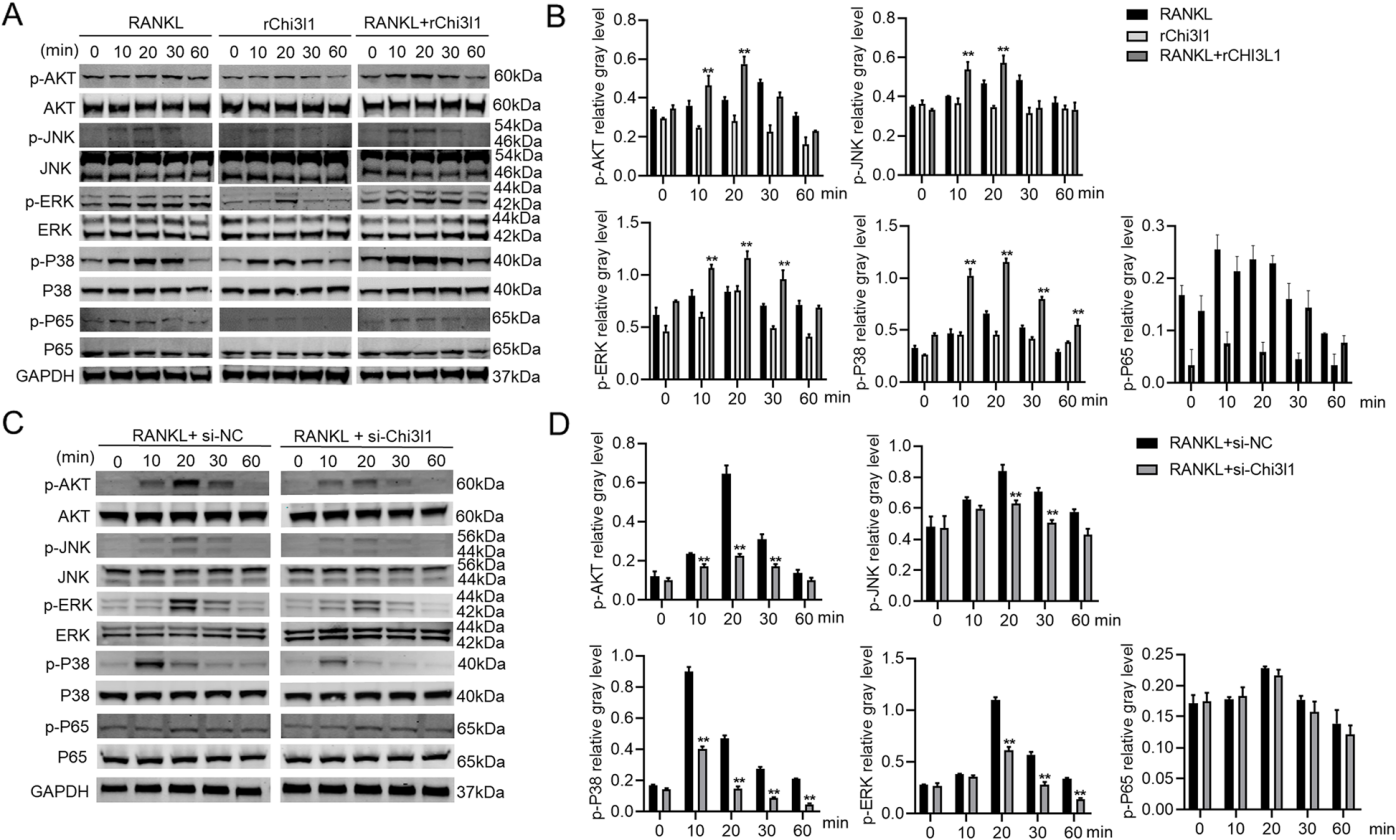
Chi3l1 regulates the RANKL-induced MAPK/AKT pathway activation. **A** BMMs were induced with RANKL, 500 ng/ml Chi3l1 recombinant protein, or RANKL+ 500 ng/ml Chi3l1 recombinant protein for 0, 10, 20, 30, and 60 mins. Then the signaling pathway phosphorylation was analyzed by western blot. **B** Quantitative densitometric analysis was performed to normalize p-AKT, p-ERK, p-P38, p-JNK, and p-P65 expressions from (**A**). **C** After silencing Chi3l1 expression in BMMs using Chi3l1 siRNA for 48 h, RANKL was used for stimulating BMMs for 0, 10, 20, 30, and 60 mins. The signaling pathway activation was analyzed by western blot. **D** Quantitative densitometric analysis was conducted to normalize the aforementioned expressions from (**C**). **P < 0.01. Data are presented as the means ± SD

The result revealed that Chi3l1 recombinant protein dose-dependently promoted osteoclast differentiation of BMMs, but Chi3l1 silencing inhibited this process. To clarify the function of Chi3l1 on classical signaling pathways (MAPK/PI3K-AKT/NF-κB) during osteoclast differentiation. First, we investigated the impact of 500 ng/mL Chi3l1 recombinant protein on RANKL-induced activation of MAPK (ERK/P38/JNK), PI3K/AKT (AKT), and NF-κB (P65) via Western blot analysis. As illustrated in Fig. 2A and B, the results showed that RANKL significantly increased the phosphorylation levels of MAPKs (p-ERK, p-P38, p-JNK), PI3K/AKT (p-AKT), and NF-κB (p-P65) in BMMs of the control group. Phosphorylation levels showed an initial increase followed by a decrease. When BMMs were stimulated with recombinant Chi3l1 protein alone, the phosphorylation levels of the above proteins were enhanced but at a slightly lower level than that of RANKL alone. However, the phosphorylation of p-P65 was basically not activated by Chi3l1 recombinant protein. Co-stimulation of RANKL and Chi3l1 recombinant protein significantly enhanced the phosphorylation levels that were notably higher than those of RANKL or Chi3l1 recombinant protein alone. However, this co-stimulation also had no function on p-P65. Subsequently, After downregulating the expression of Chi3l1 in BMMs by transfection with Chi3l1-siRNA before stimulation of RANKL, we found that Chi3l1 silencing significantly suppressed the phosphorylation levels of p-ERK, p-P38, p-JNK, and p-AKT compared to the control group. As expected, the p-P65 phosphorylation was also not affected by Chi3l1 inhibition (Fig. 2C-D). These findings indicate that the MAPK and AKT signaling pathways, rather than the NF-κB pathway, are responsible for Chi3l1 regulating the osteoclast differentiation of BMMs.

### Increased binding of Chi3l1 to IL13Rα2 during early osteoclastic differentiation

**Fig. 3:**
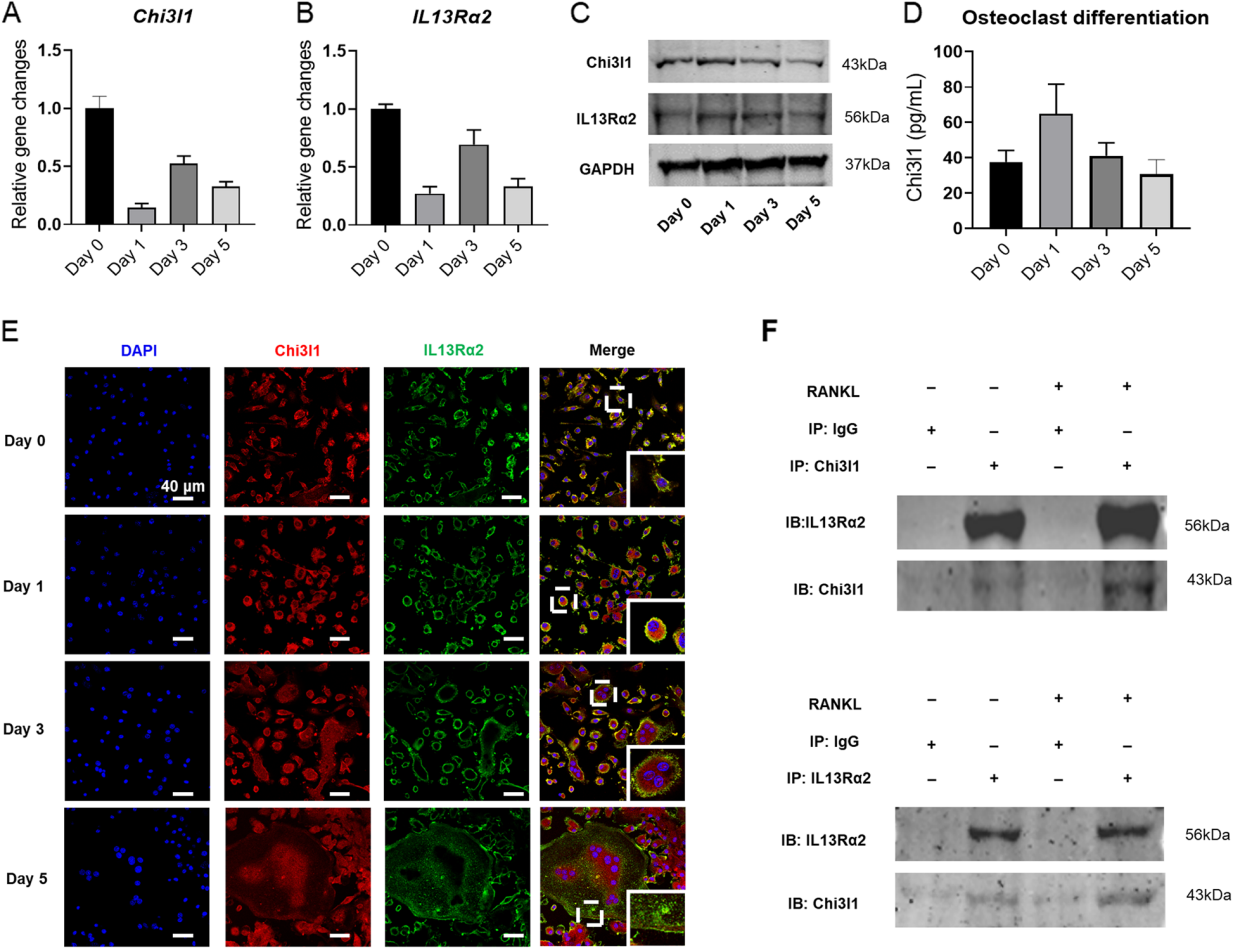
Increased binding of Chi3l1 to IL13Rα2 during early osteoclastic differentiation. M-CSF and RANKL were applied to induce osteoclastic differentiation of BMMs for 0, 1, 3, and 5 days. **A, B** The mRNA levels of Chi3l1 and IL13Rα2 during osteoclastogenesis. **C** The protein expressions of Chi3l1 and IL13Rα2 during osteoclastogenesis. **D** The culture supernatant concentration of Chi3l1 during osteoclastogenesis. **E** The location of Chi3l1 and IL13Rα2 in BMMs during osteoclast differentiation, as detected by immunofluorescence (IF) co-staining. **F** The binding of Chi3l1 and IL13Rα2 in BMMs, as confirmed by co-immunoprecipitation (IP), was increased after 24 hours of RANKL stimulation, w **P < 0.01. Results are presented as means ± SD

We next attempted to explore the possibility that IL13Rα2 may serve as a crucial receptor for Chi3l1 during the osteoclast differentiation of BMMs. First, we examined the expression of Chi3l1 and IL13Rα2 during the osteoclast differentiation process. PCR analysis revealed that both Chi3l1 and IL13Rα2 gene mRNA levels decreased on day 1, increased slightly on day 3, and then decreased again on day 5 (Fig. 3A-B). A similar pattern in protein expression of Chi3l1 and IL13Rα2 was also observed by western blot, with both proteins reaching their highest levels on day 1 before gradually declining (Fig. 3C). Consistent with the western blot results (Fig. 3D), ELISA assay also presented that the supernatant concentration of Chi3l1 increased at the beginning and then gradually decreased during the differentiation of osteoclasts, indicating that Chi3l1 and IL13Rα2 exhibited similar expression patterns during osteoclastic differentiation. Next, to examine whether Chi3l1 binds to IL13Rα2 during osteoclast differentiation, immunofluorescence co-staining and immunoprecipitation (co-IP) experiments were undertaken. Figure 3E showed that Chi3l1 was mainly detected on the cytoplasm and plasma membrane when BMMs were cultured with M-CSF, whereas IL13Rα2 was predominantly detected on the membrane, and the co-localization of both proteins was visible on the cell membrane. Subsequently, M-CSF and RANKL were applied to treat BMMs for 1, 3, and 5 days. As shown in Fig. 3E, the result demonstrated a significant increase in Chi3l1 and IL13Rα2 expression and their co-localization on the membrane on day 1, followed by a decrease on day 3. However, by day 5, the expression and co-localization of Chi3l1 and IL13Rα2 in mature multinucleated osteoclasts were almost undetectable. Finally, co-IP demonstrated that endogenous Chi3l1 and endogenous IL13Rα2 in BMMs could interact with each other, and this interaction was dramatically enhanced after 24 h stimulation of RANKL (Fig. 3F). Therefore, these above results suggest that IL13Rα2 may be a key receptor of Chi3l1 in the regulation of osteoclast differentiation of BMMs.

### IL13Rα2 silencing and rIL13Rα2 inhibit osteoclast differentiation

**Fig. 4:**
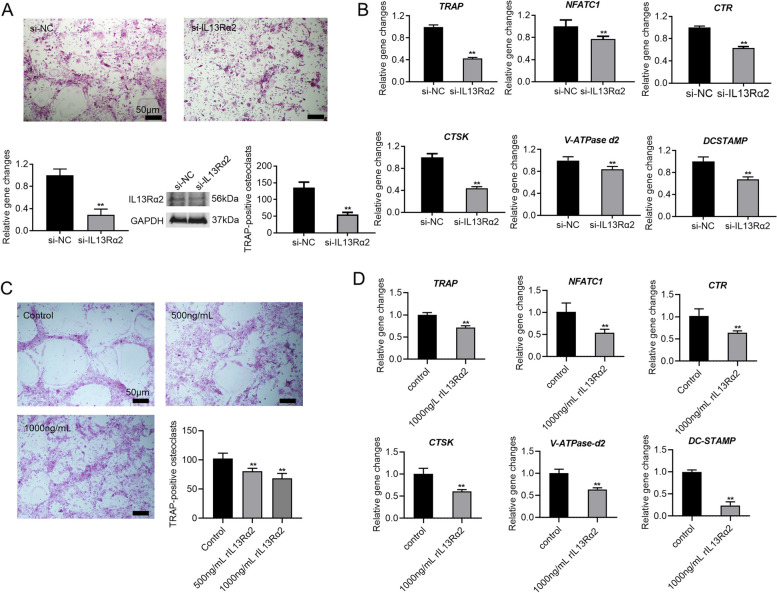
IL13Rα2 silencing and rIL13Rα2 protein inhibit osteoclast differentiation. **A** After silencing the IL13Rα2 expression in BMMs with IL13Rα2 siRNA, M-CSF and RANKL were applied to treat BMMs for 5 days to induce osteoclast differentiation. TRAP staining revealed that the formation of mature multinucleated osteoclasts was inhibited by IL13Rα2 siRNA. **B** PCR analysis also revealed that the mRNA expression of the osteoclast differentiation-related genes was significantly down-regulated by IL13Rα2 siRNA. **C** M-CSF and RANKL were applied to treat BMMs for 5 days to induce osteoclast differentiation with 500 ng/ml or 1000 ng/ml rIL13Rα2 protein. TRAP staining discovered that mature multinucleated osteoclasts were dose-dependently attenuated by rIL13Rα2 protein. **D** PCR results demonstrated that the mRNA expression of the osteoclast differentiation-related genes was dramatically suppressed by 1000 ng/ml rIL13Rα2 protein. **P < 0.01. Data are presented as the means ± SD

Given that IL13Rα2 has been identified as a receptor for Chi3l1 on the cell membrane of BMMs during early osteoclast differentiation. Next, we examined the impact of IL13Rα2 knockdown on osteoclast differentiation. After silencing IL13Rα2 expression using IL13Rα2 siRNA, as confirmed by PCR and western blot analysis, we treated BMMs with M-CSF and RANKL to induce osteoclast differentiation. The results revealed that silencing IL13Rα2 significantly inhibited the mature osteoclast formation and its related gene expression (Fig. 4A-B). Furthermore, to competitively inhibit the binding of Chi3l1 to IL13Rα2 on the cell membrane of BMMs, BMMs were supplemented with 500 ng/ml or 1000 ng/ml of rIL13Rα2 protein during osteoclast differentiation. We observed that the formation of mature osteoclasts was dose-dependently attenuated by rIL13Rα2 (Fig. 4C). PCR analysis also presented that rIL13Rα2 (1000 ng/mL) significantly suppressed the osteoclast-specific gene expressions (Fig. 4D). Furthermore, we also found that neither IL13Rα2 silencing nor treatment with rIL13Rα2 protein affected the proliferative ability of BMMs (Supplemental Fig. S1B-C). These data indicate that both IL13Rα2 silencing and rIL13Rα2 protein inhibit osteoclast differentiation.

### IL13Rα2 silencing attenuates the RANKL-induced MAPK/AKT signaling pathway activation

**Fig. 5:**
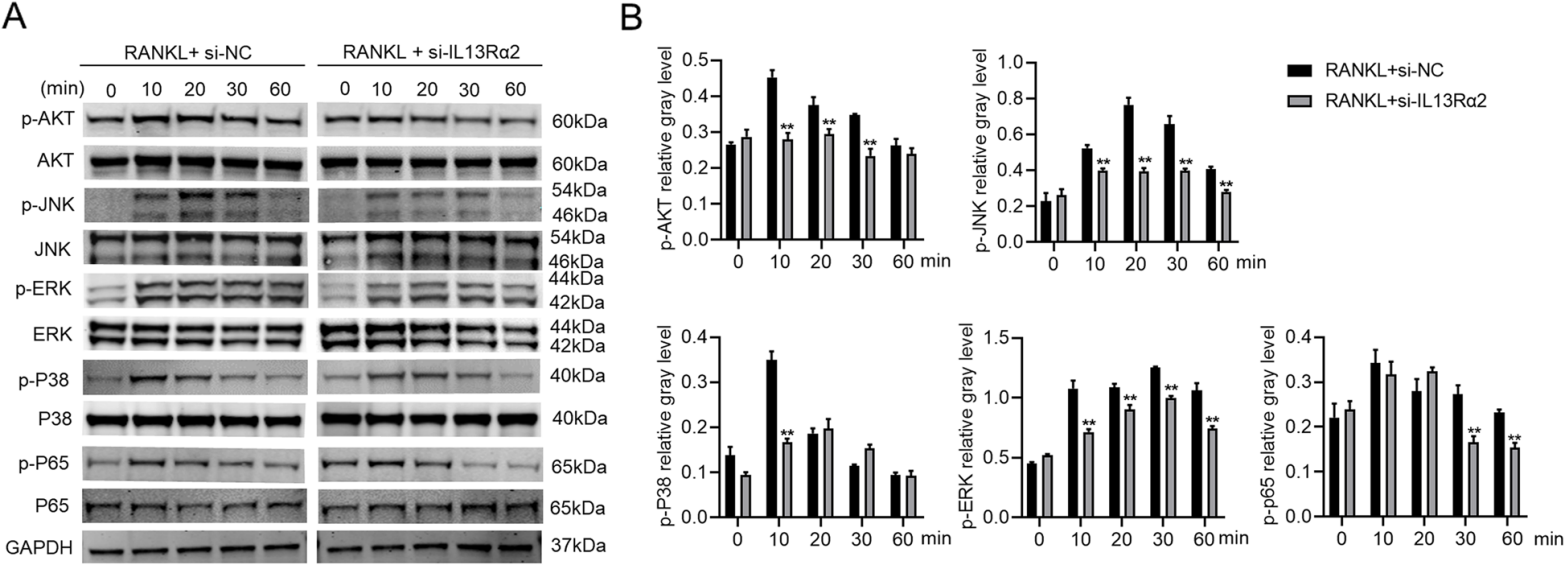
IL13Rα2 silencing suppresses the RANKL-induced activation of MAPK/AKT pathway. **A** After silencing IL13Rα2 expression in BMMs with IL13Rα2 siRNA for 48 h, BMMs were exposed to RANKL for 0, 10, 20, 30, and 60 mins. Then the phosphorylation of signaling pathways was analyzed by western blot. **B** Quantitative densitometric analysis was performed to normalize p-AKT, p-ERK, p-P38, p-JNK, and p-P65 expressions as shown in (**A**). **P < 0.01. Results are presented as means ± SD

Considering that Chi3l1 promotes the RANKL-induced MAPK and AKT pathway activation and interacts with IL13Rα2 during osteoclast differentiation of BMMs, we studied the impact of IL13Rα2 silencing on these pathways activated by RANKL in BMMs. After silencing IL13Rα2 expression with siRNA for 48 h, BMMs were exposed to RANKL for 0, 10, 20, 30, and 60 mins. The western blot results (Fig. 5A-B) indicated that IL13Rα2 knockdown reduced phosphorylation levels of RANKL-induced p-AKT, p-ERK, p-P38, and p-JNK. Additionally, IL13Rα2 knockdown had no effect on the phosphorylation of p-P65. These findings align with the results obtained from Chi3l1 silencing. These results suggest that IL13Rα2 might be a receptor for Chi3l1, promoting the activation of RANKL-induced MAPK and AKT pathway.

### IL13Rα2 silencing reverses the effects of Chi3l1 on osteoclast differentiation and the activation of RANKL-induced MAPK/AKT pathway

**Fig. 6:**
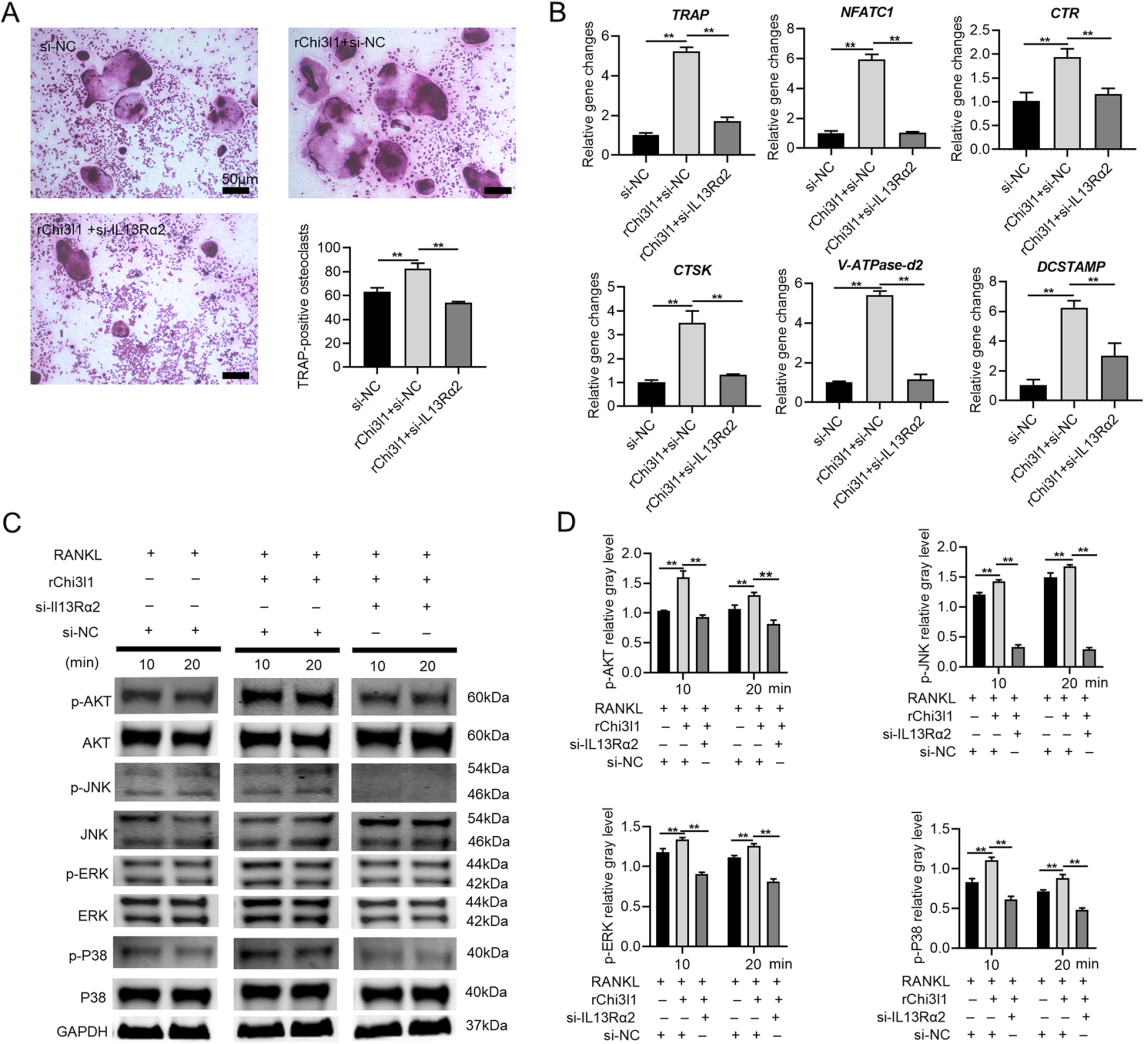
IL13Rα2 silencing reverses Chi3l1-promoting osteoclastogenesis and the activation of RANKL-induced MAPK/AKT pathway. **A** IL13Rα2 siRNA or NC-siRNA was transfected into BMMs, and after 48 hours, M-CSF and RANKL were used to treat BMMs with or without 500 ng/ml Chi3l1 recombinant protein. TRAP staining revealed that IL13Rα2 silencing inhibited the formation of mature multinucleated osteoclasts, which was increased by Chi3l1 recombinant protein and RANKL. **B** PCR analysis also showed that IL13Rα2 silencing downregulated the mRNA expression of the osteoclast differentiation-related genes that were promoted by Chi3l1 recombinant protein and RANKL. **C** After silencing IL13Rα2 expression in BMMs with siRNA for 48 h, BMMs were induced with RANKL for 10 and 20 minutes with or without 500 ng/ml Chi3l1 recombinant protein. The activation of MAPK (ERK/P38/JNK) and AKT signaling pathways was analyzed by western blot. **D** Quantitative densitometric analysis was performed to normalize p-AKT, p-ERK, p-P38, p-JNK, and p-P65 expressions as shown in (**C**). **P < 0.01. Results are presented as means ± SD

To clarify the role of IL13Rα2 as a receptor for Chi3l1 in facilitating osteoclast differentiation of BMMs. First, we downregulated IL13Rα2 expression in BMMs by transfection with IL13Rα2-siRNA. leading to downregulate the expression of IL13Rα2. After 48 hours, M-CSF, RANKL, and 500 ng/mL recombinant Chi3l1 protein were added to induce osteoclast differentiation for 5 days. As shown in Fig. 6A and B, Chi3l1 recombinant protein enhanced the formation of mature osteoclasts and the mRNA expressions of osteoclast-specific genes compared to the control group, but IL13Rα2 silencing reversed this process. Next, we investigated the effect of silencing IL13Rα2 on the MAPK and AKT pathways activated by co-stimulation of RANKL and Chi3l1 recombinant protein. Since the phosphorylation levels of p-AKT, p-ERK, p-P38, and p-JNK, activated by RANKL and recombinant Chi3l1 protein, exhibited significant upregulation at 10 and 20-minute time points. we transfected BMMs with IL13Rα2-siRNA for 48 hours to knock down IL13Rα2 expression before co-stimulating them with RANKL and 500 ng/mL Chi3l1 recombinant protein for 10 and 20 minutes. The phosphorylation levels of the aforementioned factors were apparently enhanced by co-stimulation of RANKL and Chi3l1 compared to the control group. However, IL13Rα2 silencing reversed these phosphorylation levels activated by co-stimulation with RANKL and Chi3l1(Fig. 6C-D). These results indicate that IL13Rα2 is a pivotal receptor of Chi3l1 for activating the MAPK and AKT signaling pathways, thereby facilitating the osteoclast differentiation of BMMs.

### rIL13Rα2 protein suppresses LPS-induced calvarial osteolysis

**Fig. 7:**
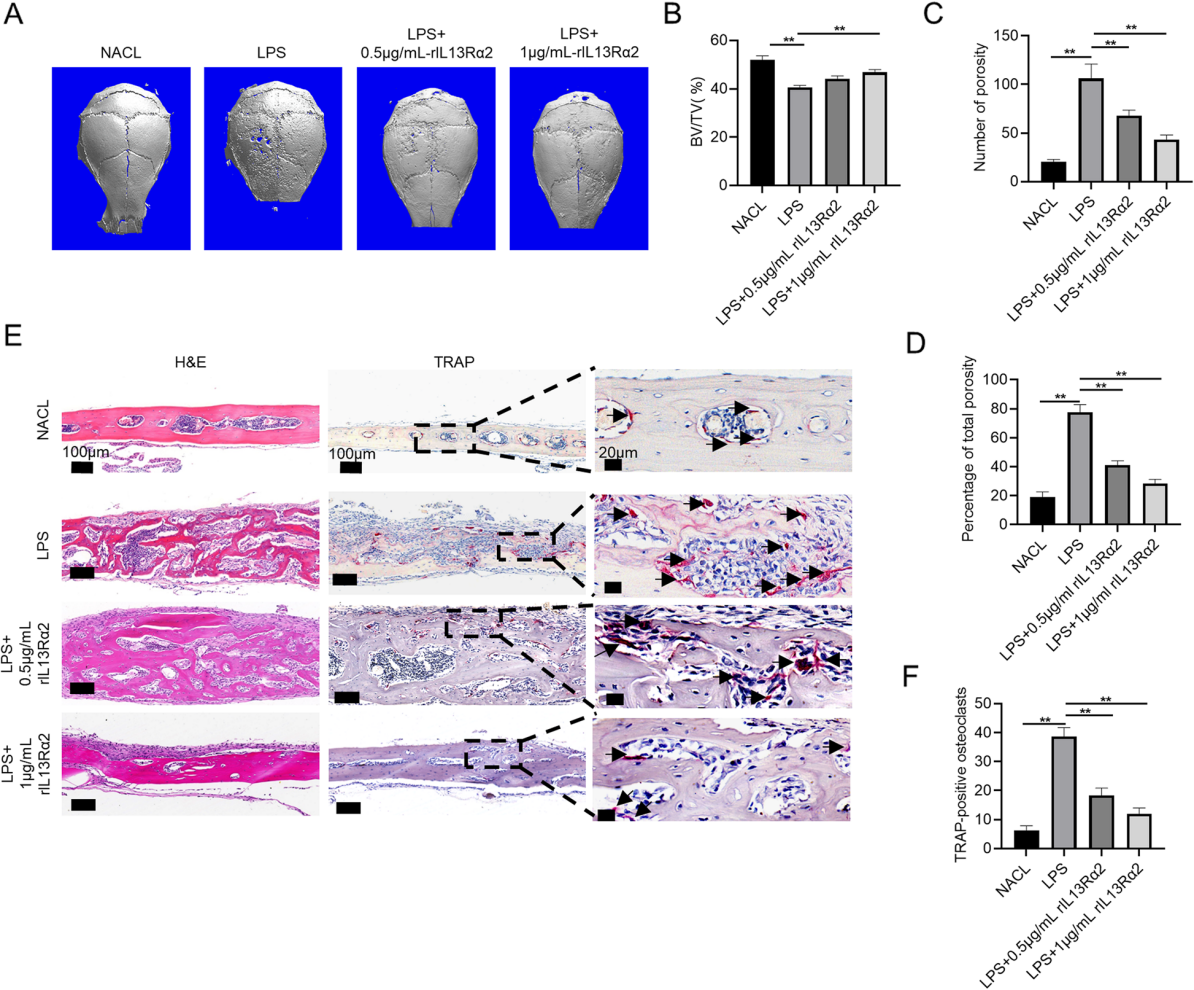
rIL13Rα2 protein suppresses LPS-induced calvarial osteolysis. A mouse model was constructed and divided into saline, LPS, LPS+ 0.5 μg/ml rIL13Rα2, and LPS + 1 μg/ml-rIL13Rα2 groups. **A** Representative images of three-dimensional reconstructions of the calvarial bone were obtained using a micro-CT scanner. **B** Histograms of the bone volume fraction (BV/TV). **C** Histograms of the pore numbers. **D** Histograms of the percentage of bone porosity. **E** Representative images of HE and TRAP staining. **F** Histograms of the number of TRAP-positive osteoclasts. **P < 0.01. Results are presented as means ± SD

Since it is obvious that rIL13Rα2 protein can competitively inhibit the binding of Chi3l1 to membrane IL13Rα2, which was important for osteoclast differentiation. We observed that the rIL13Rα2 protein inhibited the osteoclast formation and its related gene expressions. So rIL13Rα2 protein may represent a new treatment for osteoclast-related bone diseases. Therefore, we established an LPS-induced calvarial osteolysis mouse model to elucidate its therapeutic effects. As illustrated in Fig. 7A, three-dimensional micro-CT reconstruction revealed that the calvarial bone surface in the LPS group featured numerous large and deep bone resorption sites. On the contrary, treatment with 0.5 or 1 μg/mL of rIL13Rα2 protein significantly reduced the LPS-induced destruction of calvarial bone. The group that received the higher concentration of 1 μg/mL exhibited a more effective therapeutic response, resulting in a calvarial bone surface closely resembling that of the control group. As expected, micro-CT analysis (Fig. 7B-D) also demonstrated that the LPS group had significantly lower BV/TV and higher pore counts and bone porosity compared to the control group. Nevertheless, treatment with rIL13Rα2 protein resulted in a significant increase in BV/TV and a reduction in bone porosity percentage and pore counts compared to the LPS group.

Next, we conducted a histological analysis using HE and TRAP staining to evaluate the therapeutic effectiveness of rIL13Rα2 protein in LPS-induced bone osteolysis. Figu 7E and F illustrated that LPS resulted in considerably more severe destruction of the calvarial bone and induced a higher number of TRAP-positive osteoclasts compared to the control group. However, co-administration of rIL13Rα2 protein with LPS significantly decreased cranial bone damage and suppressed the TRAP-positive osteoclast vitality, and the high-dose group demonstrated greater therapeutic efficacy than the low-dose group. Collectively, the above findings indicate that rIL13Rα2 protein inhibits osteoclast activation and suppresses LPS-induced bone osteolysis.

## Discussion

Osteoclasts are a unique type of mononuclear macrophage cells, mainly derived from hematopoietic stem cell (HSC) progenitors. These mature osteoclasts, differentiated from these osteoclastic precursors, are driven by two classical stimulatory pathways (M-CSF/CSF-1R and RANKL/RANK) and participate in bone resorption, which is dynamically balanced with the osteogenic capacity of osteoblasts under physiological conditions [1, 2, 6]. Osteoclasts-related diseases like osteoarthritis and periodontitis arise when the number or the bone resorption capacity of osteoclasts significantly exceeds normal levels. This has led to the development of various osteoclast-targeting drugs, such as bisphosphonates, denosumab, and estrogens. These drugs have undoubtedly proved very successful in treating osteoclastic bone resorption diseases. However, clinical use of these drugs is limited by several adverse events, including jaw osteonecrosis, bone fractures, breast cancer, deep vein thrombosis, and even instances of stroke [26, 27]. This critical situation demands a prompt reaction in the form of novel medications and interventions, specifically tailored to minimize these unwanted side effects while retaining efficacy.

Previous studies have identified Chi3l1 as a biomarker in many malignancies and inflammatory diseases [14], but the role of Chi3l1 in bone resorption diseases was poorly understood. Research by Mylin and Chen reported that the Chi3l1 level may be associated with bone resorption [15, 16]. In 2014, Di Rosa directly demonstrated Chi3l1’s function in osteoclast differentiation and bone resorption through in vitro cellular assays, where Chi3l1 downregulation inhibited osteoclastic bone resorption [17]. But unfortunately, the molecular mechanisms and downstream receptors of Chi3l1 in osteoclast differentiation have not been reported.

This research initially explored the impact of Chi3l1 and its underlying molecular mechanism during osteoclast differentiation. First, M-CSF and RANKL alongside exogenous recombinant Chi3l1 protein were applied to treat BMMs, revealing that Chi3l1 concentration-dependently promoted osteoclast formation. Additionally, 500 ng/ml recombinant Chi3l1 protein significantly augmented the mRNA level of osteoclast-specific genes. Conversely, in agreement with Di Rosa’s findings that downregulation of Chi3l1 expression inhibited bone resorption capacity [17], silencing Chi3l1 in BMMs using siRNA inhibited the formation of mature osteoclasts and the mRNA expression of related genes. As we know, NFATC1 is a pivotal transcription factor and is regulated by the NF-κB pathway and the activator protein 1 (AP-1). Moreover, NFATC1 exhibits self-amplification via binding to its own promoter, leading to overexpression of downstream genes like CTSK and TRAP [3, 7]. Moreover, V-ATPase d2 and DC-STAMP are critical for the osteoclast precursor cells differentiation into multinucleated osteoclasts, while V-ATPase d2 is also involved in the bone resorption via modulating the extracellular acidification of osteoclasts [28, 29]. CTSK, a secretion from active osteoclasts, is implicated in the process of collagen breakdown and the decomposition of other matrix proteins throughout bone resorption activities [30]. The membrane-expressed CTR in osteoclasts also participates in bone resorption, with CTR knockout mice exhibiting a phenotype of dramatically increased bone mass [31]. TRAP is a biomarker for osteoclasts, and TRAP staining remains the most commonly used method for identifying osteoclasts. These findings suggest that Chi3l1 directly participates in osteoclastogenesis.

The MAPK, PI3K-AKT, and NF-κB pathways are three important pathways for osteoclastogenesis [6, 7]. Next, we attempted to assess the Chi3l1 function on these pathways in BMMs induced by RANKL, and we found that exogenous Chi3l1 recombinant protein promoted MAPK and AKT pathways in BMMs, albeit to a slightly lesser extent than RANKL. When BMMs were induced by RANKL and Chi3l1 together, the pathway activation was significantly enhanced compared to that of RANKL or Chi3l1 alone. Conversely, silencing Chi3l1 significantly inhibited the activation of RANKL-induced pathways. Unexpectedly, we found that Chi3l1 had little effect on the NF-κB pathway because the phosphorylation level of p-P65 was not altered by either Chi3l1 recombinant protein or Chi3l1 silencing. Collectively, these findings indicate that the MAPK and AKT pathways are responsible for Chi3l1 to enhance osteoclast differentiation of BMMs.

Given the significant effect of Chi3l1 on osteoclastogenesis, identifying its receptor was the next priority. Despite the fact that Chi3l1 was first isolated from bovine mammary secretions (nonlactating period) in 1988 by Rejman et al., it was not until 2013 that IL13Rα2 was identified as the first receptor for Chi3l1 [21, 32]. IL13Rα2 was first cloned and characterized from the Caki-1 cell line by Caput and colleagues [18]. IL13Rα2 is a 56 kDa transmembrane glycoprotein with 4 N-glycosylation sites in the extracellular domain [33]. Both IL13Rα1 and IL13Rα2 are receptors for IL13, yet IL13Rα2 is only 27% identical to the IL13Rα1. Unlike IL13Rα1, which requires the assistance of IL4Rα to bind IL13, IL13Rα2 binds directly to IL13 with high affinity in the absence of IL4α. The high affinity of IL13Rα2 for IL13 is sufficient to compete with the IL13Rα1/IL4Rα complex for binding to IL13, thereby inhibiting the downstream signaling pathway of IL13 [34]. Therefore, IL13Rα2 has traditionally been viewed as a “decoy” receptor for IL13, functioning to antagonize IL13 activity. However, in 2006, Fichtner-Feigl found that IL-13Rα2 mediates IL13-induced promotion of AP-1 expression and production of transforming growth factor β1 (TGF-β1) in macrophages. This was the first demonstration that IL-13Rα2 was not just a “decoy” receptor [20]. In 2013, IL13Rα2 was also identified as the first receptor for Chi3l1 in regulating oxidant injury, inflammasome activation, pyroptosis, apoptosis, and antibacterial responses [21]. The extracellular domain (ECD) of Chi3l1 containing chitin-binding motif (CBM) and the extracellular domain (ECD) of IL13Rα2 are required for the binding of Chi3l1 and IL13Rα2 independent of IL13. Besides, Chi3l1 increases the binding to IL13Rα2 by inhibiting the N-glycosylation of IL13Rα2 [21, 35]. Xu [22] also reported that Chi3l1 enhanced macrophage M2 polarization by activating MAPK and AKT signaling pathways via IL13Rα2 receptor. Since osteoclast is also derived from mononuclear macrophage lineage, it is reasonable to hypothesize that IL13Rα2 may be a key receptor for Chi3l1 during the osteoclast differentiation of BMMs.

Initially, we demonstrated that Chi3l1 and IL13Rα2 shared a similar expression pattern during osteoclastogenesis. However, slight differences were observed between the mRNA and protein expression levels of Chi3l1 and IL13Rα2. The Chi3l1 and IL13Rα2 mRNA levels were initially decreased, then slightly increased before decreasing during osteoclastogenesis, whereas their protein levels increased initially and then decreased. These discrepancies could be attributed to the extensive transcription and translation of pre-existing Chi3l1 and IL13Rα2 mRNA into proteins in BMMs cells after stimulation with RANKL and M-CSF. This subsequently facilitated the early binding of Chi3l1 to IL13Rα2, thereby promoting osteoclast differentiation. However, this early process might not involve the synthesis of new or enough Chi3l1 and IL13Rα2 mRNA. In addition, both protein and mRNA expression levels of Chi3l1 and IL13Rα2 decreased significantly during the late stages of osteoclast differentiation. Hoover and colleagues [36] also reported that undifferentiated Bone marrow-derived mesenchymal stem cells (BMSCs) had elevated Chi3l1 mRNA expression, but Chi3l1 proteins were undetectable. However, once BMSCs differentiated into osteoblasts or chondrocytes, there was rapid and extensive transcription and translation of Chi3l1 mRNA into protein. Furthermore, ELISA assay also showed that the change in the concentration of Chi3l1 in the medium supernatant during osteoclast differentiation was consistent with the Western blot finding. The pattern of Chi3l1 expression during osteoclastogenesis similarly mirrored the findings of Di Rosa et al. [17]. Next, Immunofluorescence co-staining was applied to detect the Chi3l1 and IL13Rα2 expression and localization during the osteoclast differentiation of BMMs, we found the co-localization of Chi3l1 and IL13Rα2 was mainly observed on the membrane of BMMs. The expression and co-localization of Chi3l1 and IL13Rα2 significantly increased on the membrane after RANKL stimulation for 24 h but markedly decreased in early fused osteoclasts on day 3, and with little expression and co-localization on the membrane of mature osteoclasts on day 5, which was also consistent with the results by western blot. Finally, co-immunoprecipitation confirmed that Chi3l1 and IL13Rα2 could bind to each other in BMMs, with this binding notably enhancing after 24 h of RANKL stimulation. Therefore, IL13Rα2 interacted with Chi3l1 during the early osteoclast differentiation, suggesting that it might serve as a crucial receptor for Chi3l1 in the regulation of osteoclast differentiation.

Considering that IL13Rα2 might be the receptor of Chi3l1, the study investigated the function of IL13Rα2 on osteoclast formation. Similar to the inhibitory effect of Chi3l1 silencing, IL13Rα2 silencing also inhibited osteoclast differentiation of BMMs. Furthermore, we also found that the effect of Chi3l1 recombinant protein in promoting osteoclast differentiation was reversed by IL13Rα2 silencing. Subsequently, we examined the role of IL13Rα2 silencing on the aforementioned signaling pathways, which were activated by RANKL during osteoclastogenesis. We found that IL13Rα2 silencing dramatically attenuated the phosphorylation of ERK, P38, JNK, and AKT but did not affect the p-P65, paralleling the results obtained from Chi3l1 silencing. Moreover, the MAPK and AKT pathways activation induced by co-stimulation with Chi3l1 and RANKL was reversed by IL13Rα2 silencing. These data suggest that Chi3l1 activates downstream MAPK and AKT signaling pathways via binding to the IL13Rα2 receptor on the membrane of BMMs, which is consistent with the findings reported by other researchers. For example, Chen [37] reported that Chi31l, secreted by tumor M2 macrophages, interacted with the IL13Rα2 receptor to stimulate the MAPK signaling pathway, subsequently facilitating tumor metastasis. Kawada [38] discovered that Chi3l1 promoted the production of IL-8 and monocyte chemoattractant protein-1 (MCP-1) via the MAPK pathway activation. Li [39] found that Chi3l1 inhibited miR-590-3p expression through the PI3K/Akt pathway, thereby promoting tumor angiogenesis. In Xu’s research [22], Chi3l1 was shown to promote M2 activation and VEGF secretion, thereby driving the activation of the MAPK signaling pathway via the IL13Rα2 receptor. Xue’s study [40] reported that Chi3l1 promoted the migration and the tube formation of human umbilical vein endothelial cells (HUVECs) by activating MAPK and AKT signaling pathways via the IL13Rα2 receptor in vitro. Collectively, these studies indicate that the regulation of cell behaviors by the Chi3l1/IL13Rα2 axis primarily occurs through the MAPK and AKT pathways, although the precise regulatory pathways may vary between cell types.

Previous studies have demonstrated that there are three distinct forms of IL13Rα2: membrane IL13Rα2 protein, intracellular IL13Rα2 protein, and soluble extracellular IL13Rα2 protein (sIL13Rα2). sIL13Rα2 has been considered to be a “decoy” receptor, competitively binding to the ligands of IL13Rα2 to inhibit downstream signaling pathways [41–43]. Therefore, we investigated the effect of recombinant IL13Rα2 (rIL13Rα2) protein on osteoclast differentiation. Our results demonstrated that rIL13Rα2 protein concentration-dependently suppressed osteoclast differentiation, suggesting that rIL13Rα2 protein could competitively inhibit the binding of Chi3l1 with membrane IL13Rα2, thereby impeding osteoclast differentiation. Following this, an LPS-induced calvarial osteolysis mouse model was established to scrutinize its function on osteoclast-related bone disorders. We found that rLI13Rα2 protein significantly suppressed the LPS-induced cranial osteolysis and the number of osteoclasts, with a better therapeutic effect in the high-concentration group. It is noteworthy that the origins of mouse and human sIL13Rα2 differ significantly. Mouse sIL13Rα2 could be generated either by the direct transcription and translation of a selective variant of IL13Rα2 mRNA (the mRNA lacking exon 10) or the degradation of matrix metalloproteinase 8 (MMP8), which degrades the membrane IL13Rα2 to form sIL13Rα2 [44]. In contrast, only one form of human IL13Rα2 mRNA exists, which translates into membrane-bound IL13Rα2 [44], suggesting that delivering exogenous sIL13Rα2 to targeted sites in humans has therapeutic potential for diseases related to osteoclasts.

Despite yielding some valuable insights into the mechanism of Chi3l1 regulating osteoclastogenesis, our study is not without limitations. First, a large number of receptors for Chi3l1 have now been identified, including IL13Rα2, transmembrane protein 219 (TMEM219), galectin-3, chemoattractant receptor-homologous molecule expressed on Th2 cells (CRTH2), CD44, heparin, hyaluronic acid, receptor for advanced glycation end products (RAGE), and syndecan-1/αVβ3 [14, 45]. For instance, TMEM219, binding to IL13Rα2, has been shown to mediate Chi3l1-induced activation of the ERK1/2 and AKT pathways [46]. Consequently, additional experiments are necessary to ascertain if other receptors or molecular mechanisms for Chi3l1 are implicated in osteoclast differentiation. Secondly, the mechanism of Chi3l1 expression during osteoclast differentiation also required further elucidation. Moreover, the therapeutic potential of sIL13Rα2 in osteoclasts-related diseases such as osteoarthritis and rheumatoid arthritis warrants further exploration.

## Conclusions

**Fig. 8:**
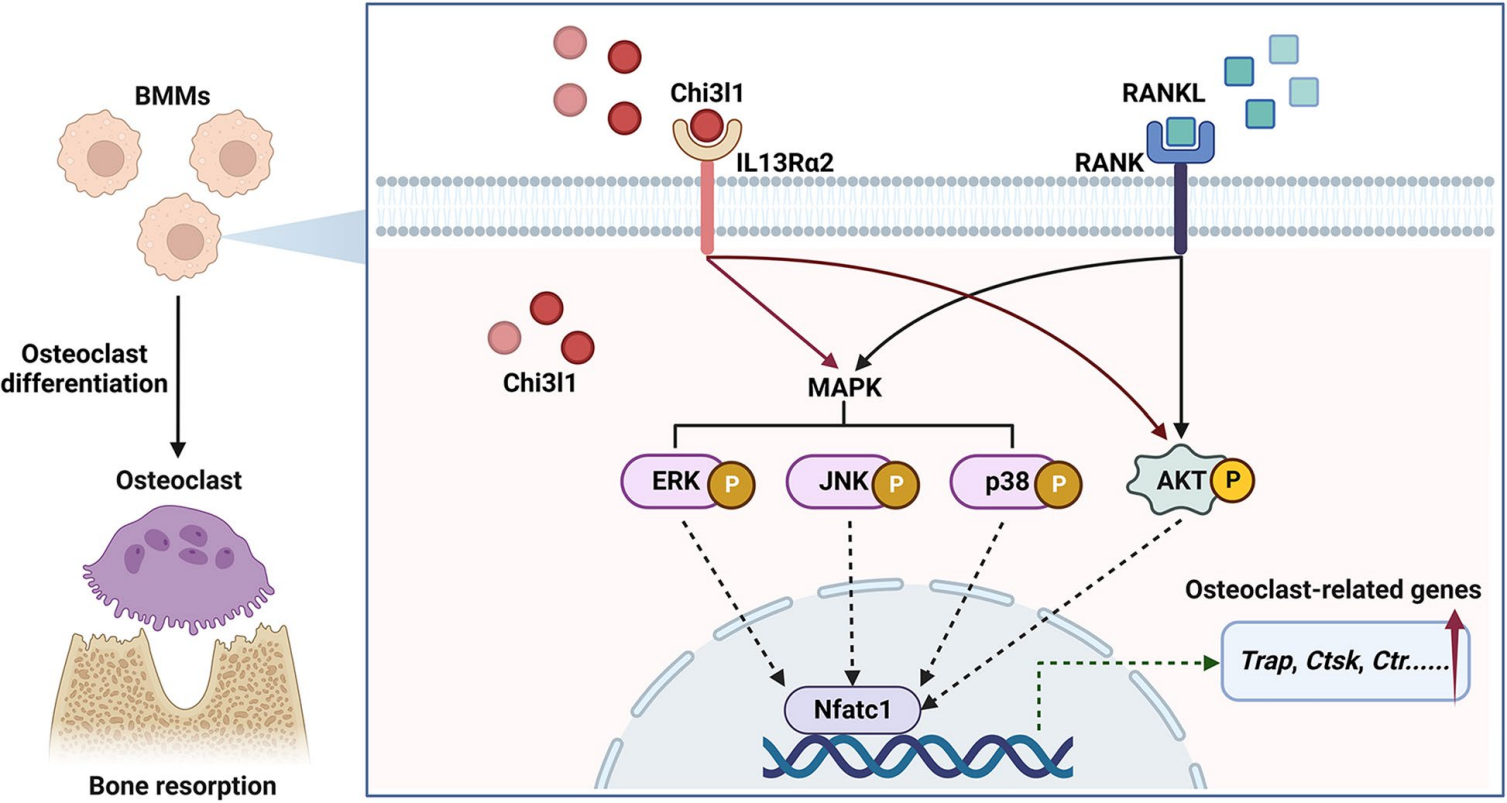
Receptor and Molecular Mechanisms of Chi3l1 in Osteoclastogenesis. Chi3l1 interacts with IL13Rα2 on the bone marrow-derived macrophages (BMMs) membrane, acting as osteoclast precursors. This interaction promotes the activation of the RANKL-induced MAPK and AKT signaling pathways, eventually leading to osteoclast differentiation

In conclusion, our findings reveal that IL13Rα2 served as a receptor for Chi3l1, stimulating the MAPK and AKT pathways and thereby facilitating the differentiation of BMMs into osteoclasts in vitro (Fig. 8). Furthermore, we observed that the rIL13Rα2 protein significantly suppressed the LPS-induced cranial osteolysis and osteoclast formation in vivo. By demonstrating the mutual relationship between Chi3l1 and IL13Rα2 in osteoclastogenesis for the first time, our study suggested that IL13Rα2 could represent a potential therapeutic target for the management of osteoclast-associated diseases.

### Supplementary Information

**Additional file 1: Supplemental Figure 1. **Neither recombinant Chi3l1/sIL13Rα2protein nor Chi3l1/sIL13Rα2 silencing had any effect on the proliferative ability of BMMs. A BMMs were treated with M-CSF and various concentrations of chi31l recombinant protein (0, 125, 250, 500, 1000ng/ml) for 48h and 96h, then the proliferative ability of BMMs was determined by the CCK-8 experiment. B After silencing Chi3l1or IL13Rα2 expression in BMMs with siRNA for 48h, BMMs were treated with M-CSF for 48h and 96h, then the proliferative ability of BMMs was determined by the CCK-8 experiment. C BMMs were treated with M-CSF and various concentrations of rIL13Rα2 protein (0, 125, 250, 500, 1000ng/ml) for 48h and 96h, then the proliferative ability of BMMs was determined by the CCK-8 experiment. **P < 0.01. Results are expressed as means ± SE.

## Data Availability

The raw data substantiating the conclusions drawn in this study can be available from the corresponding author upon a reasonable request.

## Abbreviations

GH18: 18 glycoside hydrolase gene family
Chi3l1: Chitinase 3-like 1
IL13Rα2: IL-13 receptor α-chain 2
BMMs: Mouse bone marrow macrophages
BMSCs: Bone marrow-derived mesenchymal stem cells
M-CSF: Macrophage colony-stimulating factor
CSF-1R: Colony-stimulating factor 1 receptor
RANKL: Receptor activator of nuclear factor kappa B ligand
RANK: Receptor activator of nuclear factor kappa-B
MAPK: Mitogen-activated protein kinase
PI3K/AKT: Phosphoinositide-3-kinase/protein kinase B
NF-κB: Nuclear factor kappa-B
NFATC1: Nuclear factor of activated T-cells cytoplasmic 1
Atp6v0d2: V-ATPase d2
CTSK: Cathepsin K
CTR: Calcitonin receptor
TRAP: Tartrate-resistant acid phosphatase
