# Supplementary material for: IL13Rα2 as a crucial receptor for Chi3l1 in osteoclast differentiation and bone resorption through the MAPK/AKT pathway
Source: Cell Commun Signal. 2024 Jan 30;22:81. doi: 10.1186/s12964-023-01423-7 (PMC10826115; doi:10.1186/s12964-023-01423-7)

Figure1

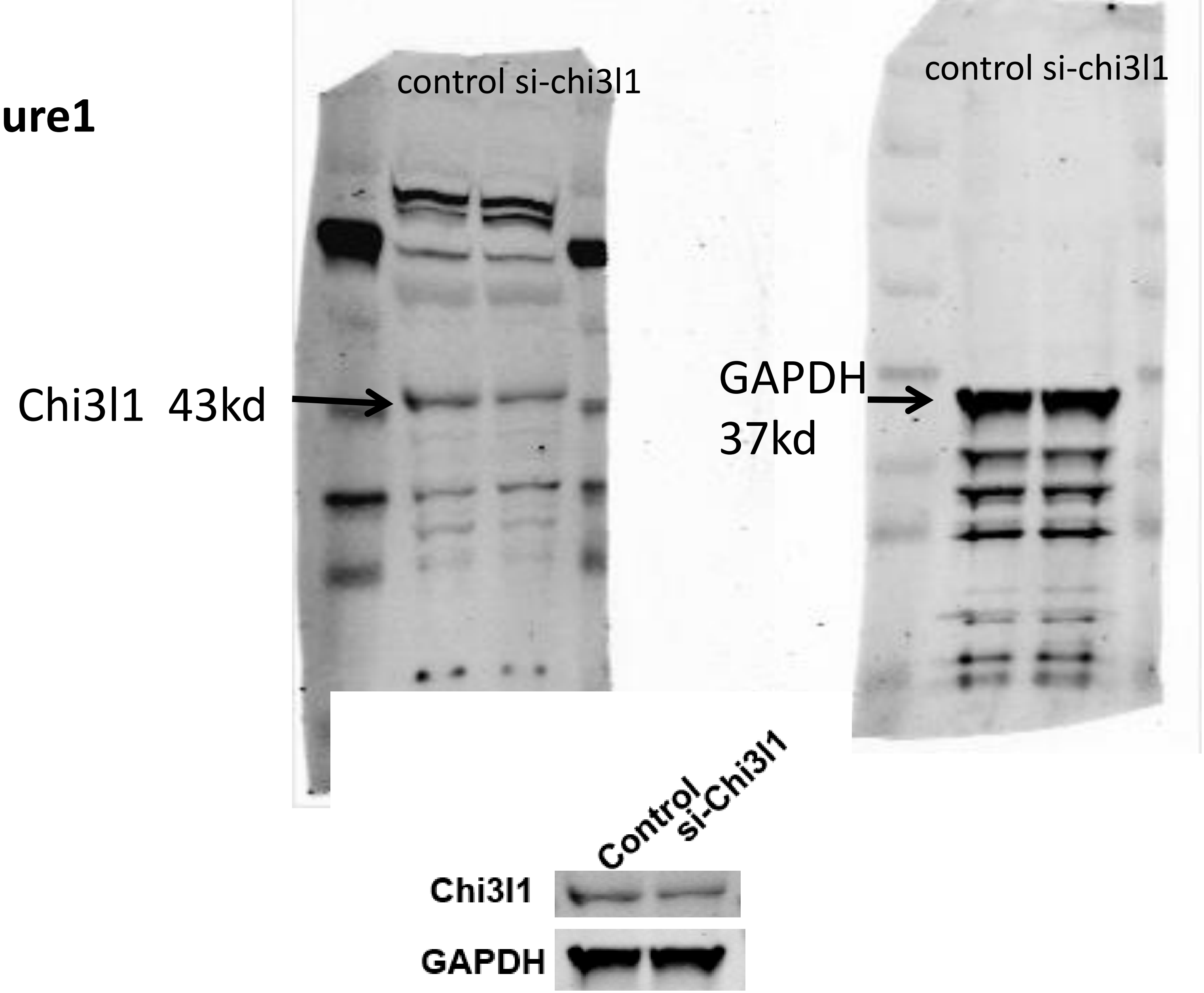
control si-chi3l1
Chi3l1 43kd
control si-chi3l1
GAPDH
37kd
Control
si-Chi3l1
Chi3l1
GAPDH

New Figure1

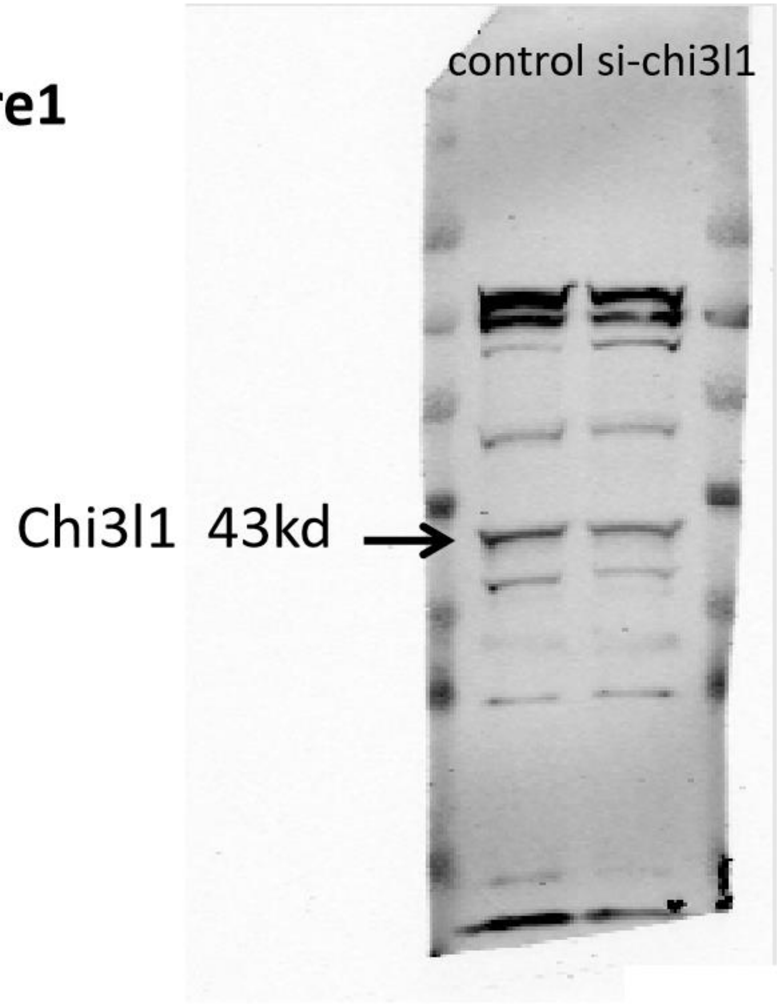

control si-chi3l1
Chi3l1 43kd

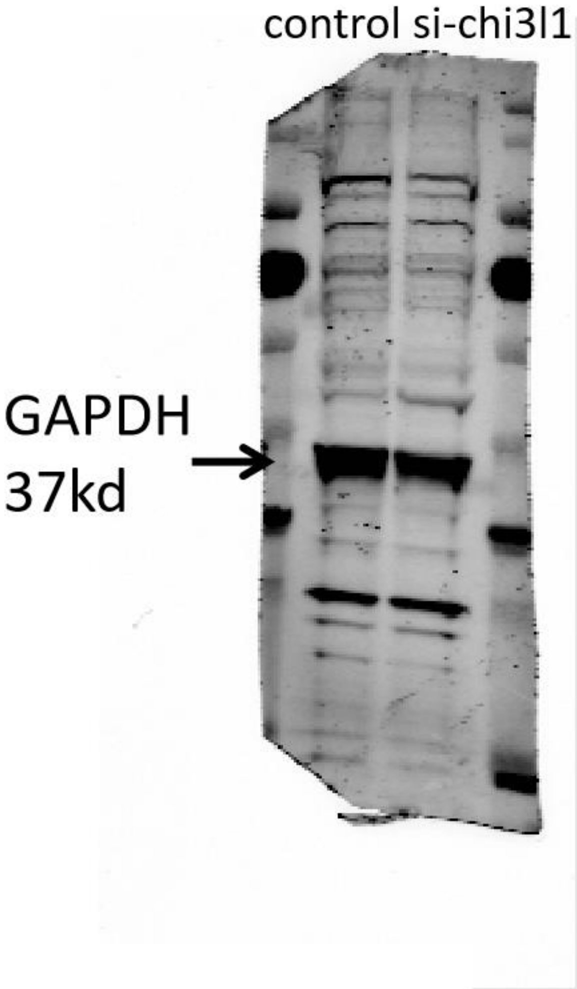

control si-chi3l1
GAPDH
37kd

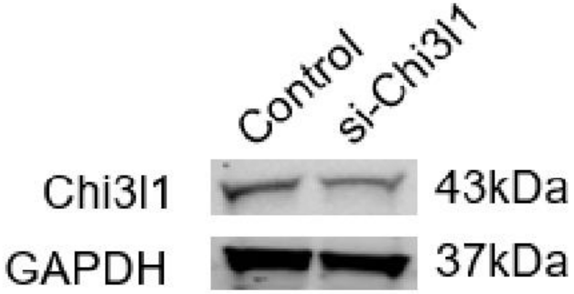

Control
si-Chi3l1
Chi3l1
43kDa
GAPDH
37kDa

Figure2.A

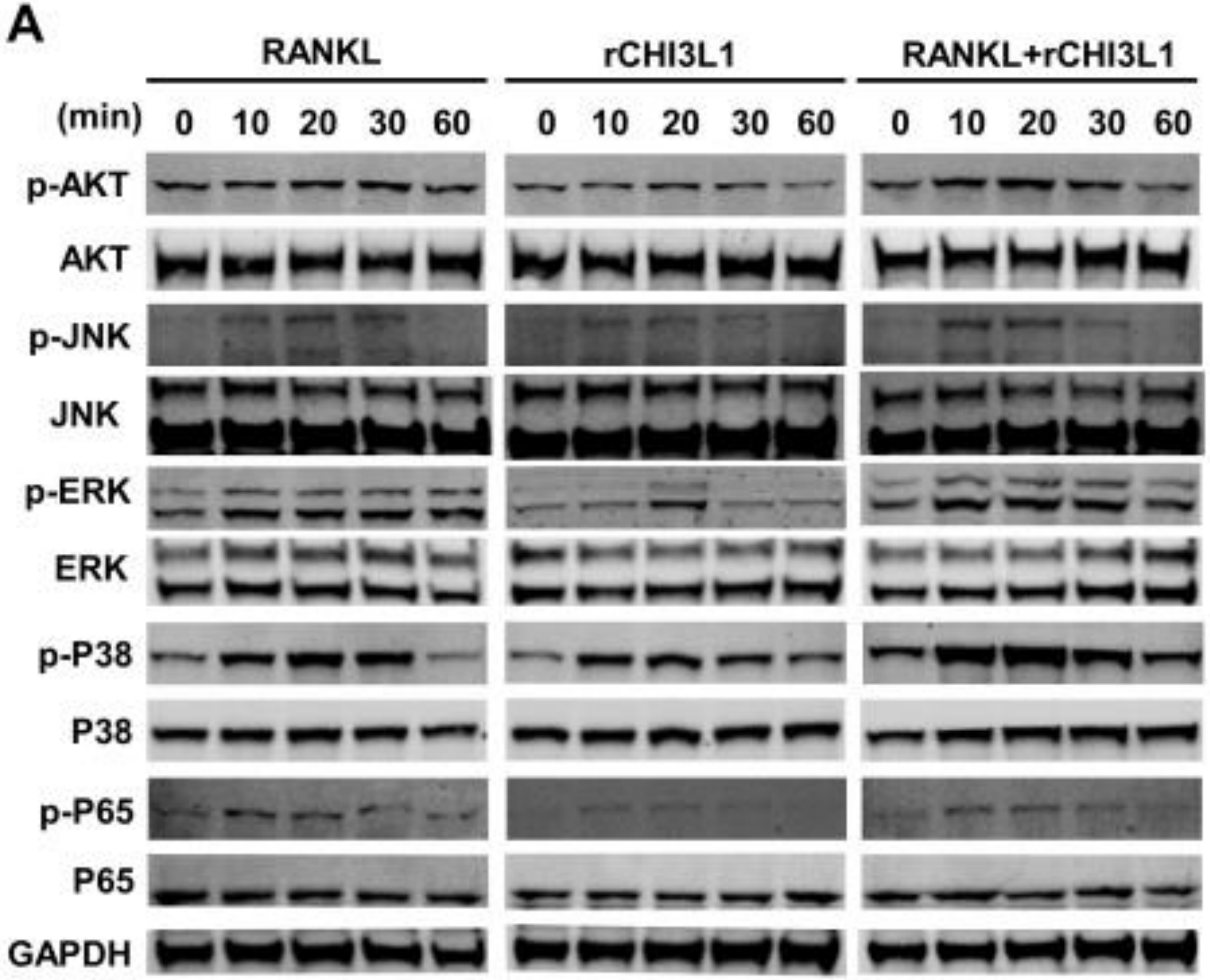
A
RANKL
rCHI3L1
RANKL+rCHI3L1
(min) 0 10 20 30 60 0 10 20 30 60 0 10 20 30 60
p-AKT
AKT
p-JNK
JNK
p-ERK
ERK
p-P38
P38
p-P65
P65
GAPDH

Figure 2.A

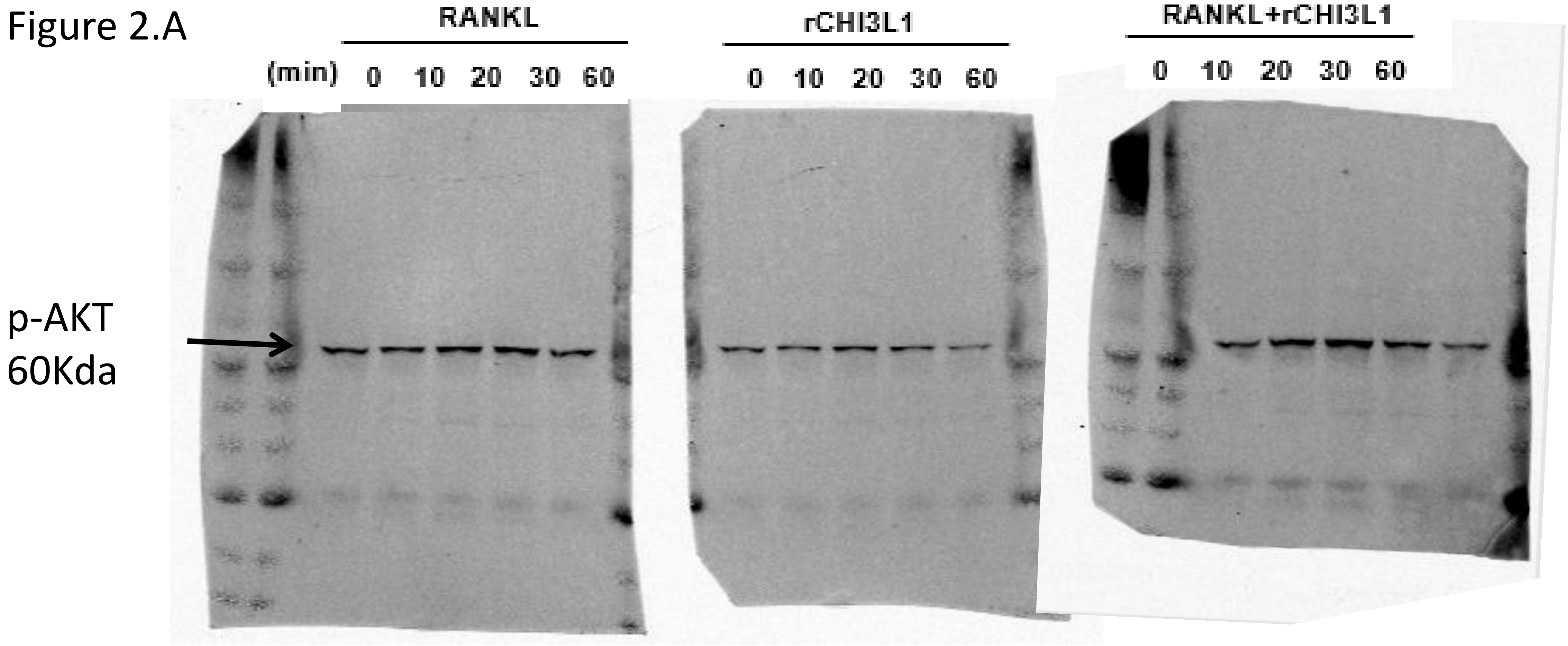
RANKL
(min) 0 10 20 30 60
rCHI3L1
0 10 20 30 60
RANKL+rCHI3L1
0 10 20 30 60
p-AKT
60Kda

Figure 2.A

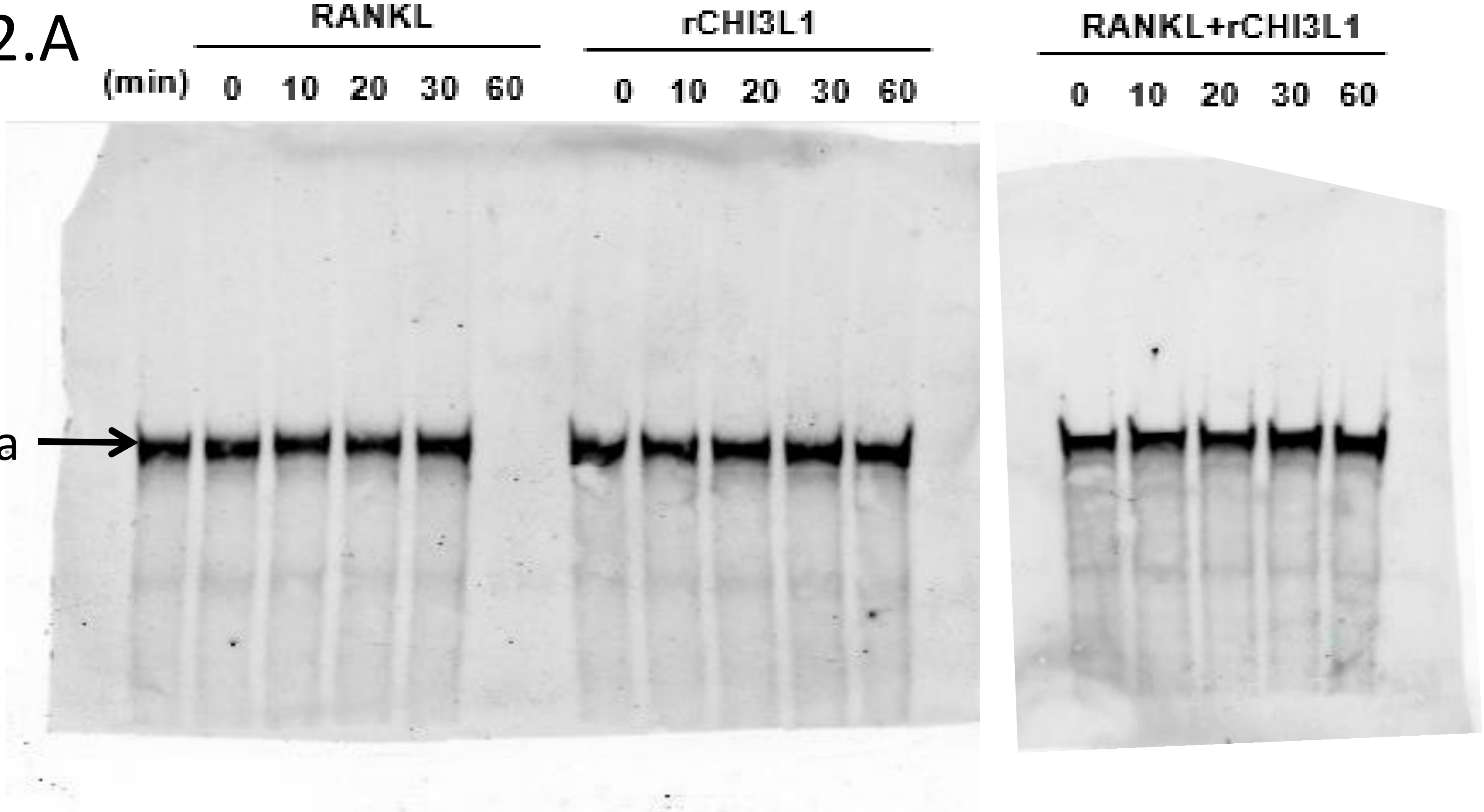
RANKL
rCHI3L1
RANKL+rCHI3L1
(min) 0 10 20 30 60
0 10 20 30 60
0 10 20 30 60
AKT 60Kda

Figure 2.A

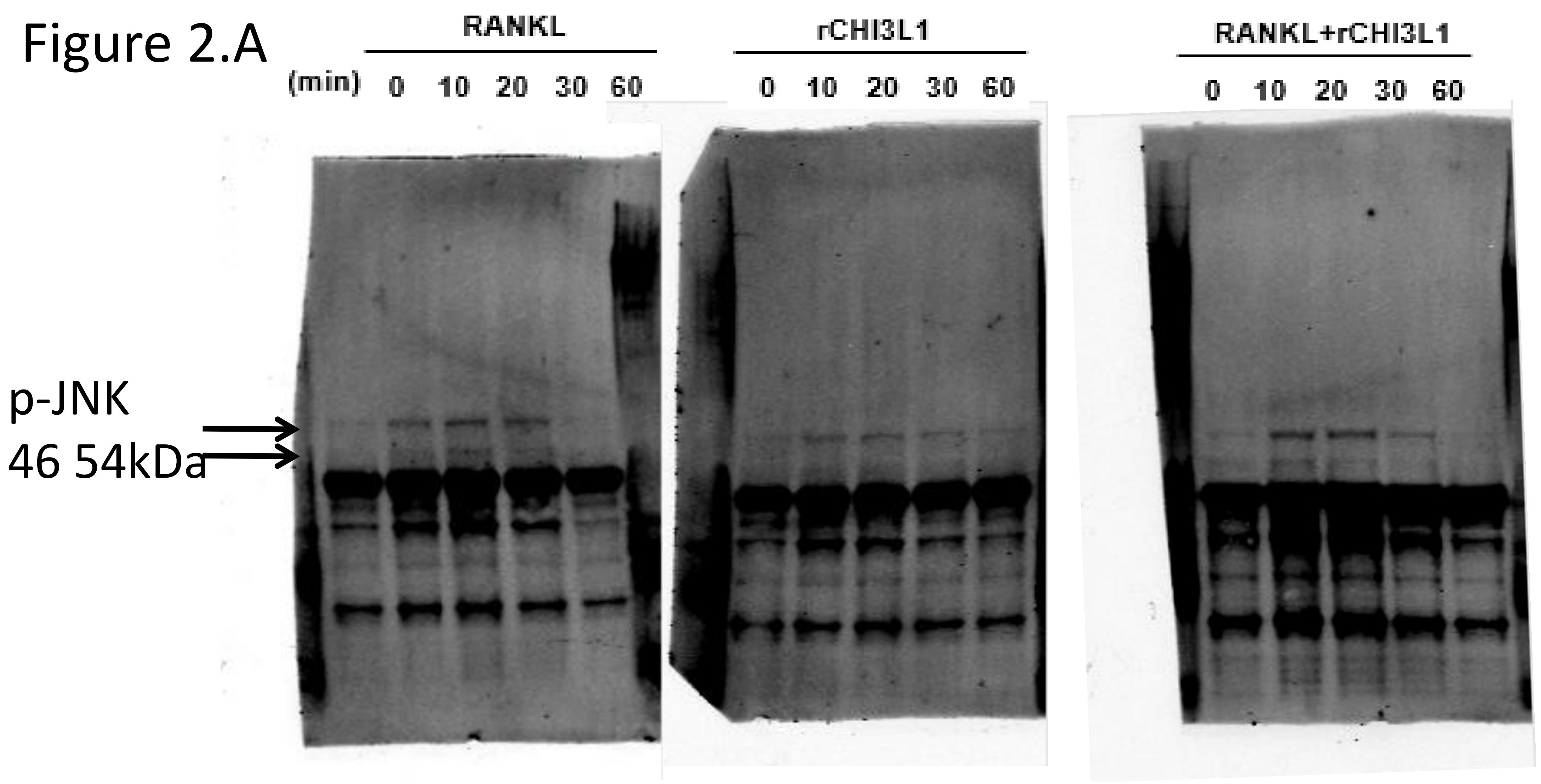

RANKL
rCHI3L1
RANKL+rCHI3L1
(min)
0 10 20 30 60
0 10 20 30 60
0 10 20 30 60
p-JNK
46 54kDa

Figure 2.A

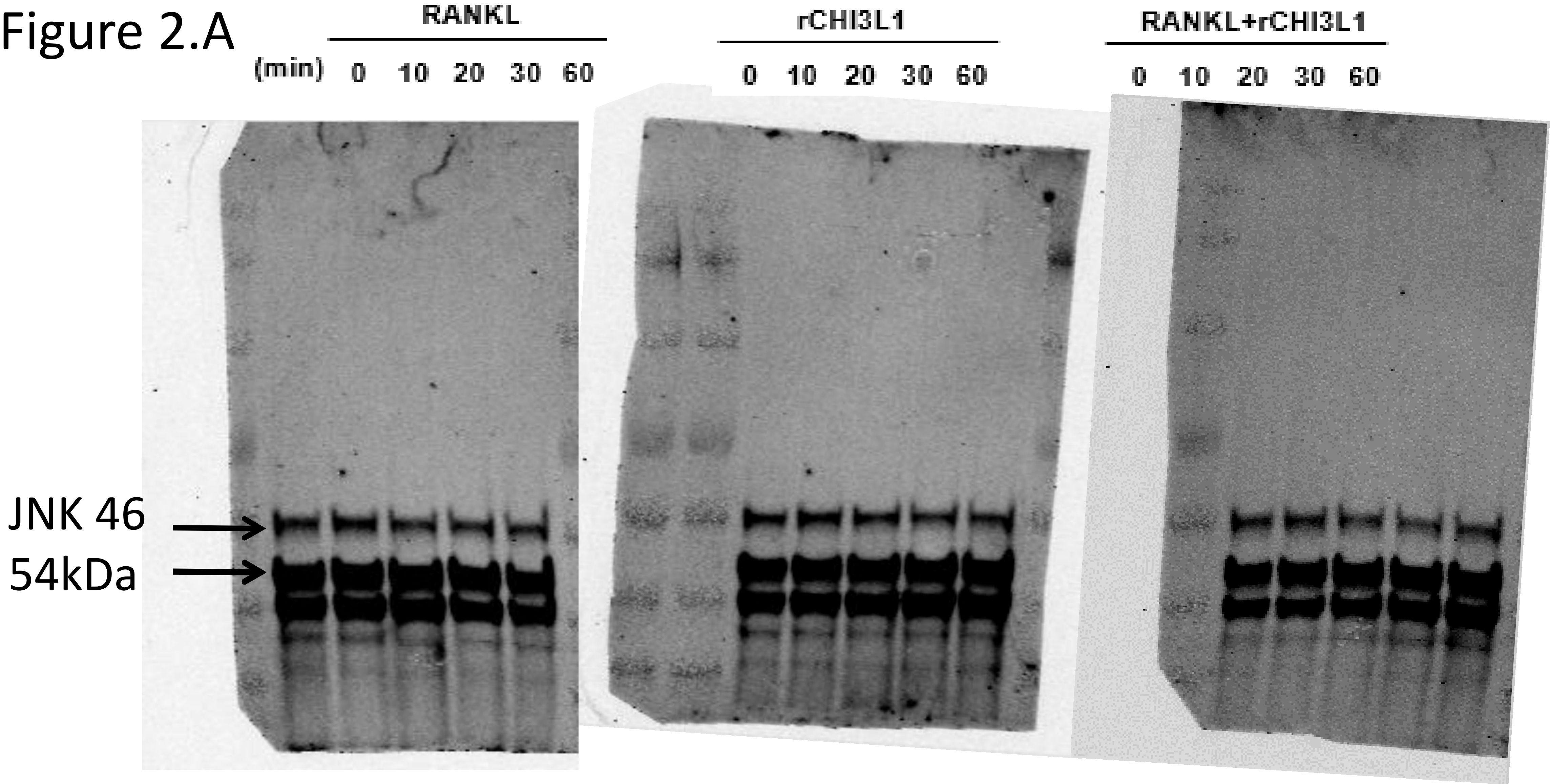
RANKL
(min) 0 10 20 30 60
rCHI3L1
0 10 20 30 60
RANKL+rCHI3L1
0 10 20 30 60
JNK 46
54kDa

Figure 2.A

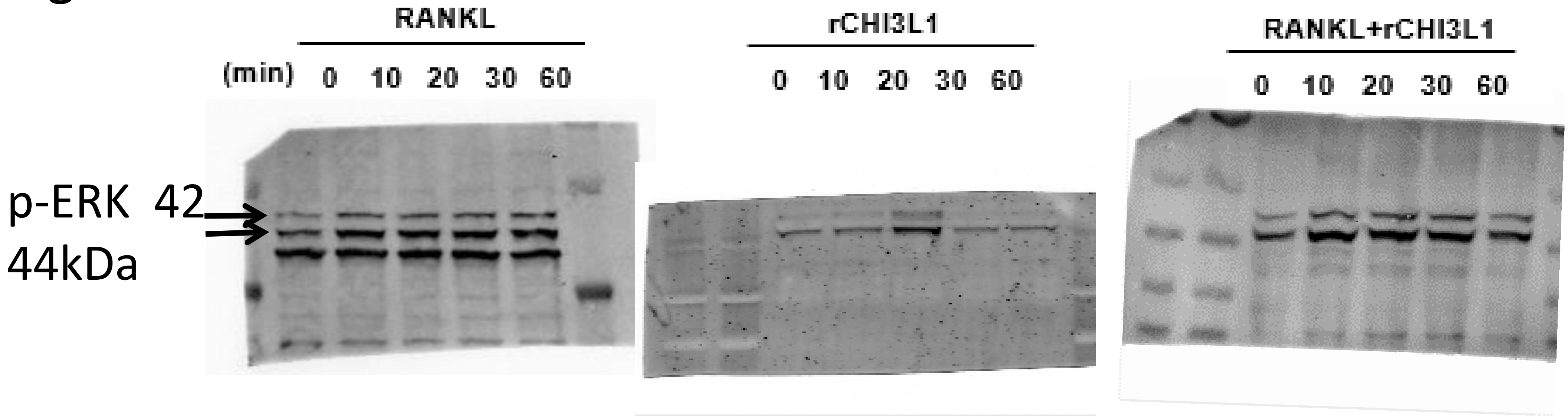
RANKL
(min) 0 10 20 30 60
rCHI3L1
0 10 20 30 60
RANKL+rCHI3L1
0 10 20 30 60
p-ERK 42
44kDa

Figure 2.A

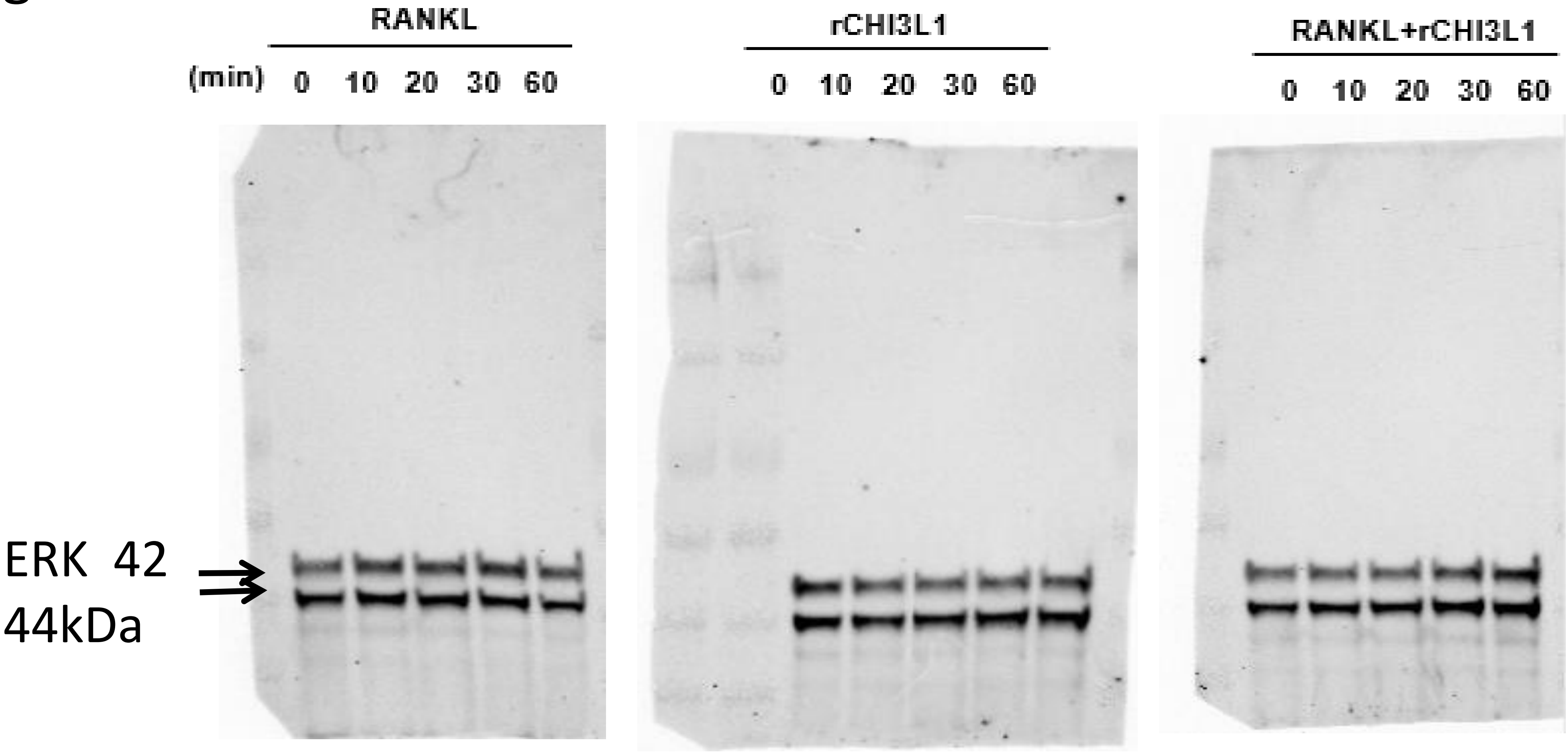

RANKL
rCHI3L1
RANKL+rCHI3L1
(min) 0 10 20 30 60
0 10 20 30 60
0 10 20 30 60
ERK 42
44kDa

Figure 2.A

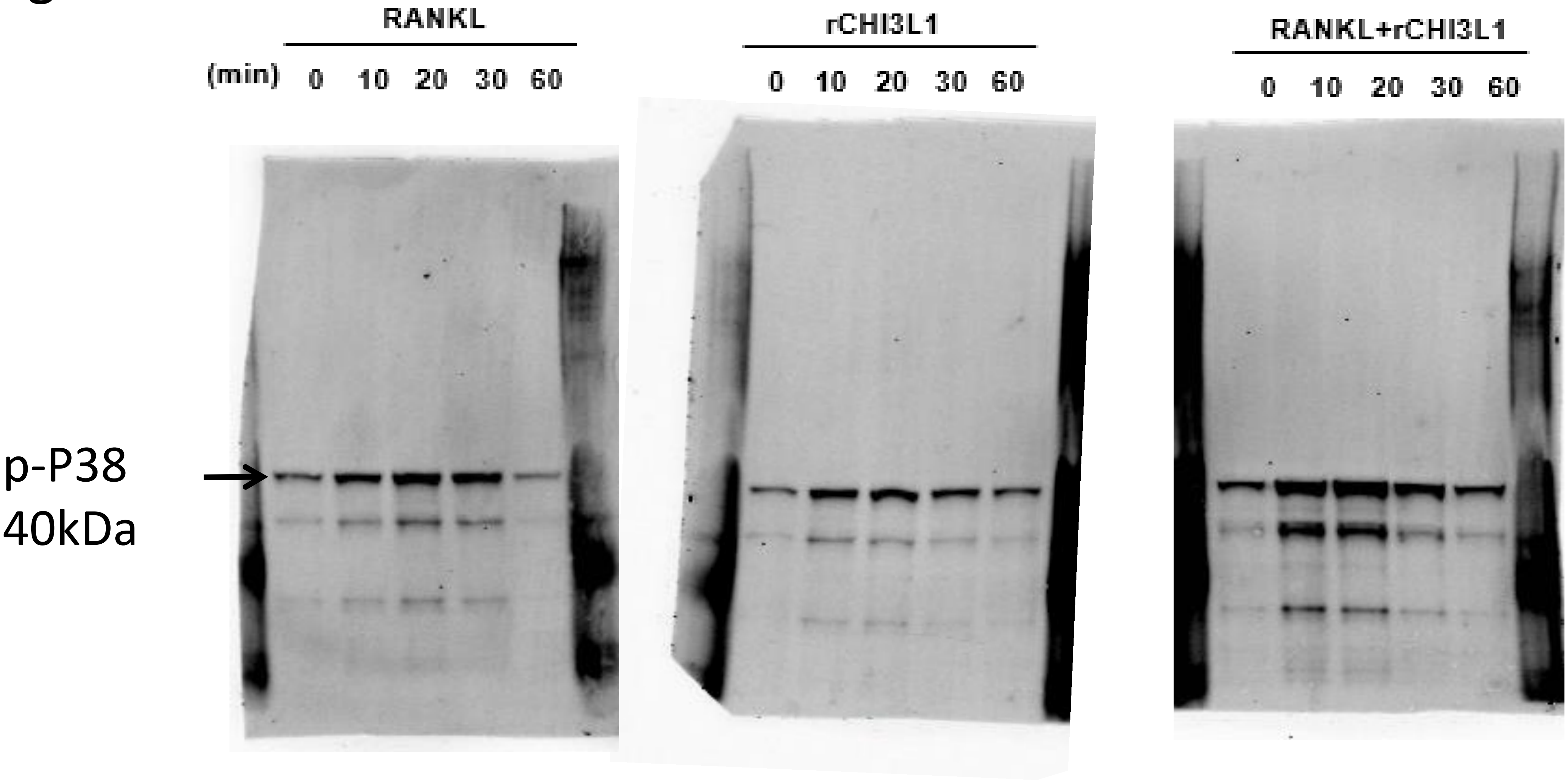
RANKL
(min) 0 10 20 30 60
rCHI3L1
0 10 20 30 60
RANKL+rCHI3L1
0 10 20 30 60
p-P38
40kDa

Figure 2.A

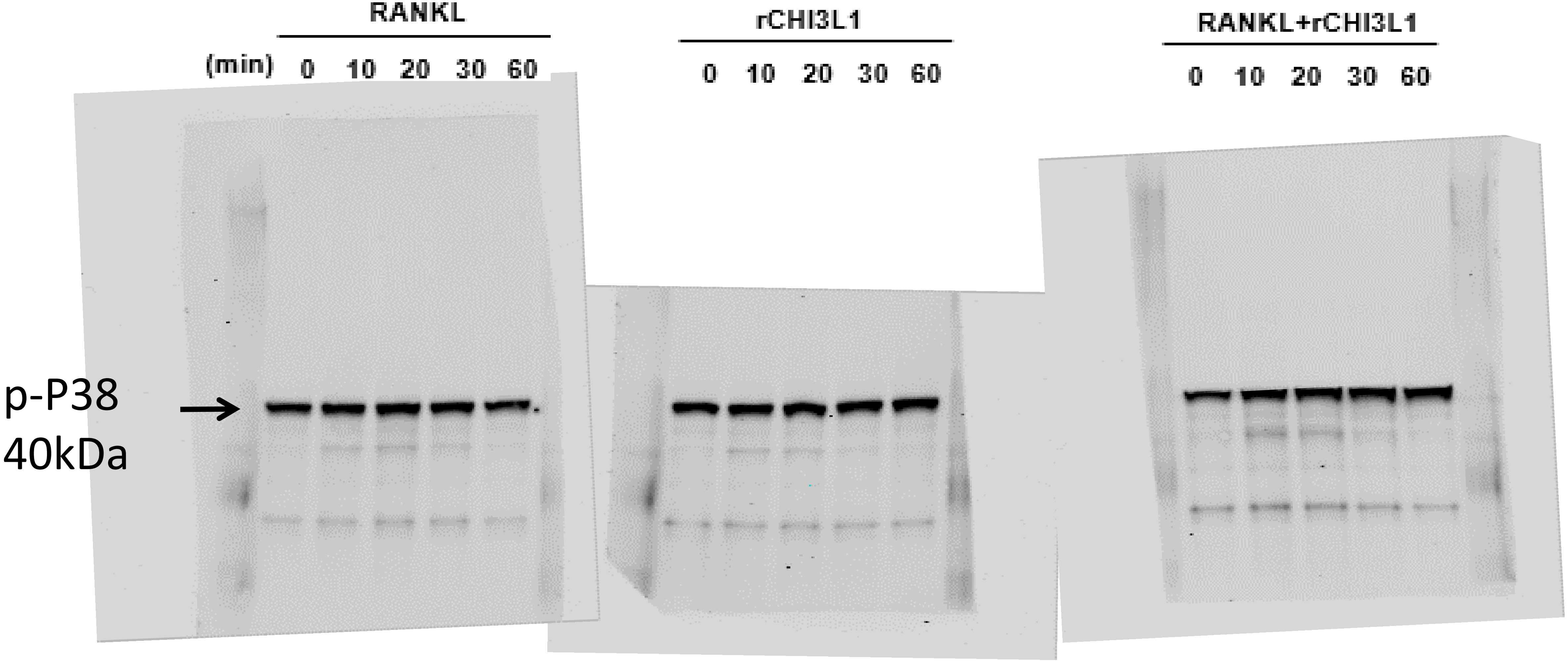
RANKL
rCHI3L1
RANKL+rCHI3L1
(min) 0 10 20 30 60
0 10 20 30 60
0 10 20 30 60
p-P38
40kDa

Figure 2.A

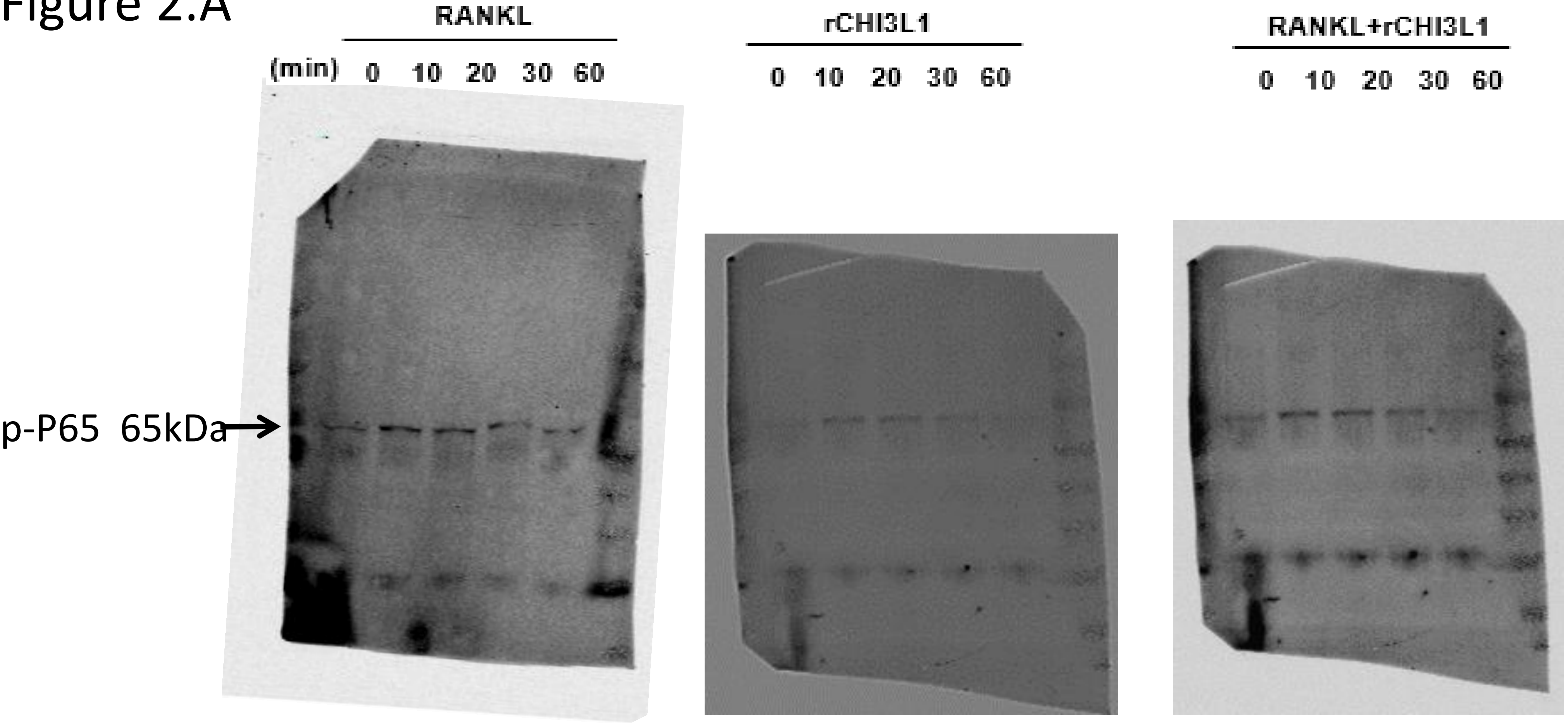
RANKL
(min) 0 10 20 30 60
rCHI3L1
0 10 20 30 60
RANKL+rCHI3L1
0 10 20 30 60
p-P65 65kDa

Figure 2.A

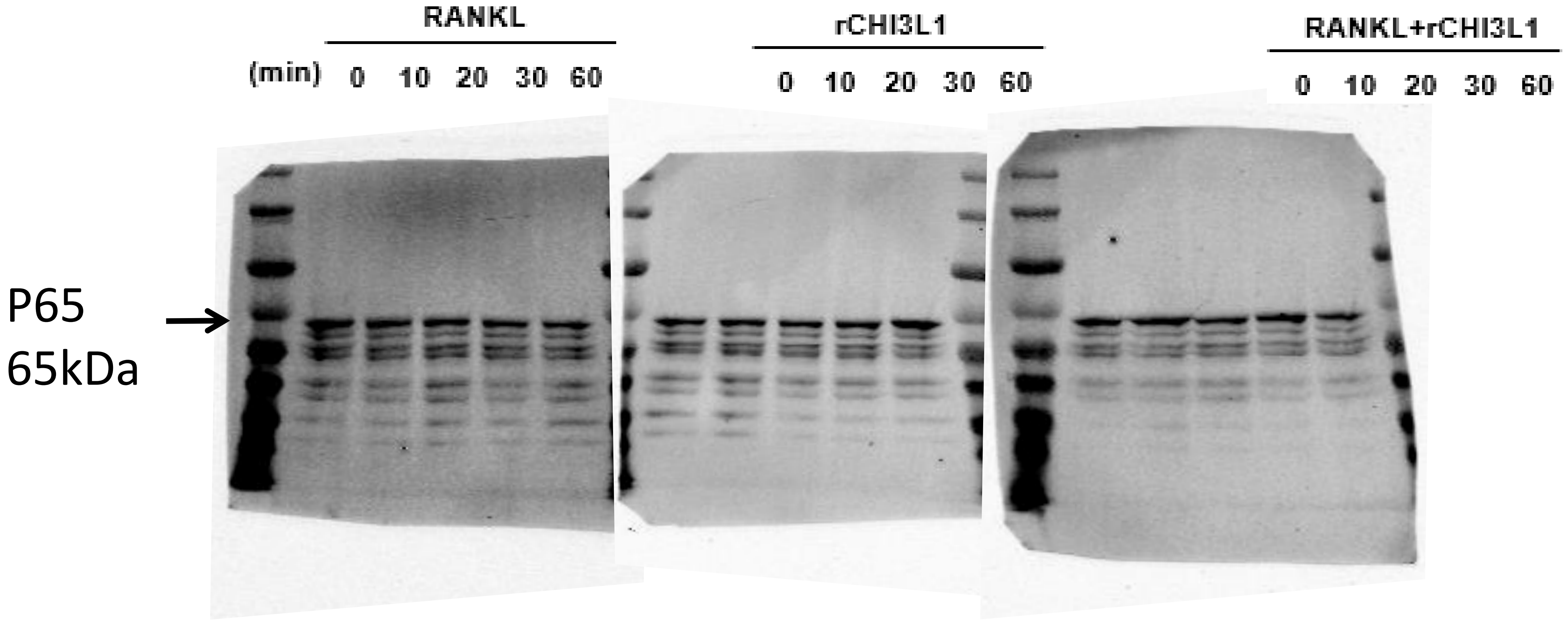
RANKL
(min) 0 10 20 30 60
rCHI3L1
0 10 20 30 60
RANKL+rCHI3L1
0 10 20 30 60
P65
65kDa

Figure 2.A

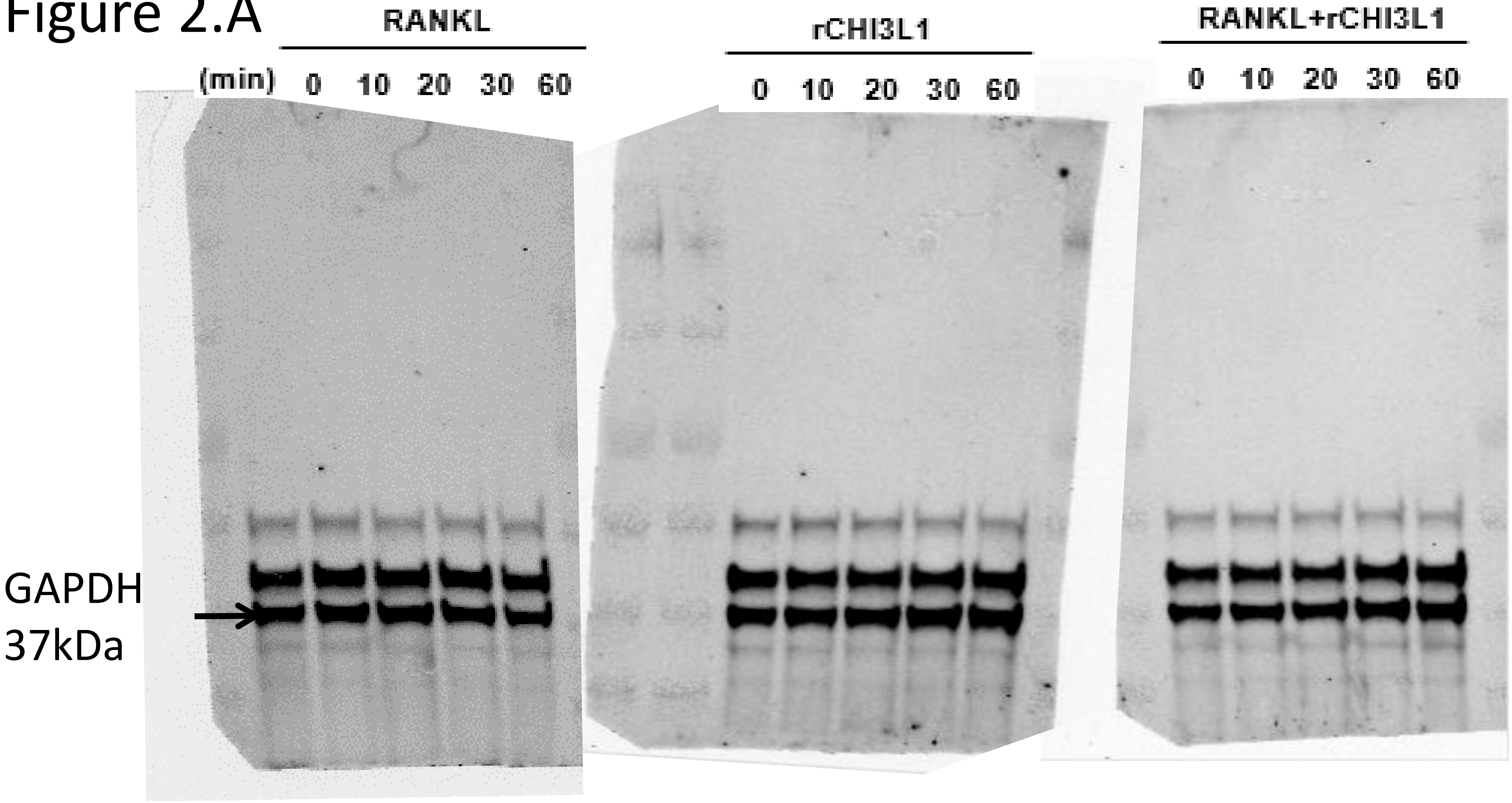
RANKL
rCHI3L1
RANKL+rCHI3L1
(min)
0 10 20 30 60
0 10 20 30 60
0 10 20 30 60
GAPDH
37kDa

Figure2.C

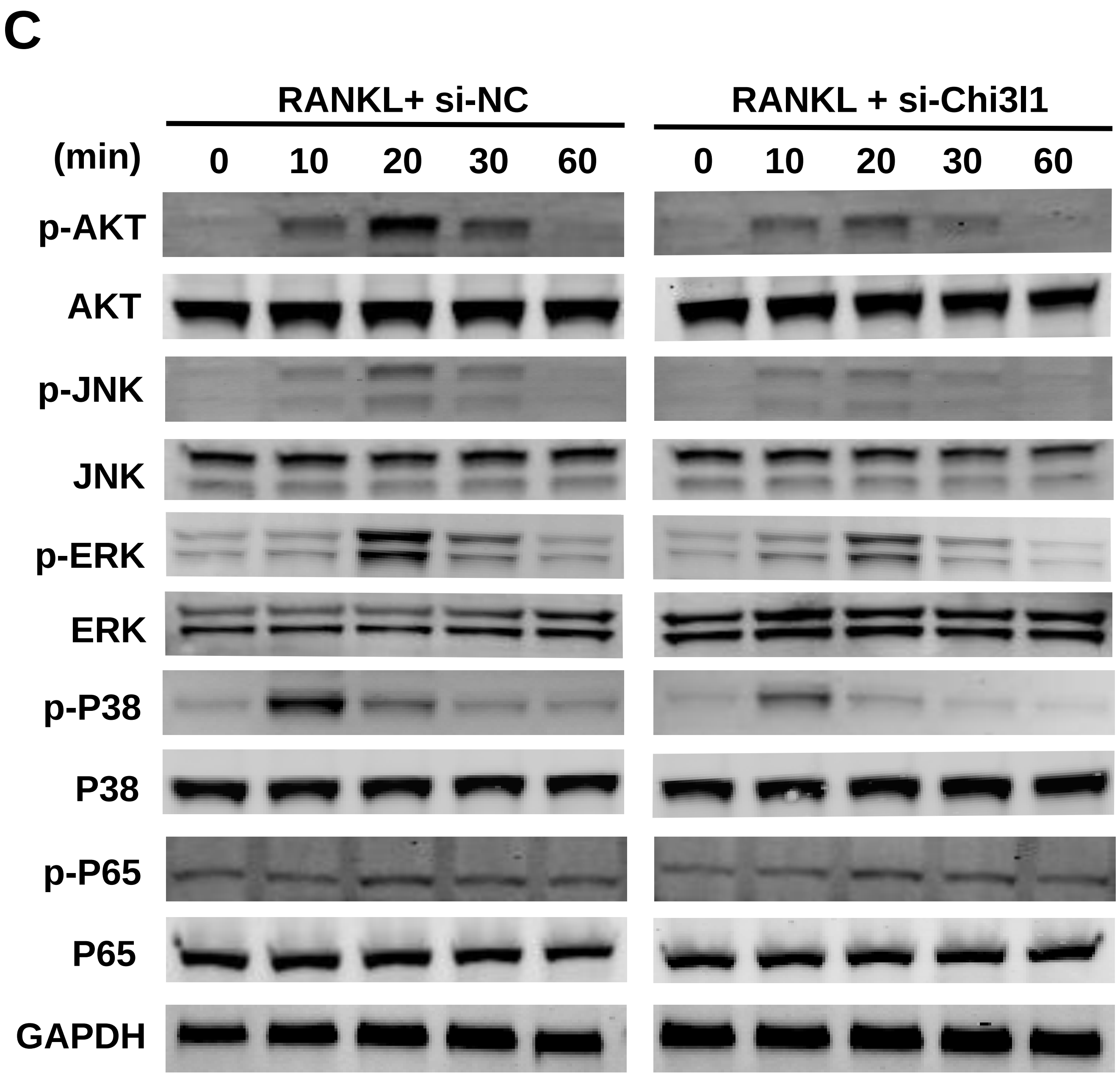
C
RANKL+ si-NC
RANKL + si-Chi3l1
(min)
0
10
20
30
60
0
10
20
30
60
p-AKT
AKT
p-JNK
JNK
p-ERK
ERK
p-P38
P38
p-P65
P65
GAPDH

Figure2.C

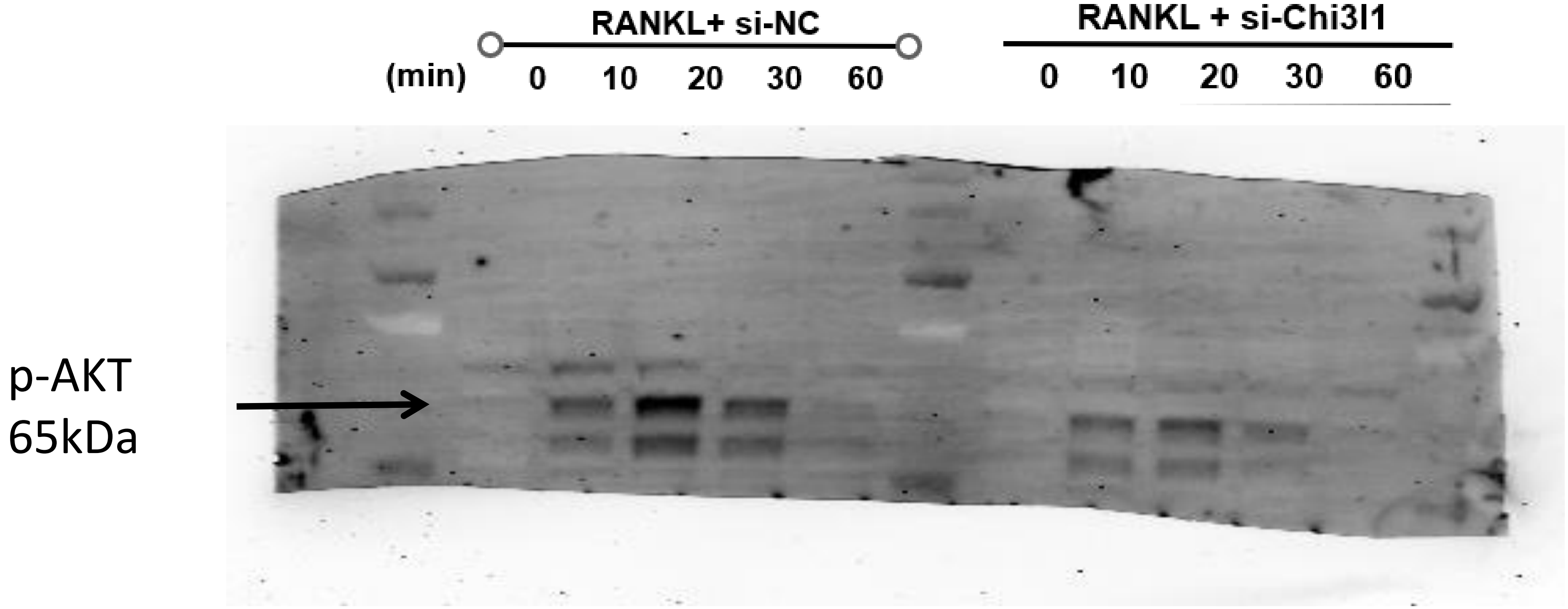

RANKL+ si-NC
RANKL + si-Chi3l1
(min) 0 10 20 30 60
0 10 20 30 60
p-AKT
65kDa

Figure2.C

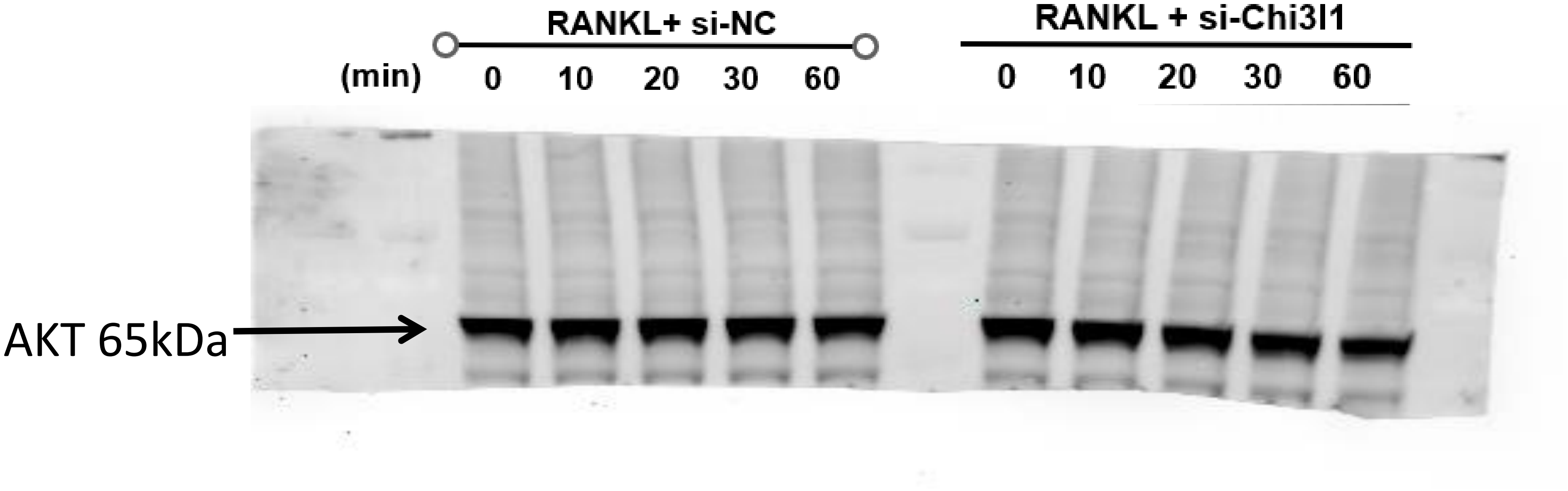
RANKL+ si-NC
RANKL + si-Chi3l1
(min) 0 10 20 30 60
0 10 20 30 60
AKT 65kDa

Figure2.C

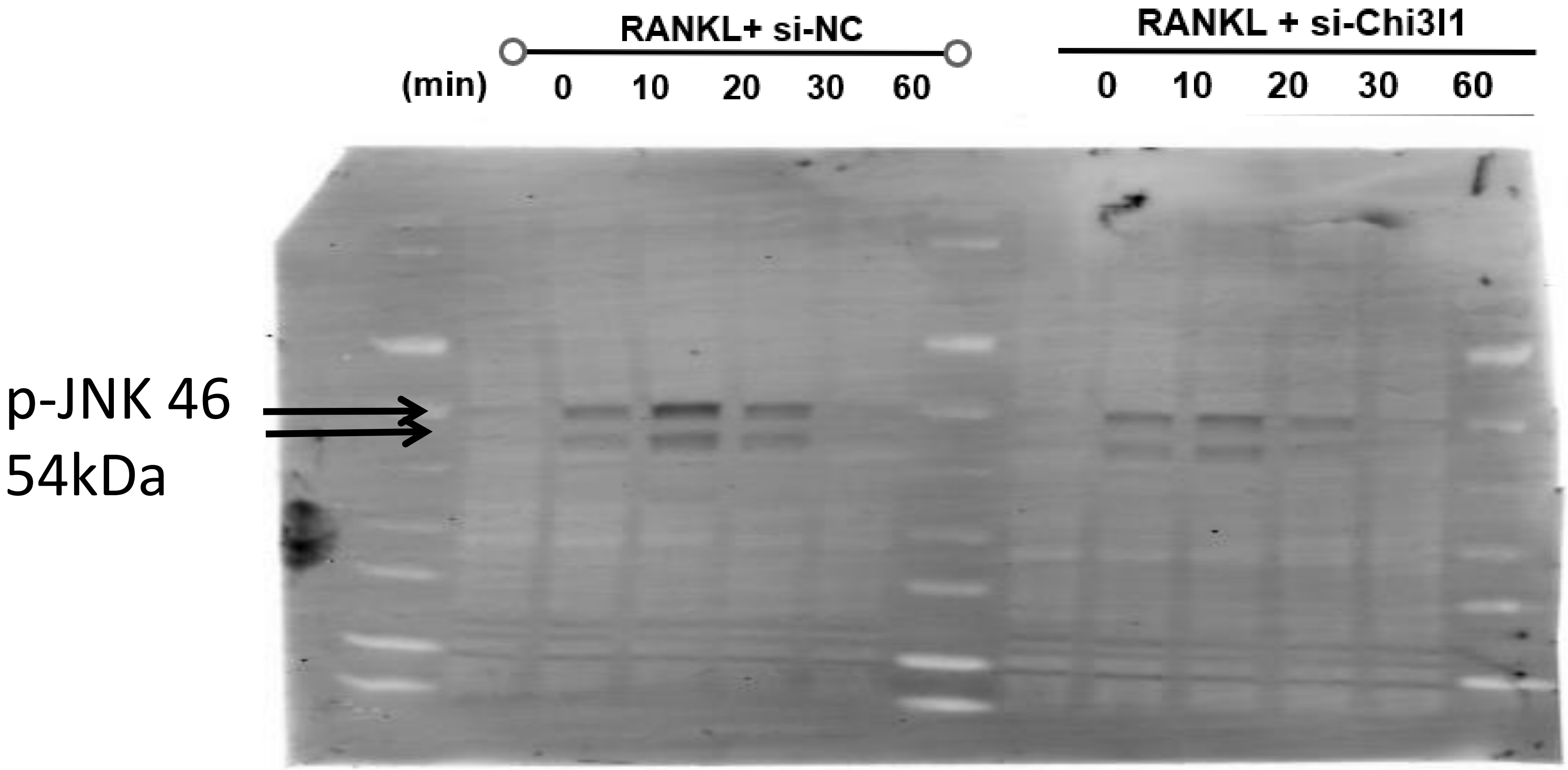
RANKL+ si-NC
RANKL + si-Chi3l1
(min)
0 10 20 30 60
0 10 20 30 60
p-JNK 46
54kDa

Figure2.C

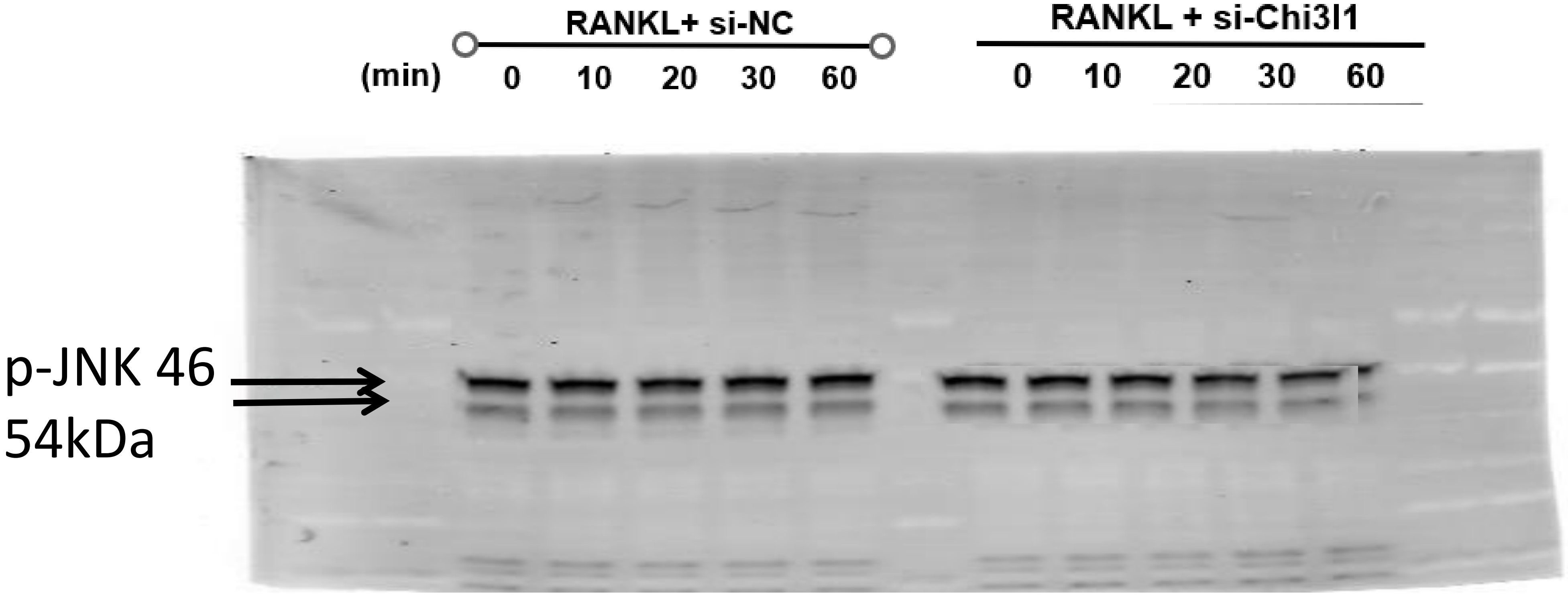
RANKL+ si-NC
RANKL + si-Chi3l1
(min)
0
10
20
30
60
0
10
20
30
60
p-JNK 46
54kDa

Figure2.C

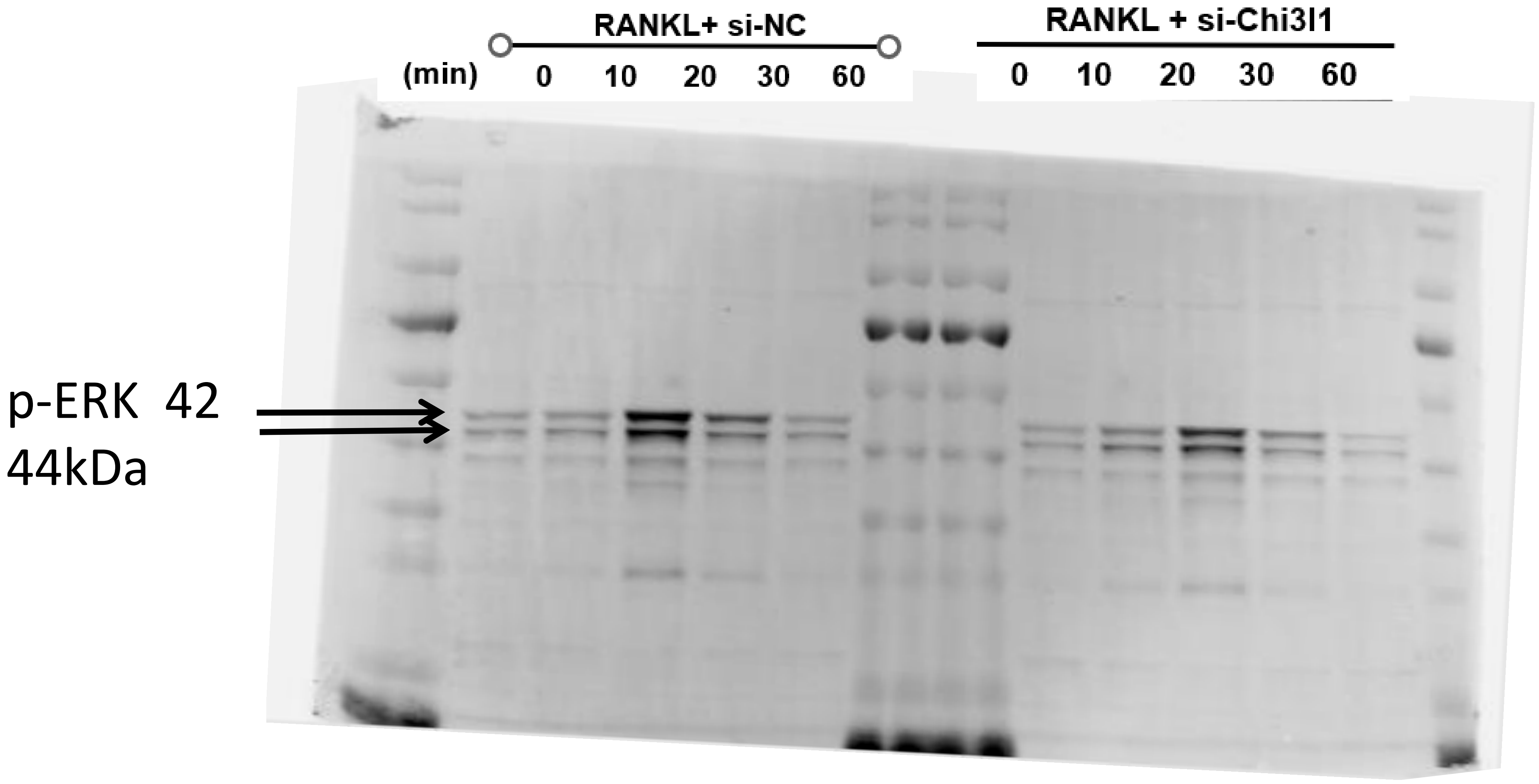
RANKL+ si-NC
RANKL + si-Chi3l1
(min)
0
10
20
30
60
0
10
20
30
60
p-ERK 42
44kDa

Figure2.C

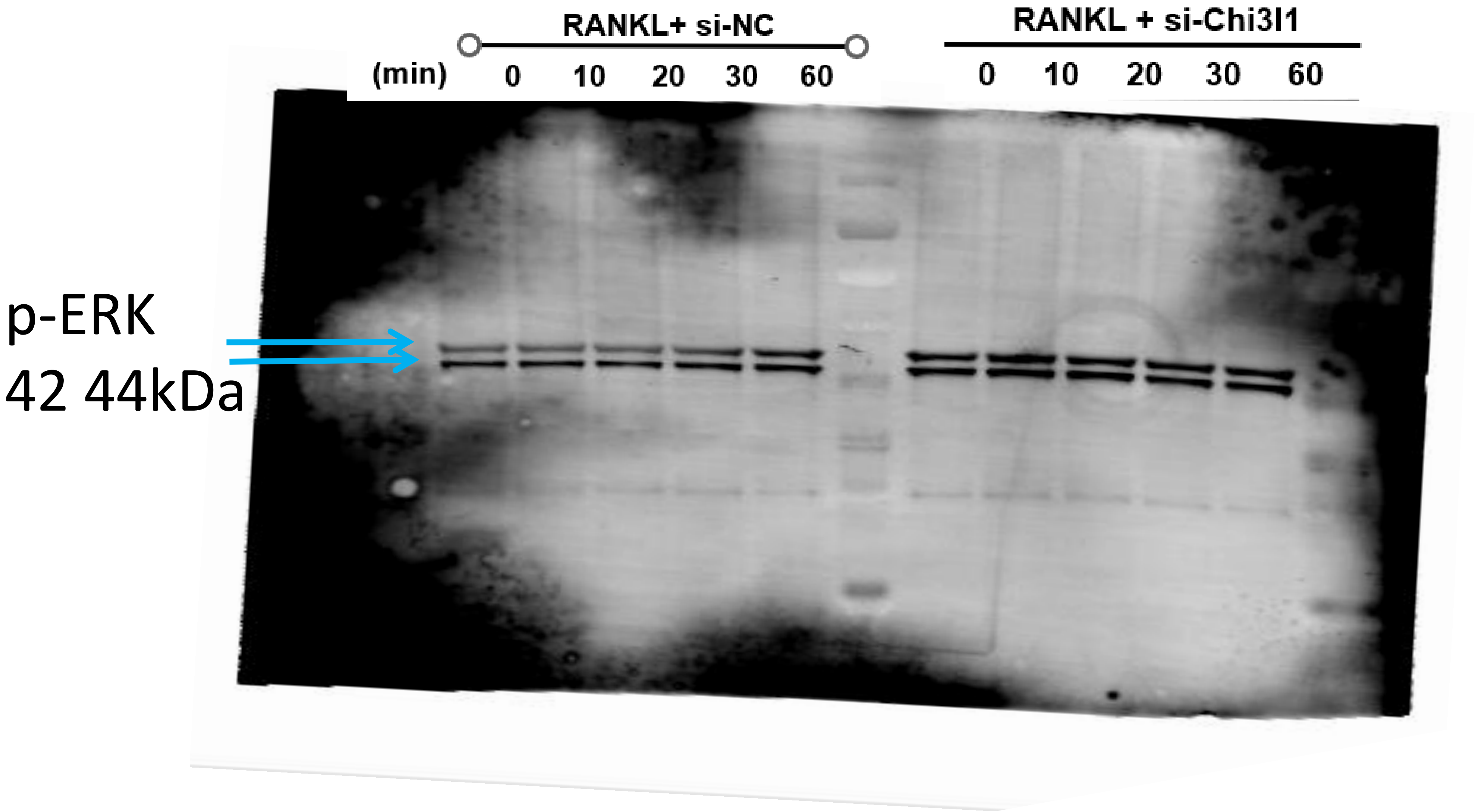
RANKL+ si-NC
RANKL + si-Chi3l1
(min)
0 10 20 30 60
0 10 20 30 60
p-ERK
42 44kDa

Figure2.C

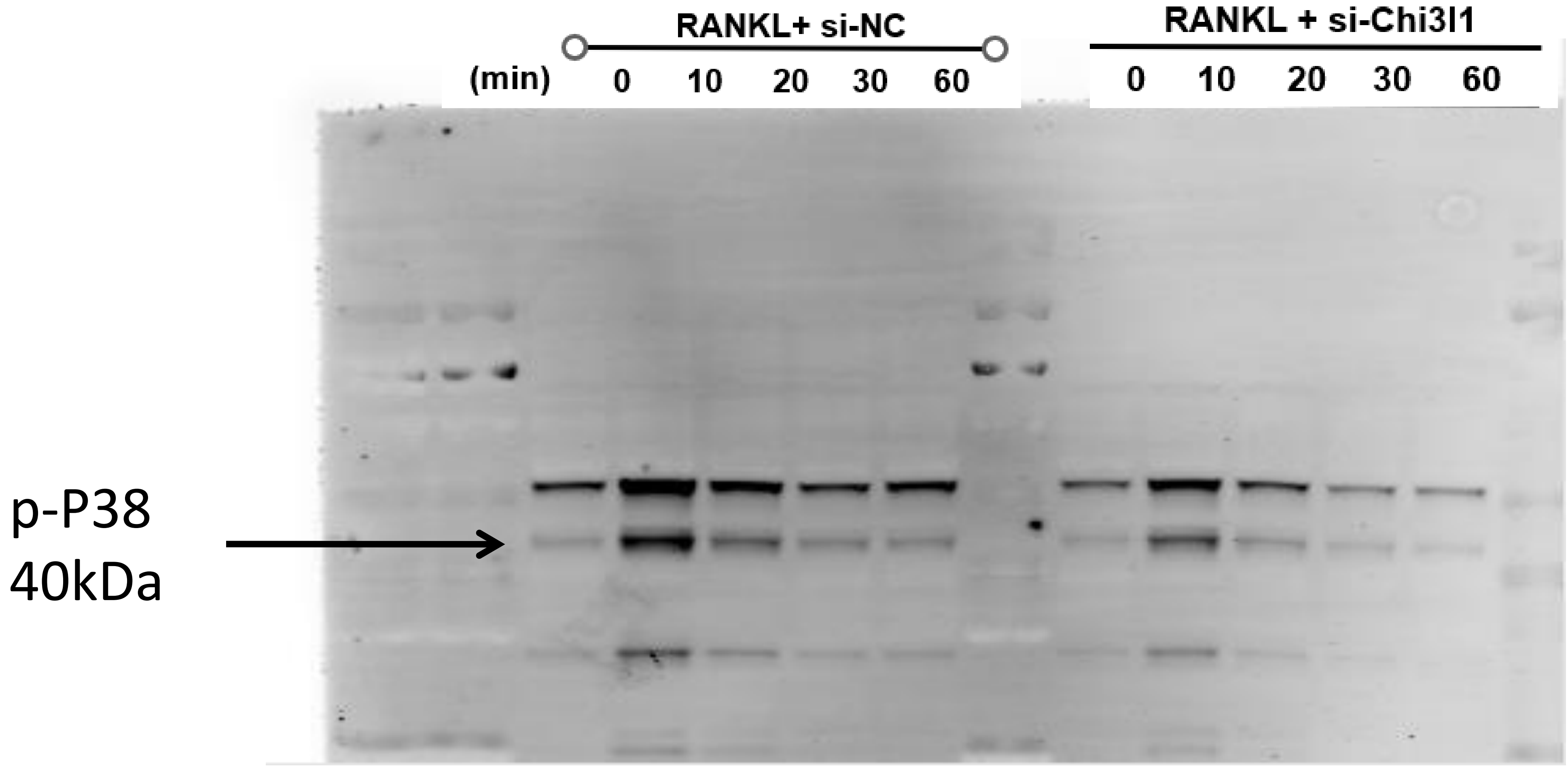

RANKL+ si-NC
RANKL + si-Chi3l1
(min)
0
10
20
30
60
0
10
20
30
60
p-P38
40kDa

Figure2.C

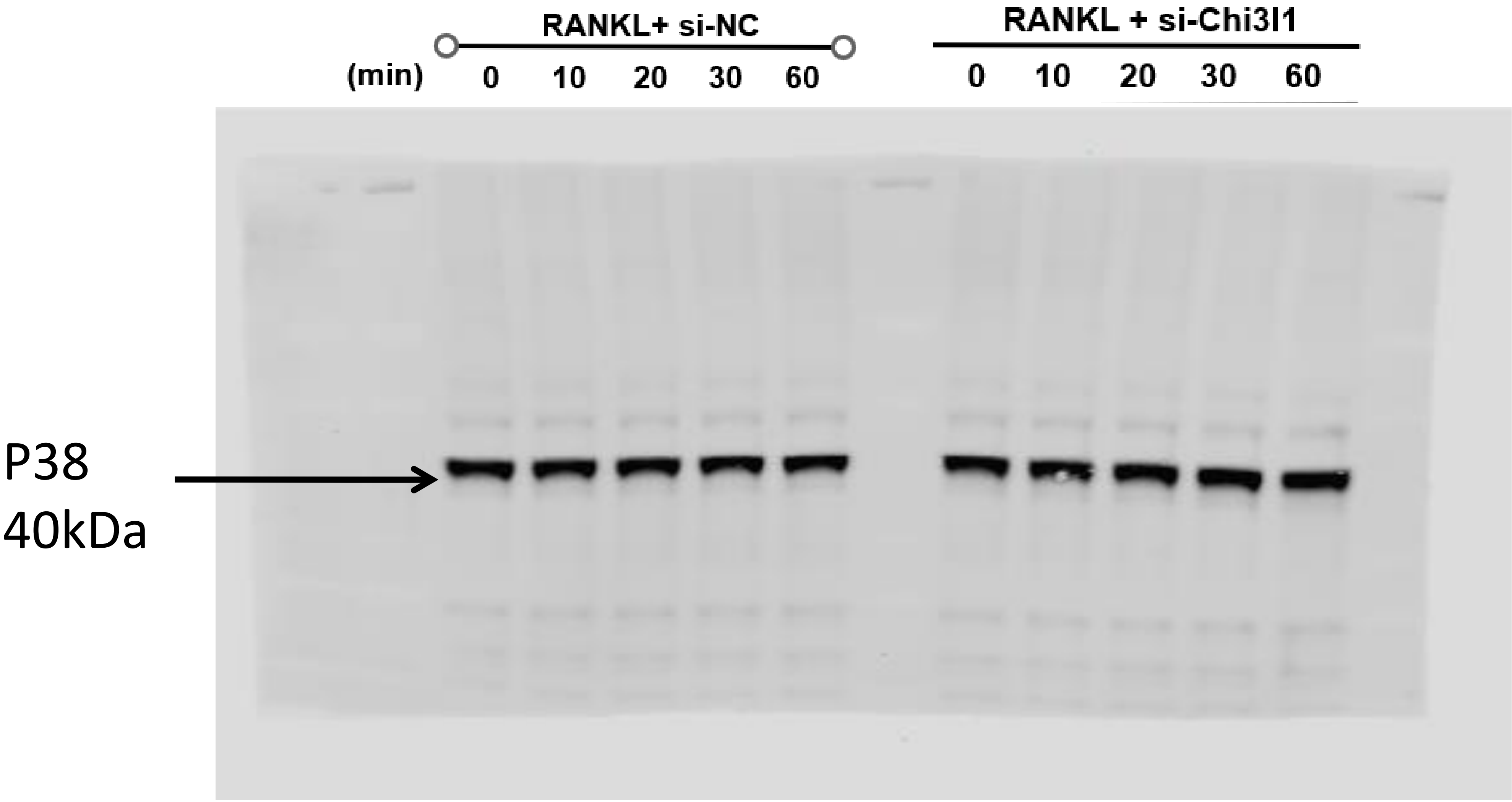

RANKL+ si-NC
RANKL + si-Chi3l1
(min)
0
10
20
30
60
0
10
20
30
60
P38
40kDa

Figure2.C

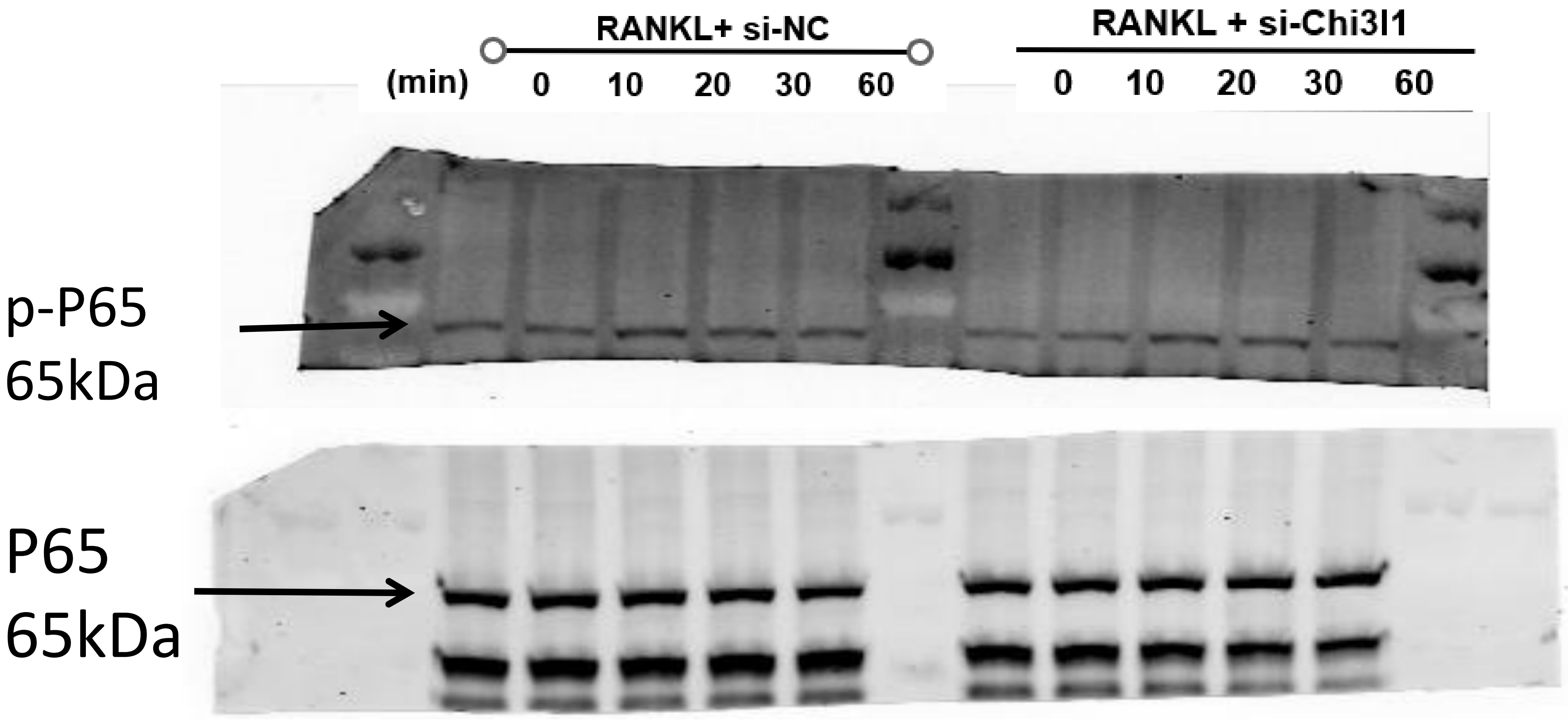
RANKL+ si-NC
RANKL + si-Chi3l1
(min)
0 10 20 30 60
0 10 20 30 60
p-P65
65kDa
P65
65kDa

Figure2.C

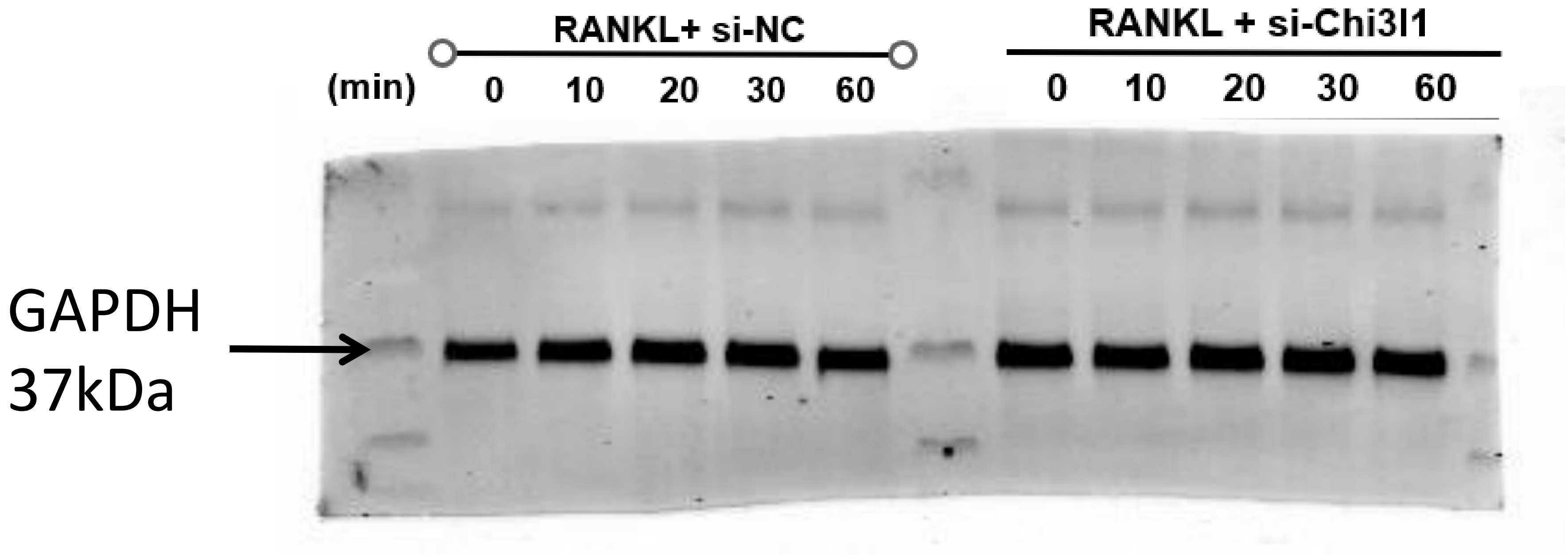
RANKL+ si-NC
RANKL + si-Chi3l1
(min)
0
10
20
30
60
0
10
20
30
60
GAPDH
37kDa

figure 3C

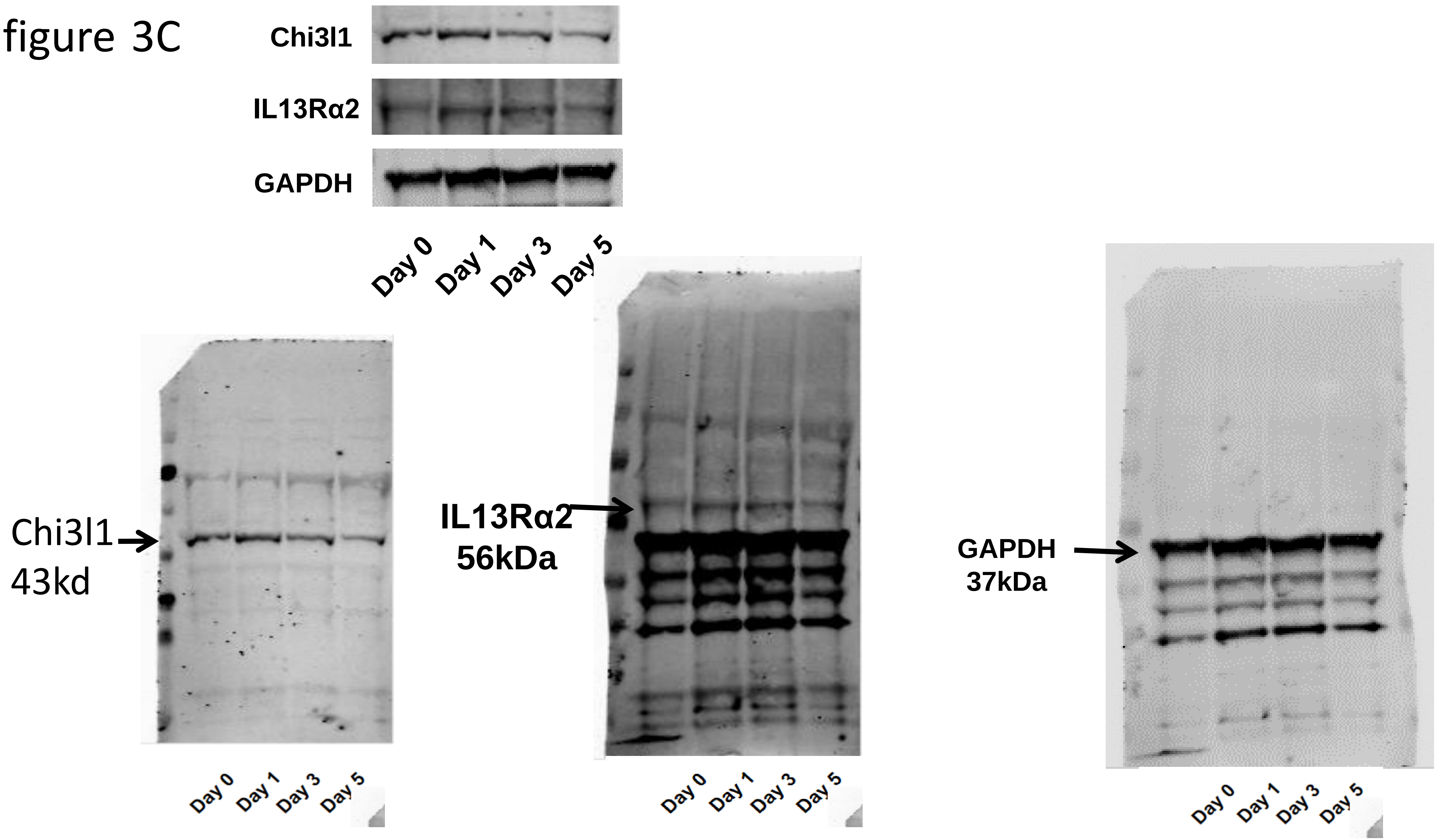
Chi3l1
IL13Rα2
GAPDH
Day 0
Day 1
Day 3
Day 5
Chi3l1
43kd
IL13Rα2
56kDa
GAPDH
37kDa

figure 3F

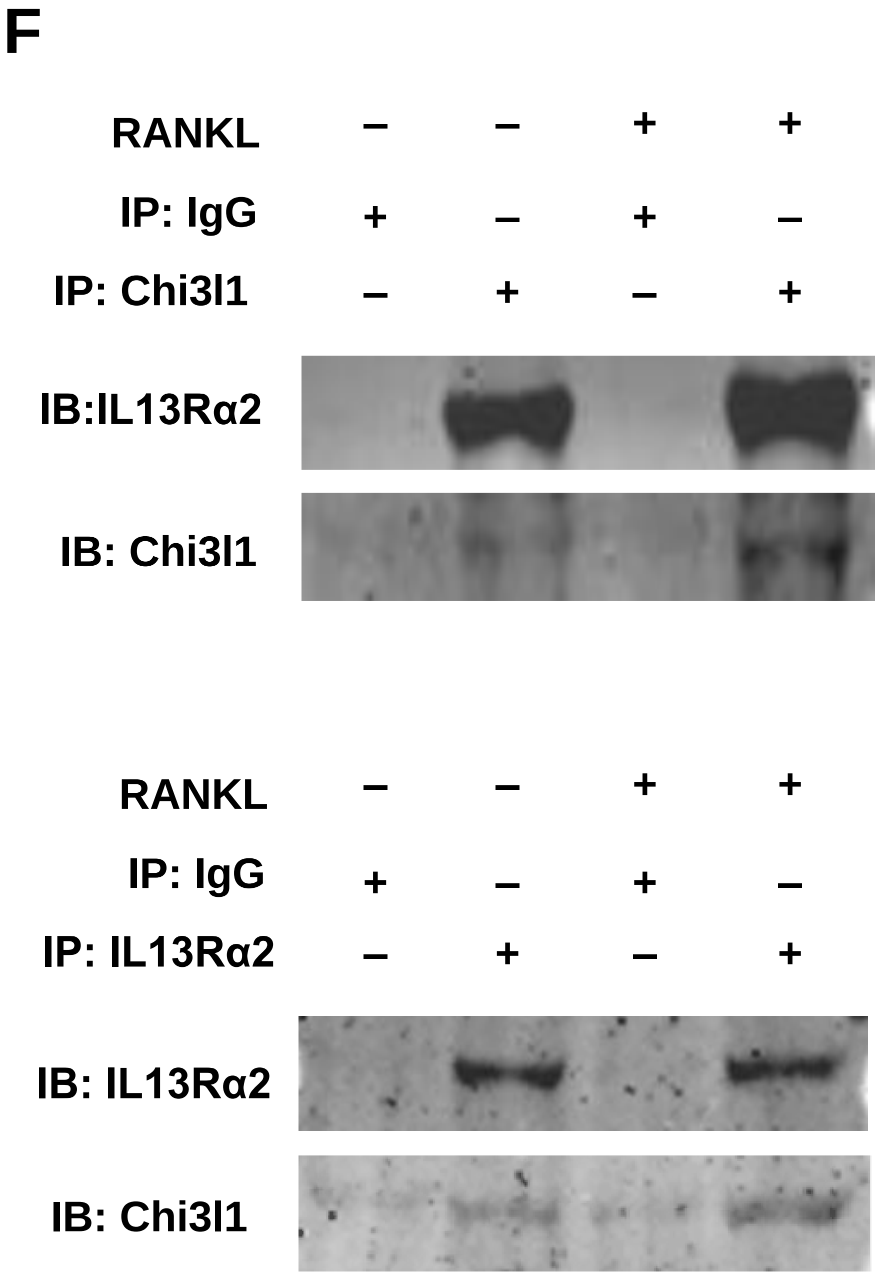
F
RANKL − − + +
IP: IgG + − + −
IP: Chi3l1 − + − +
IB:IL13Rα2
IB: Chi3l1
RANKL − − + +
IP: IgG + − + −
IP: IL13Rα2 − + − +
IB: IL13Rα2
IB: Chi3l1

Figure 3F

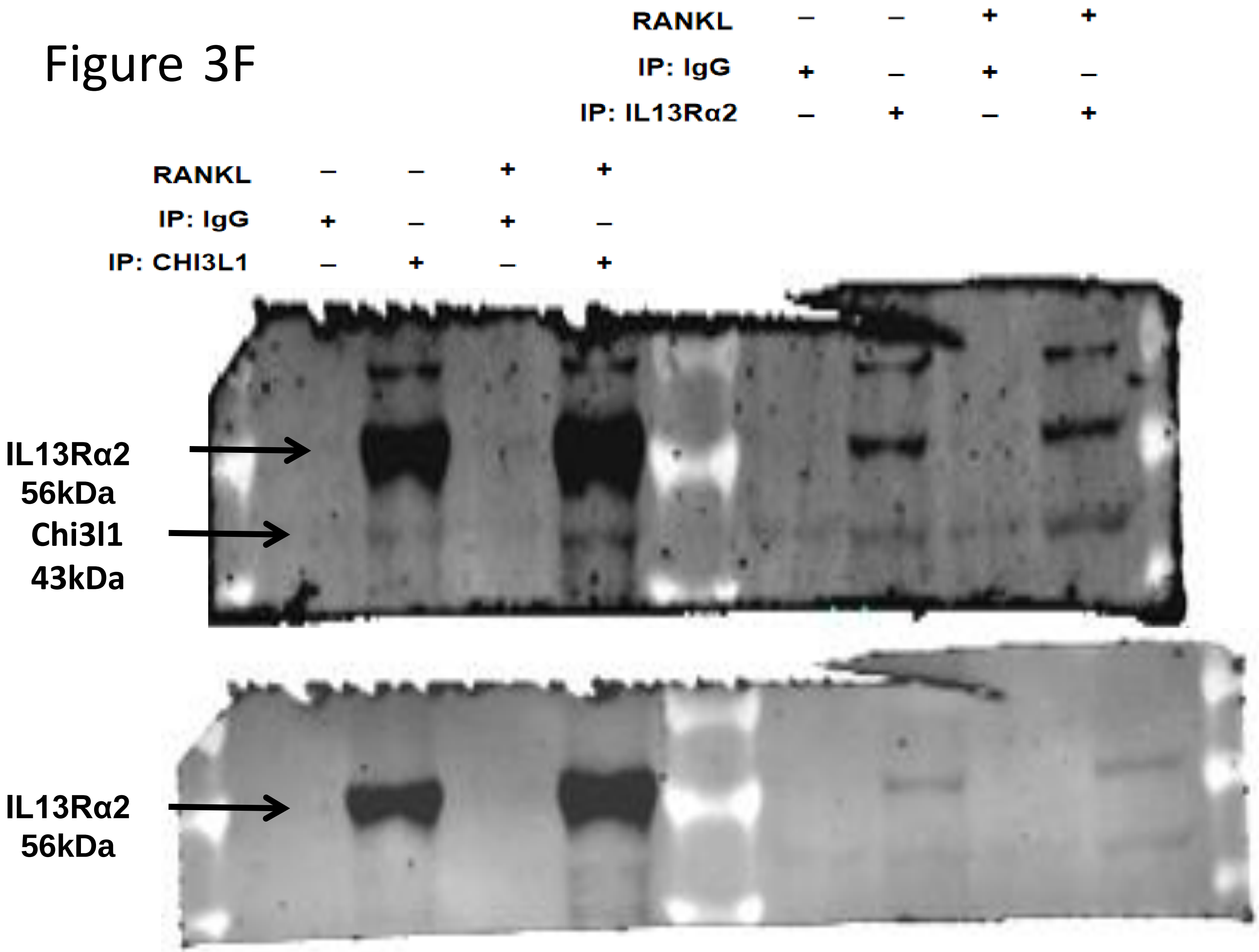

RANKL – – + +
IP: IgG + – + –
IP: IL13Rα2 – + – +
RANKL – – + +
IP: IgG + – + –
IP: CHI3L1 – + – +
IL13Rα2 56kDa
Chi3l1 43kDa
IL13Rα2 56kDa

Figure5A

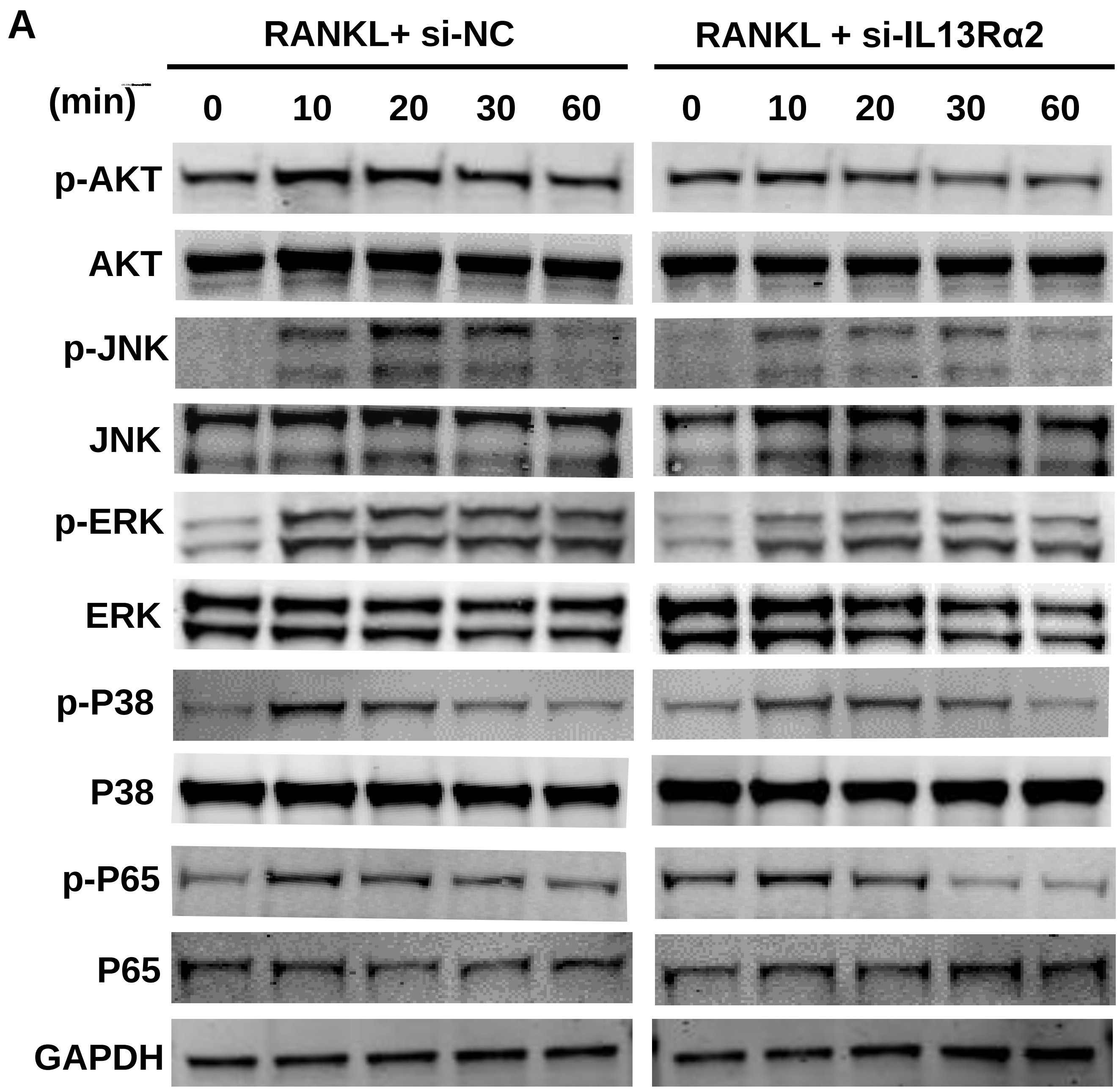
A
RANKL+ si-NC
RANKL + si-IL13Rα2
(min)
0 10 20 30 60
0 10 20 30 60
p-AKT
AKT
p-JNK
JNK
p-ERK
ERK
p-P38
P38
p-P65
P65
GAPDH

Figure5A

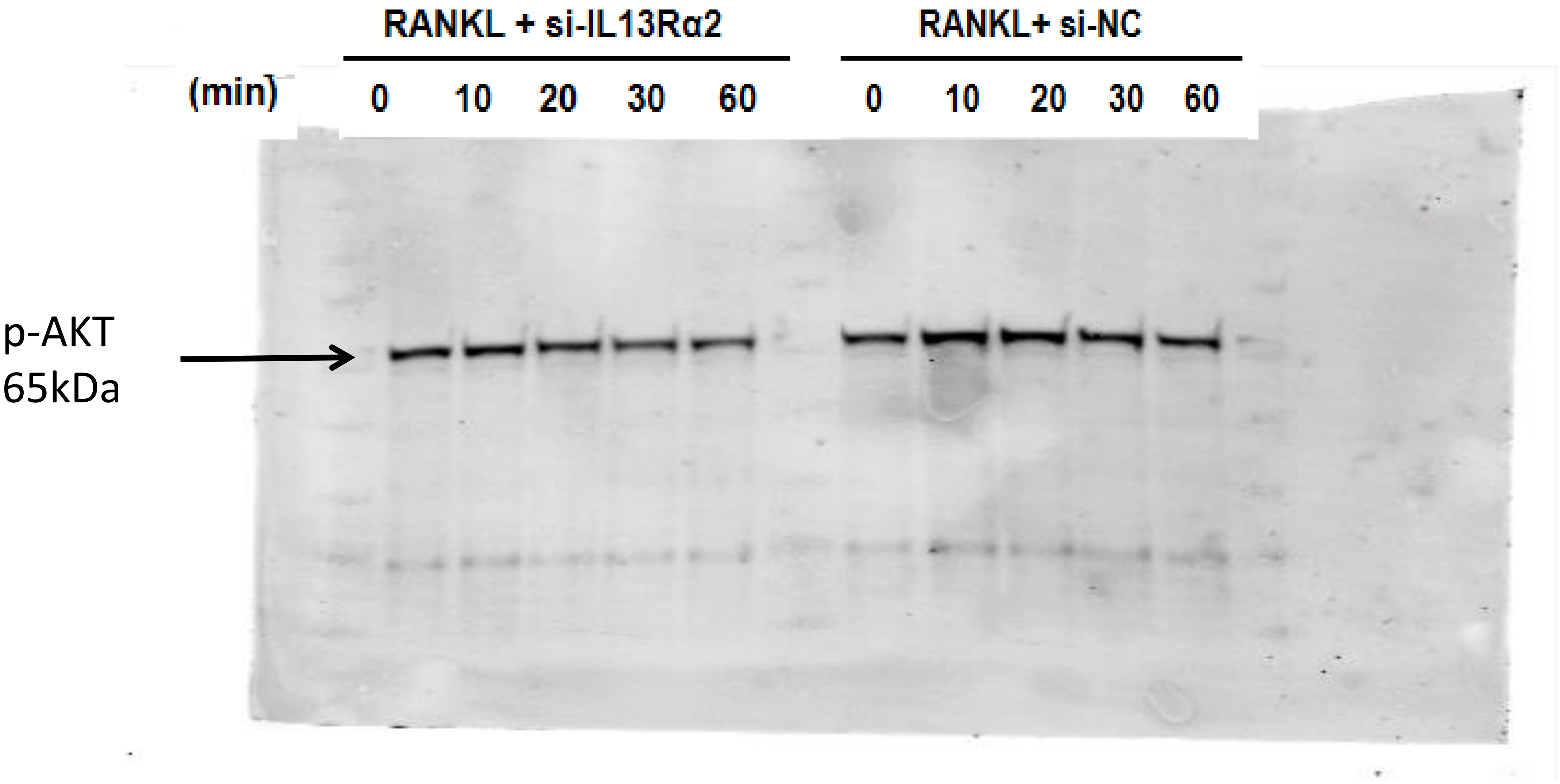
RANKL + si-IL13Rα2
RANKL+ si-NC
(min)
0 10 20 30 60
0 10 20 30 60
p-AKT
65kDa

Figure5A

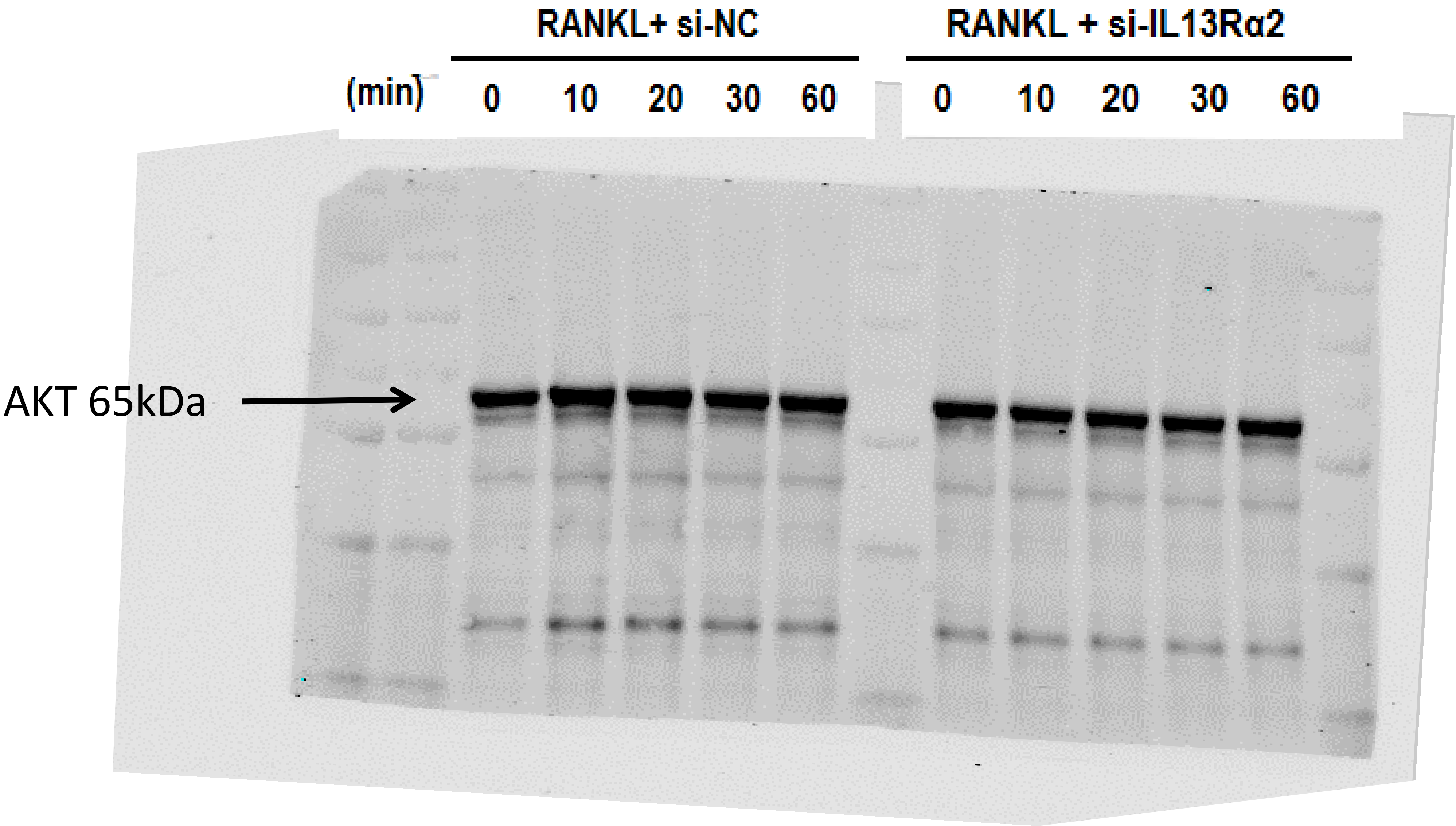
RANKL+ si-NC
RANKL + si-IL13Rα2
(min)
0
10
20
30
60
0
10
20
30
60
AKT 65kDa

Figure5A

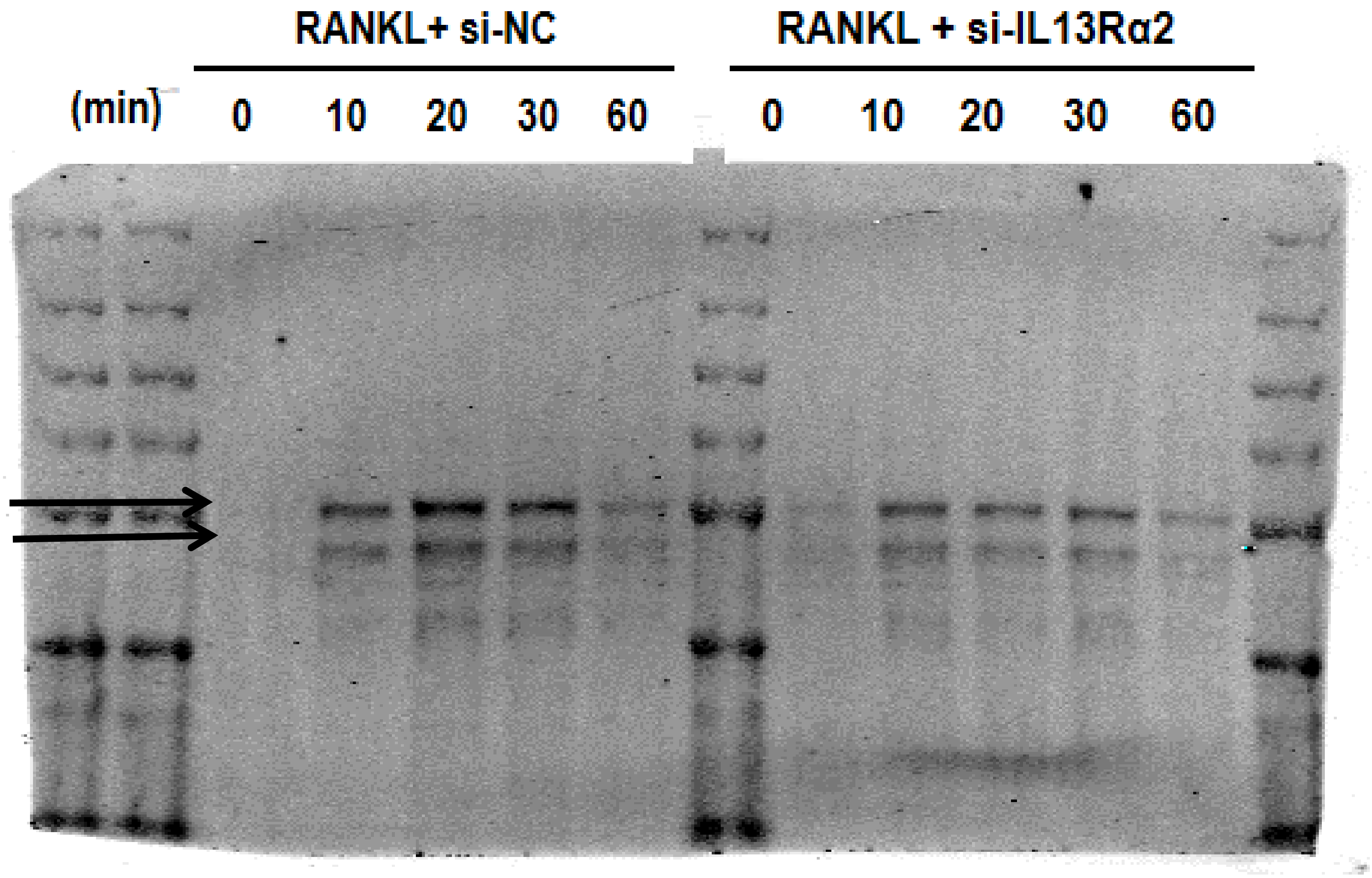
RANKL+ si-NC
RANKL + si-IL13Rα2
(min)
0
10
20
30
60
0
10
20
30
60
p-JNK
46 54kDa

Figure5A

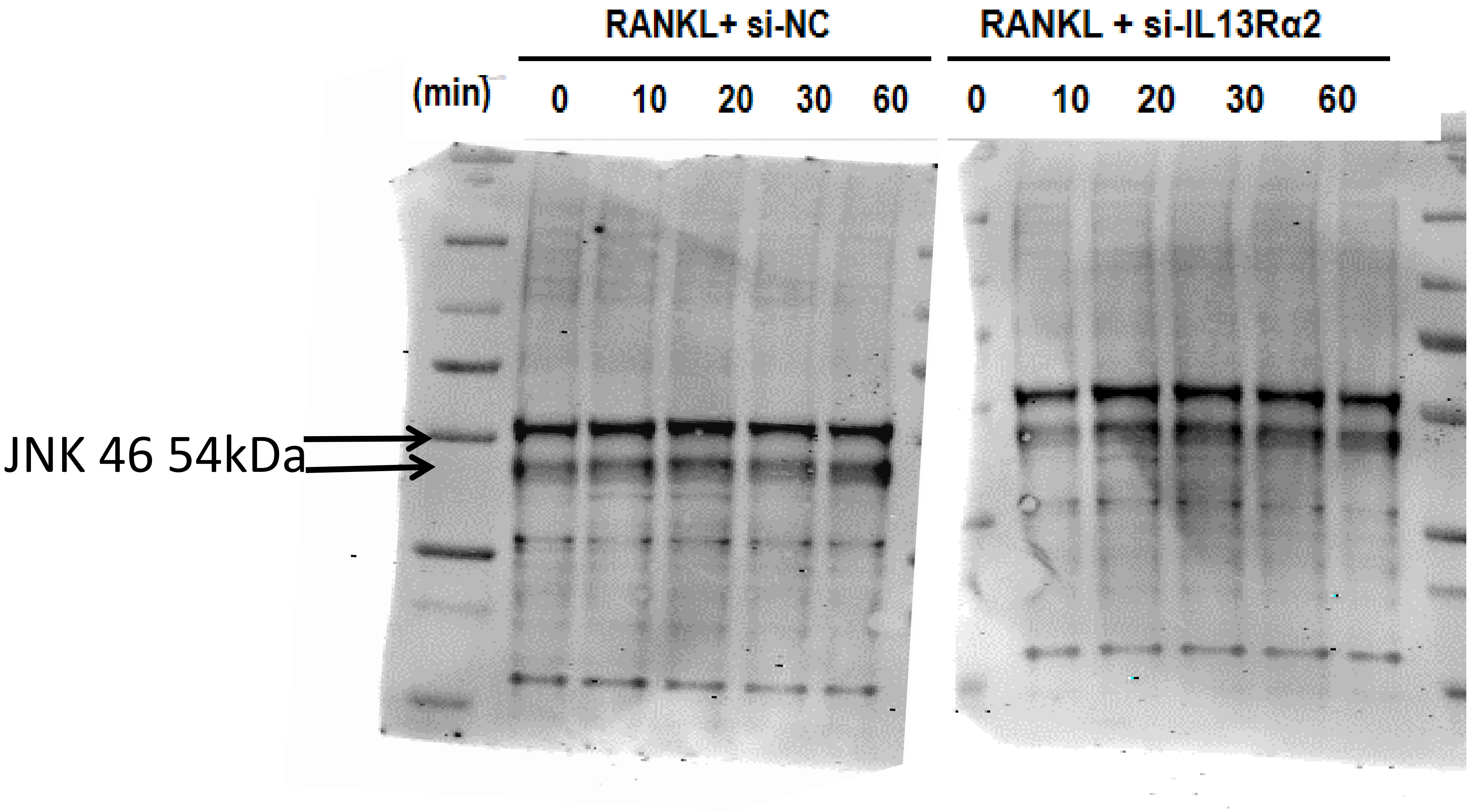
RANKL+ si-NC
RANKL + si-IL13Rα2
(min)
0
10
20
30
60
0
10
20
30
60
JNK 46 54kDa

Figure5A

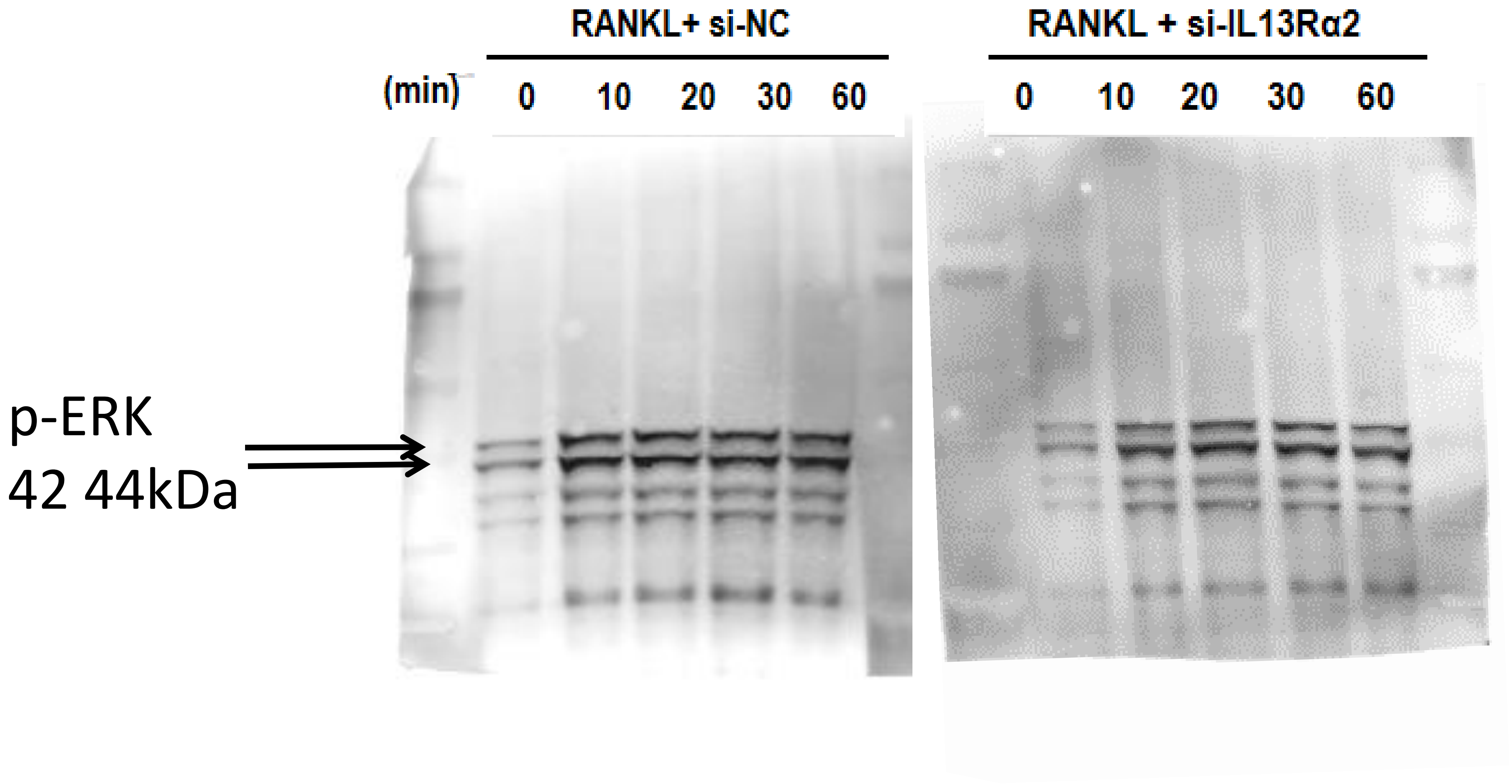
RANKL+ si-NC
RANKL + si-IL13Rα2
(min)
0
10
20
30
60
0
10
20
30
60
p-ERK
42 44kDa

Figure5A

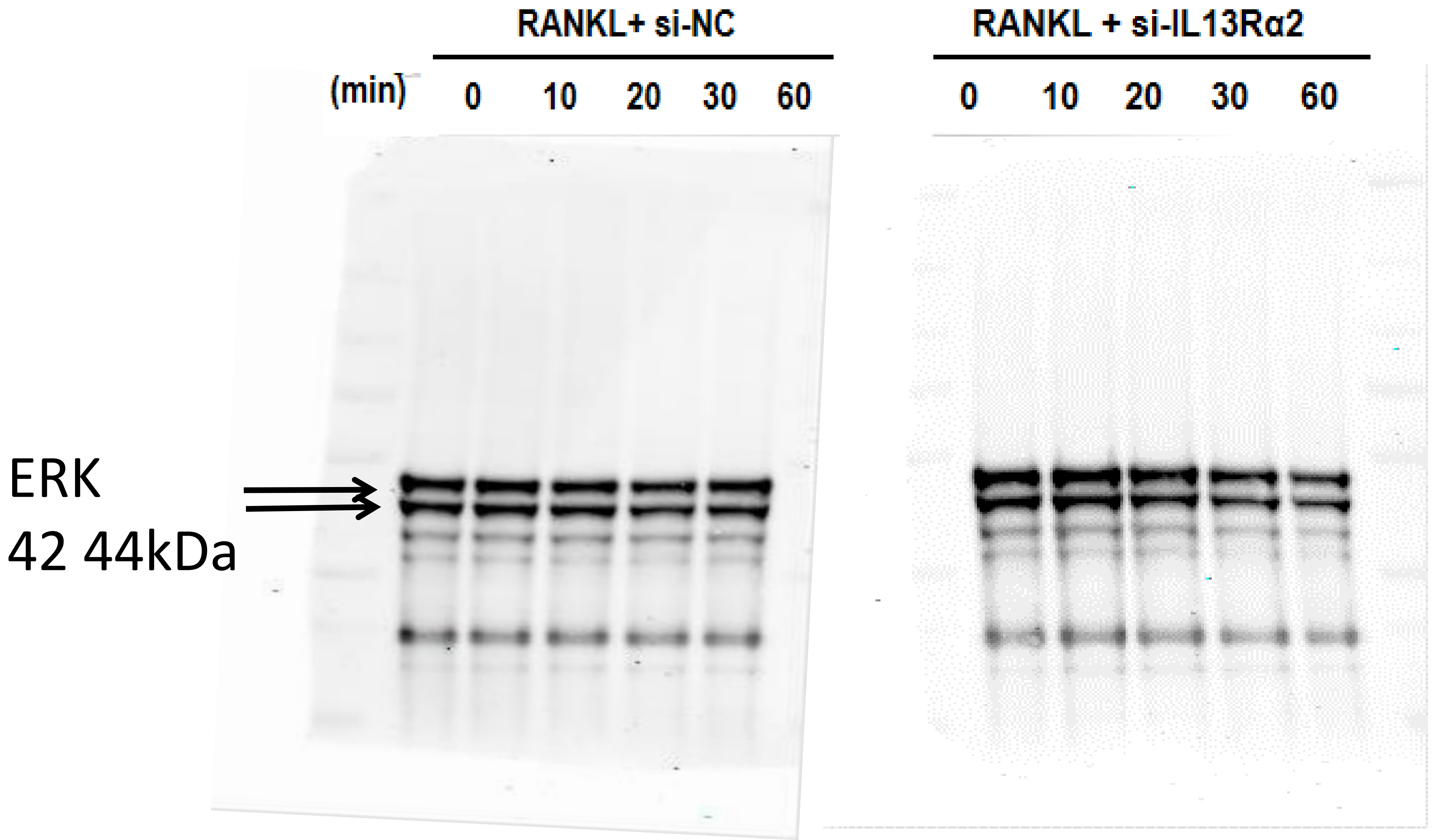
RANKL+ si-NC
RANKL + si-IL13Rα2
(min)
0 10 20 30 60
0 10 20 30 60
ERK
42 44kDa

Figure5A

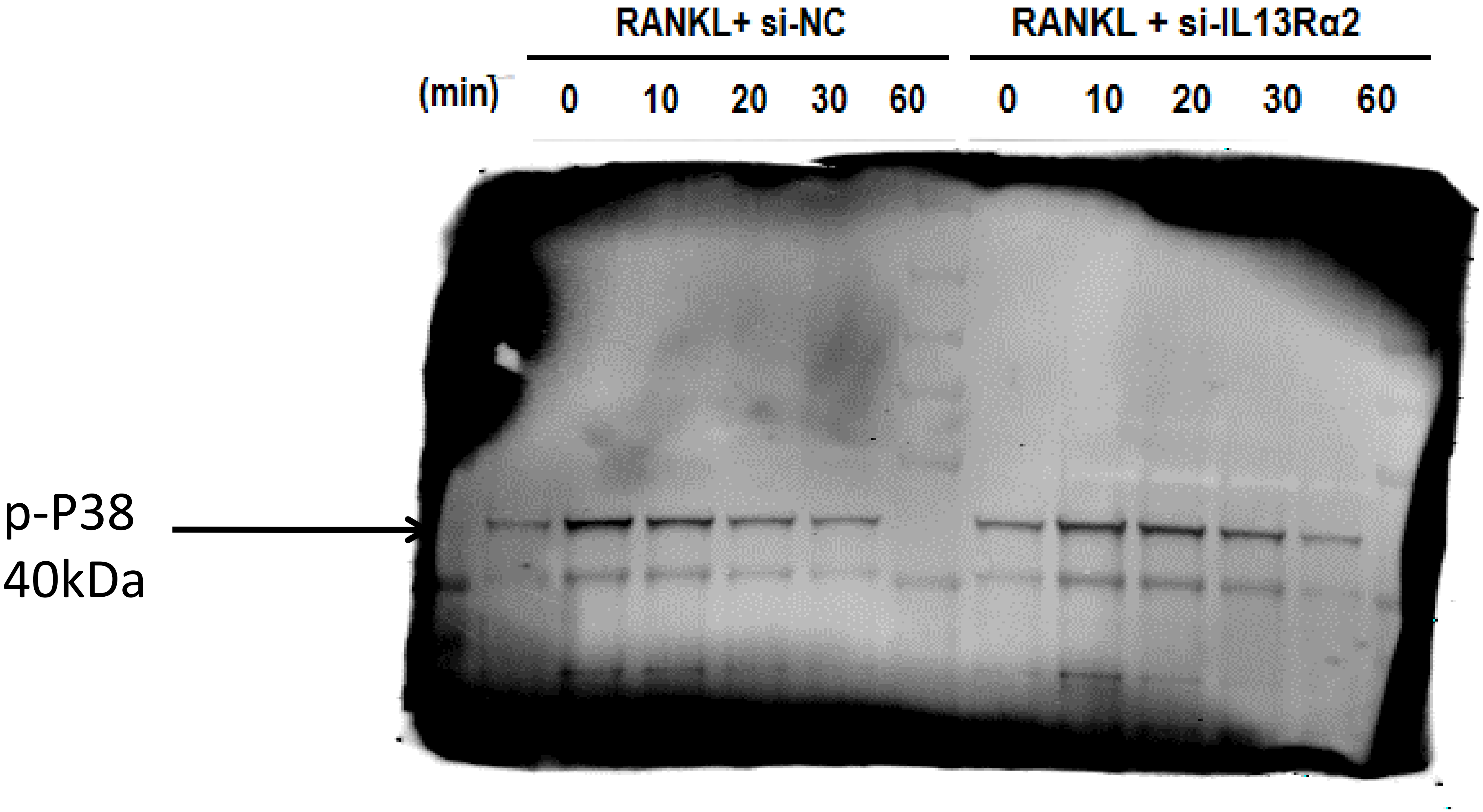

RANKL+ si-NC
RANKL + si-IL13Rα2
(min)
0 10 20 30 60
0 10 20 30 60
p-P38
40kDa

Figure5A

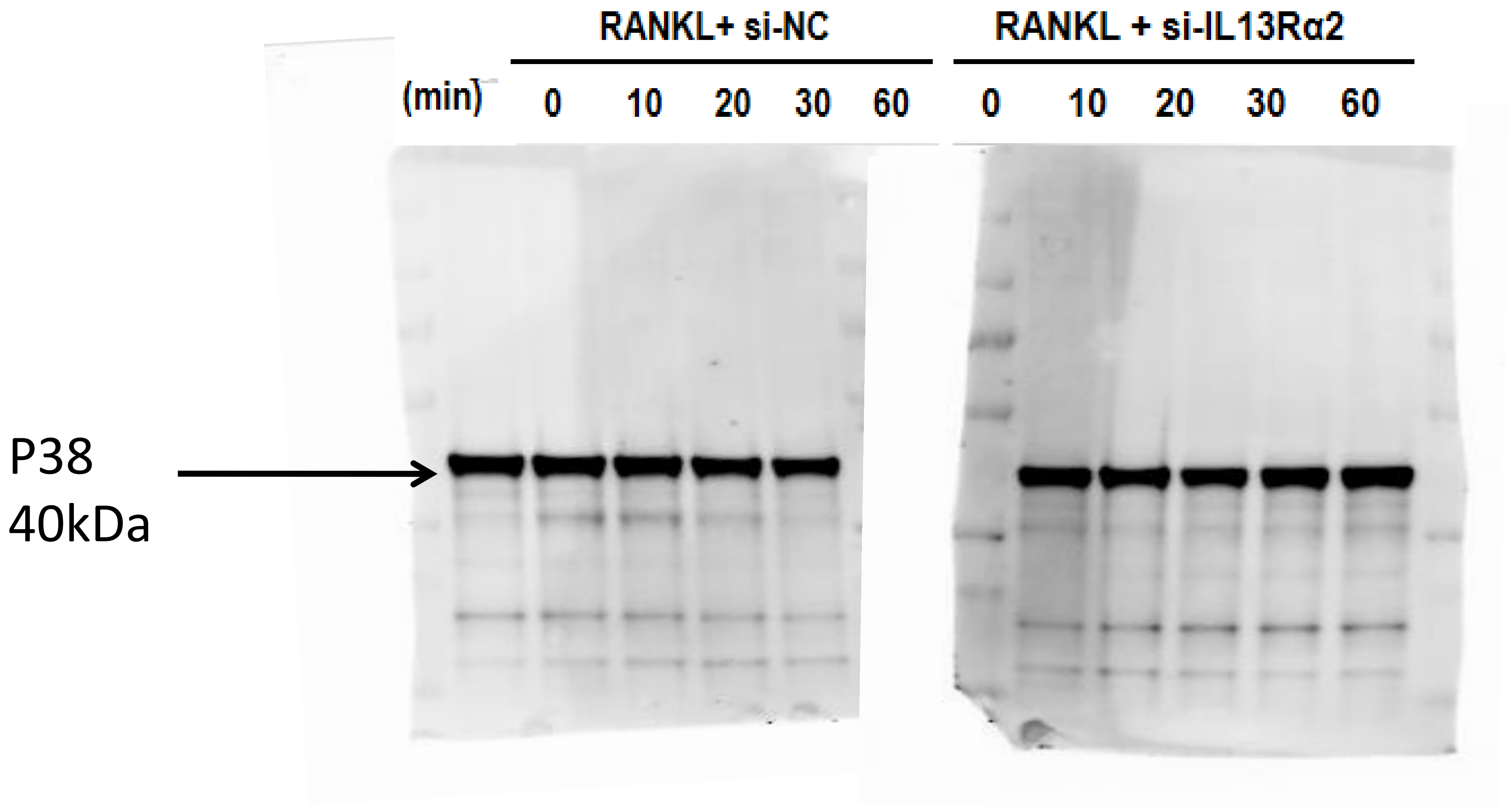
RANKL+ si-NC
RANKL + si-IL13Rα2
(min)
0 10 20 30 60
0 10 20 30 60
P38
40kDa

Figure5A

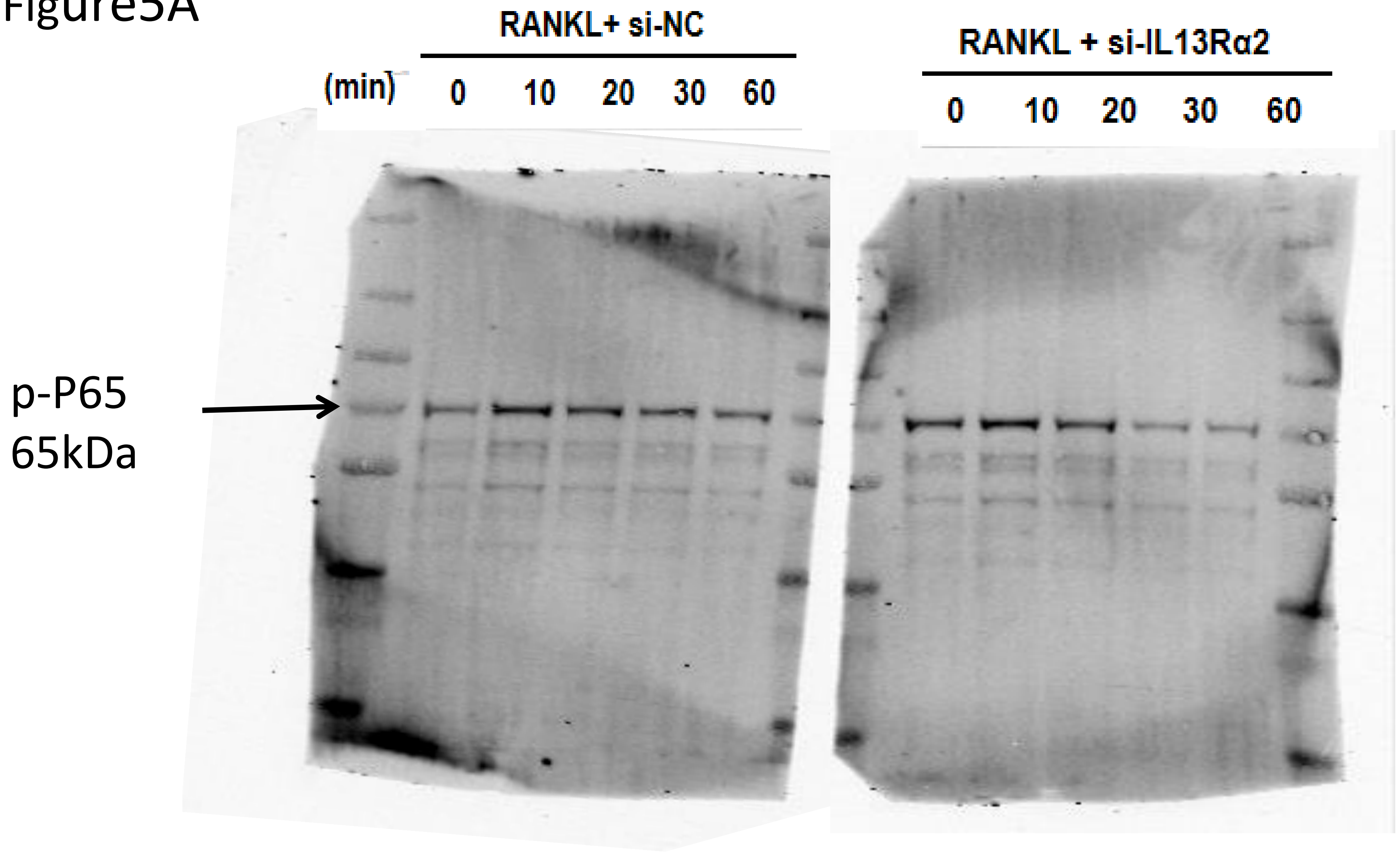
RANKL+ si-NC
RANKL + si-IL13Rα2
(min)
0 10 20 30 60
0 10 20 30 60
p-P65
65kDa

Figure5A

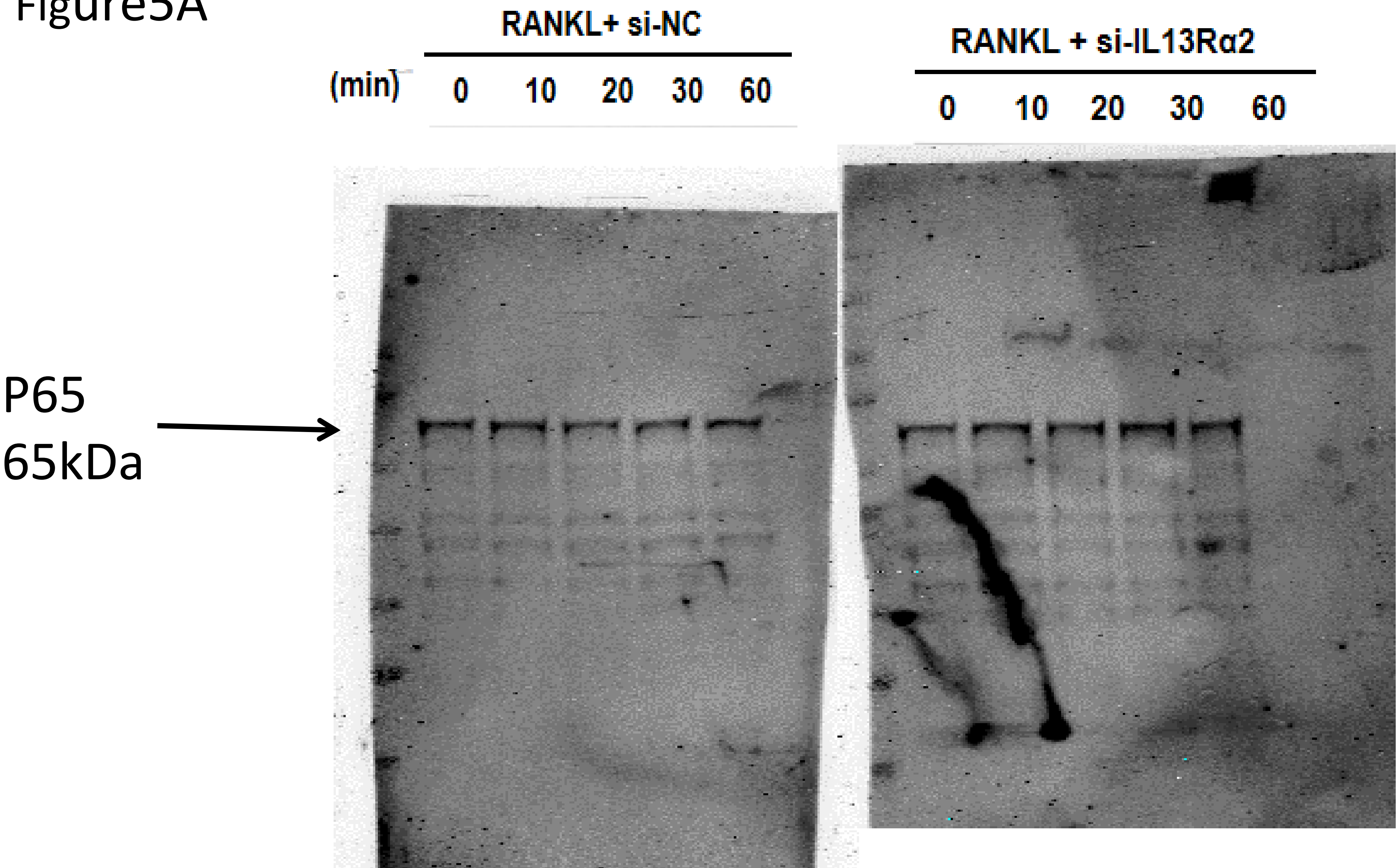

RANKL+ si-NC
(min)
0
10
20
30
60
RANKL + si-IL13Rα2
0
10
20
30
60
P65
65kDa

Figure5A

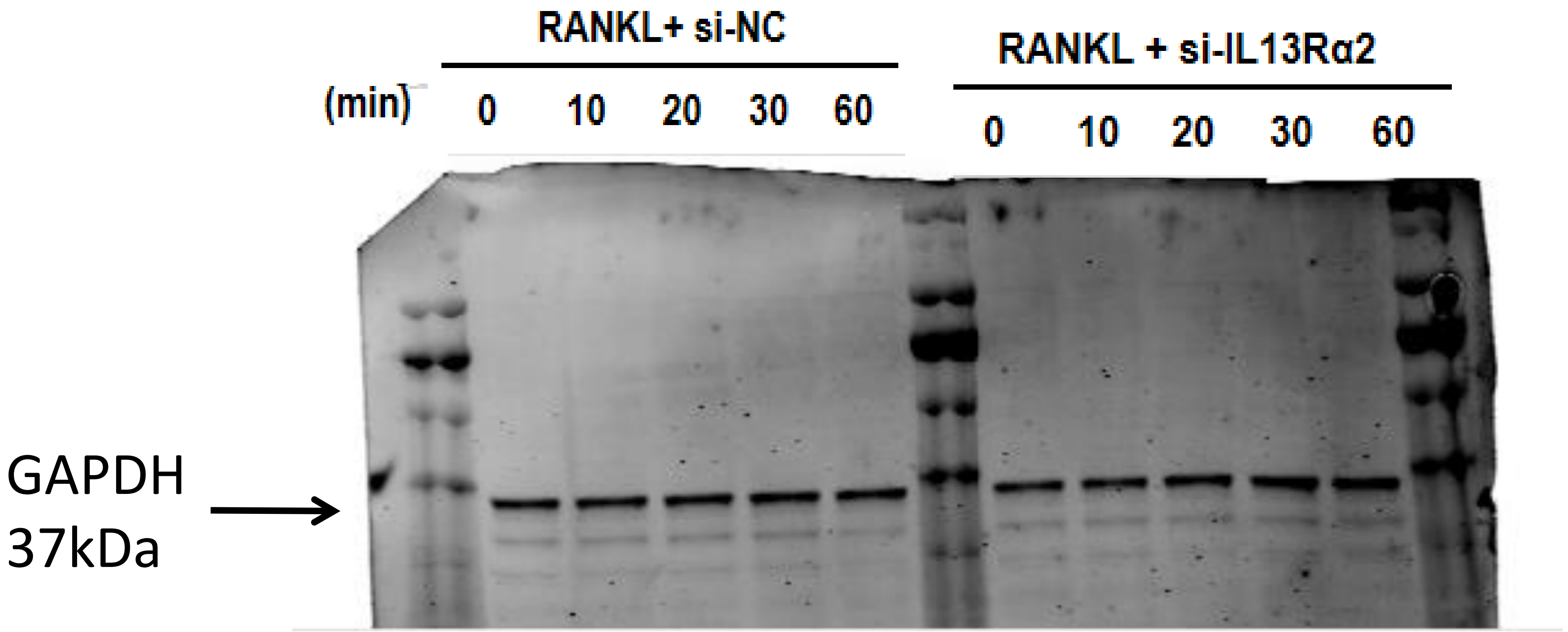

RANKL+ si-NC
RANKL + si-IL13Rα2
(min)
0
10
20
30
60
0
10
20
30
60
GAPDH
37kDa

figure 6 C

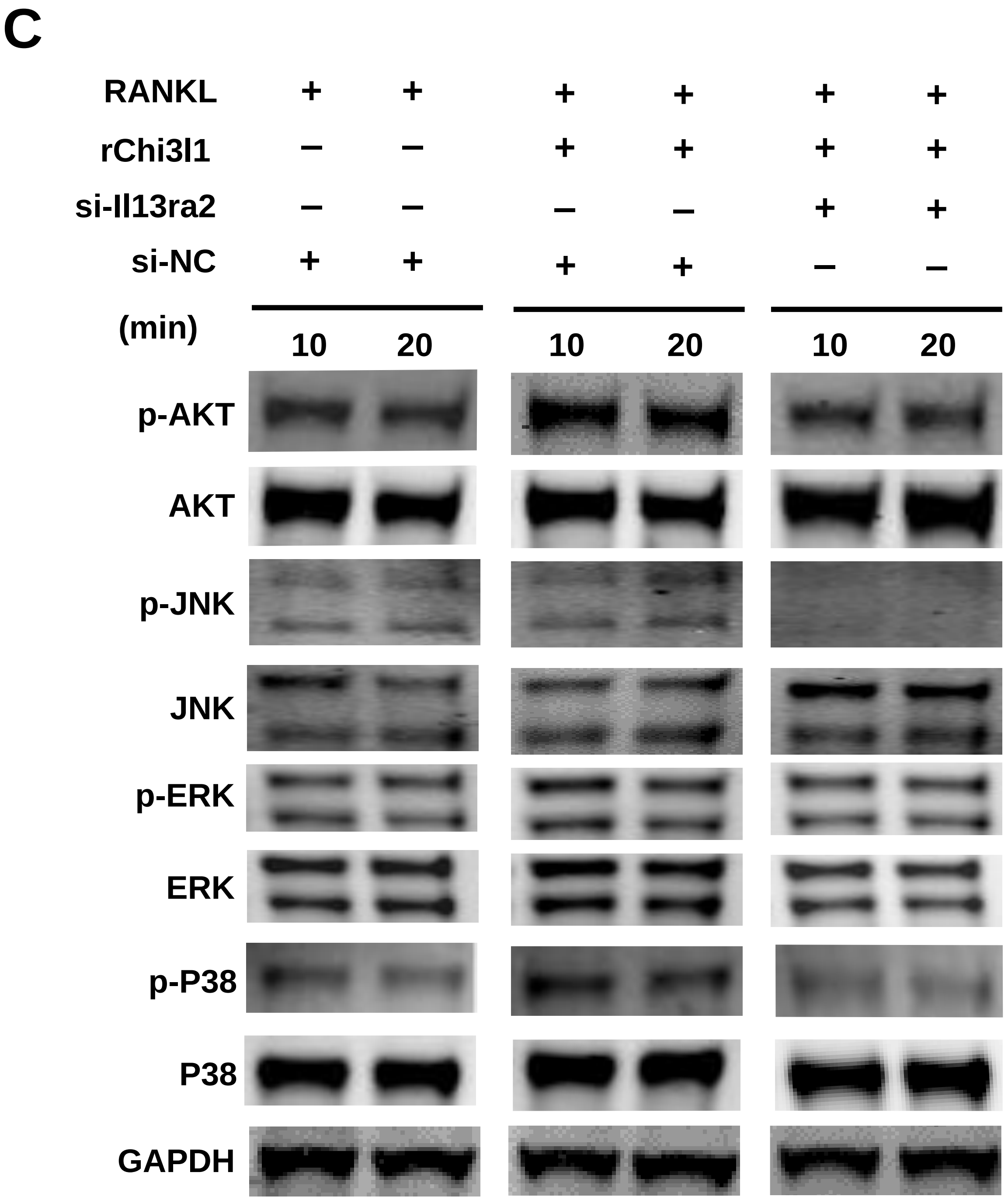
C
RANKL + + + + + +
rChi3l1 − − + + + +
si-Il13ra2 − − − − + +
si-NC + + + + − −
(min)
10 20 10 20 10 20
p-AKT
AKT
p-JNK
JNK
p-ERK
ERK
p-P38
P38
GAPDH

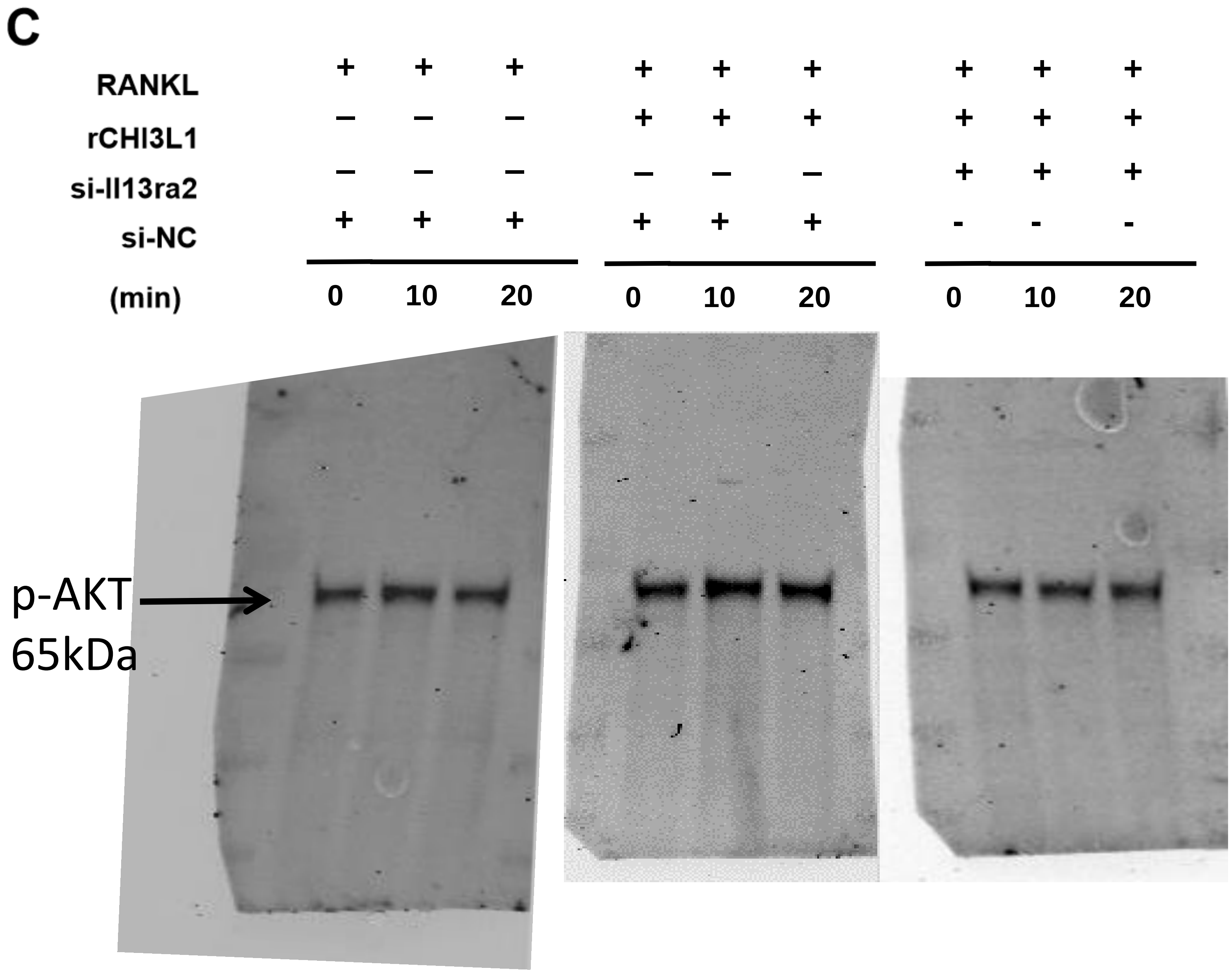
C
RANKL
rCHI3L1
si-Il13ra2
si-NC
(min)
0
10
20
p-AKT
65kDa

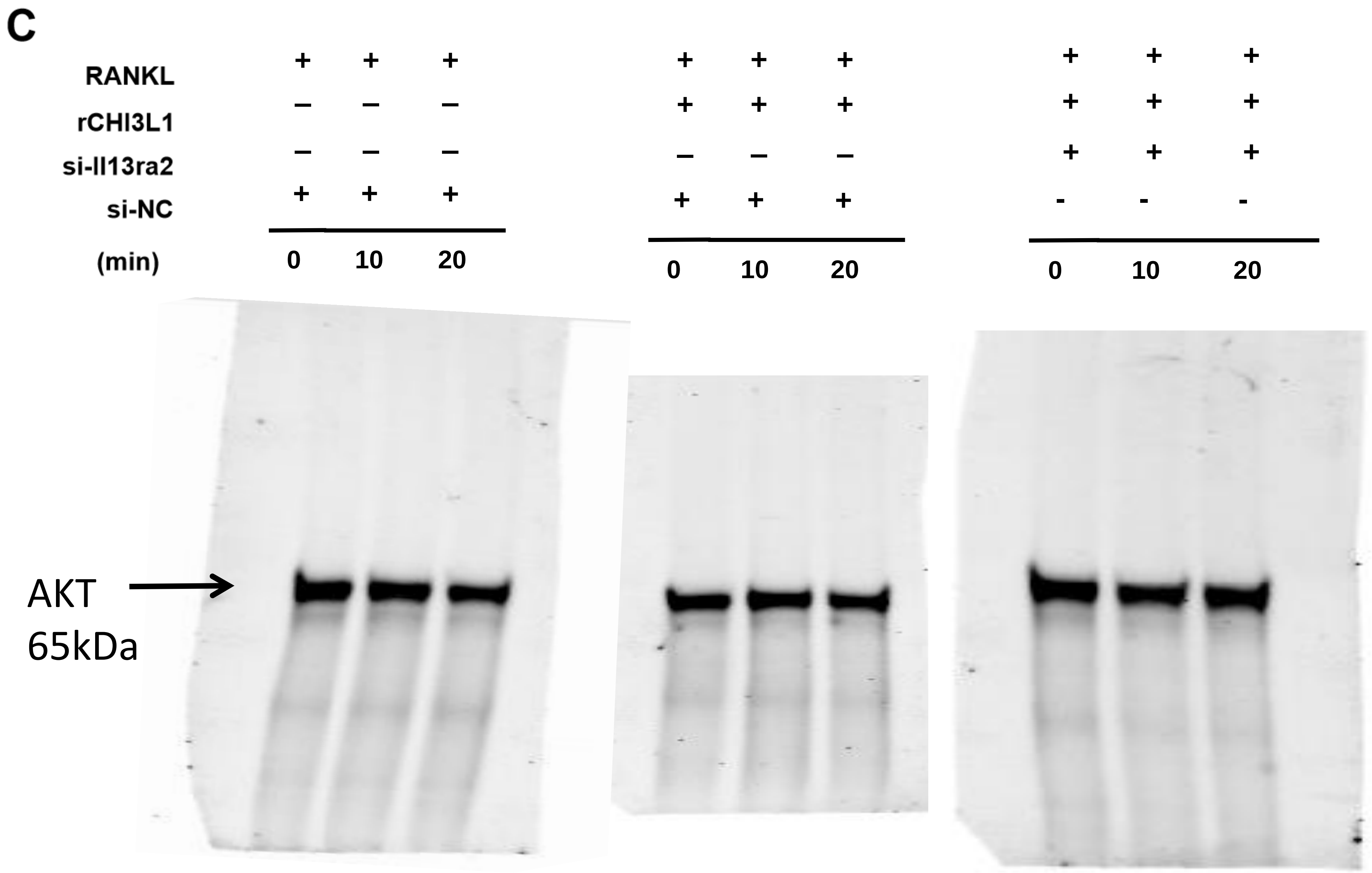
C
RANKL
rCHI3L1
si-Il13ra2
si-NC
(min)
+ + +
– – –
– – –
+ + +
0 10 20
+ + +
+ + +
– – –
+ + +
0 10 20
+ + +
+ + +
+ + +
- - -
0 10 20
AKT
65kDa

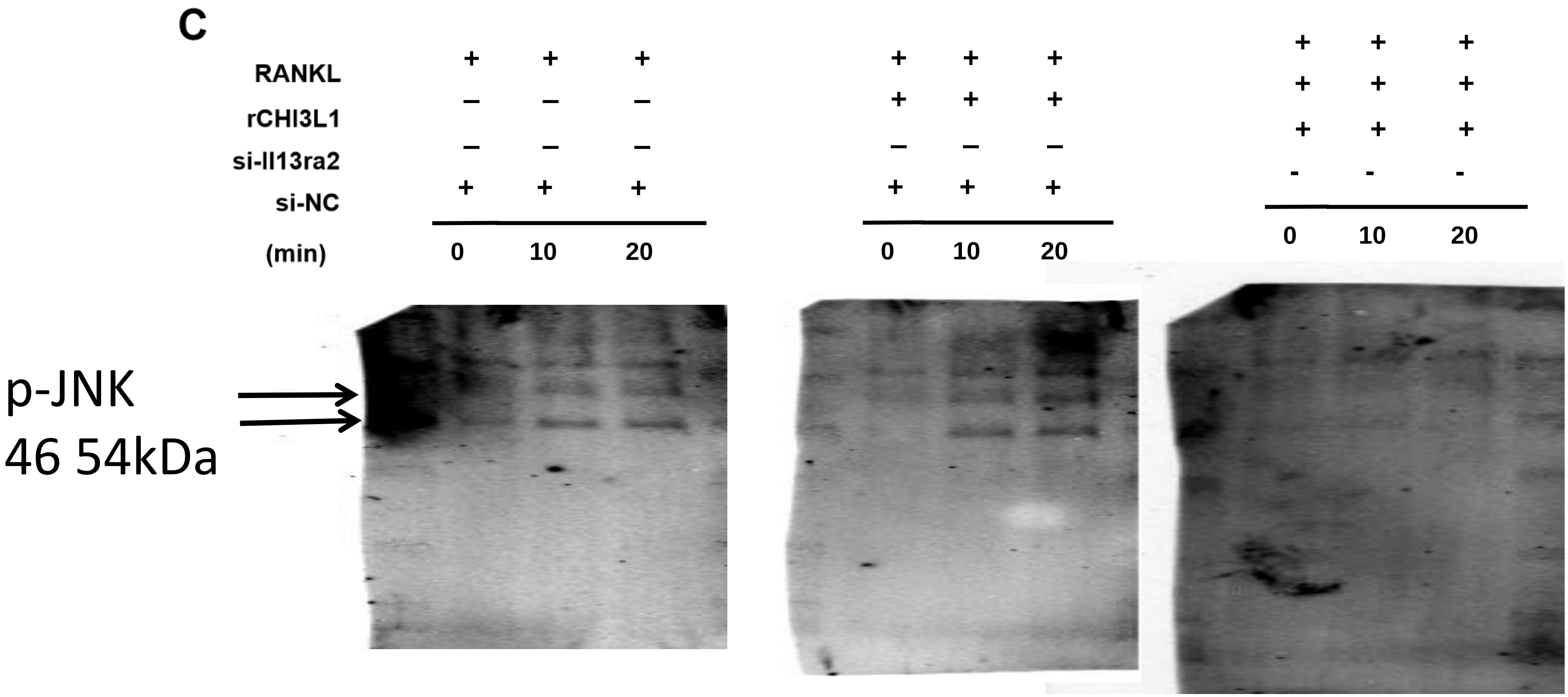
C
RANKL
rCHI3L1
si-Il13ra2
si-NC
(min)
0 10 20
0 10 20
0 10 20
p-JNK
46 54kDa

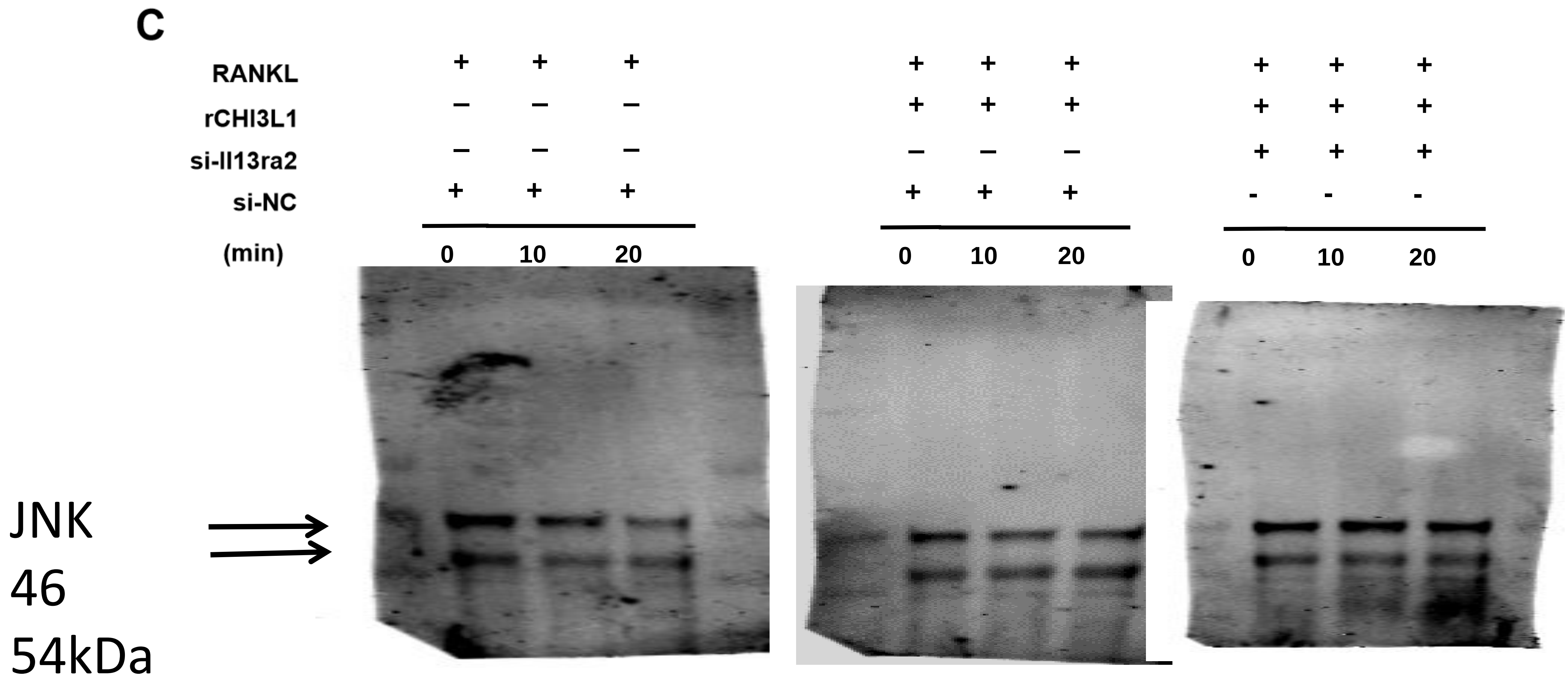
C
RANKL
rCHI3L1
si-Il13ra2
si-NC
(min)
+ + +
− − −
− − −
+ + +
0 10 20
+ + +
+ + +
− − −
+ + +
0 10 20
+ + +
+ + +
+ + +
- - -
0 10 20
JNK
46
54kDa

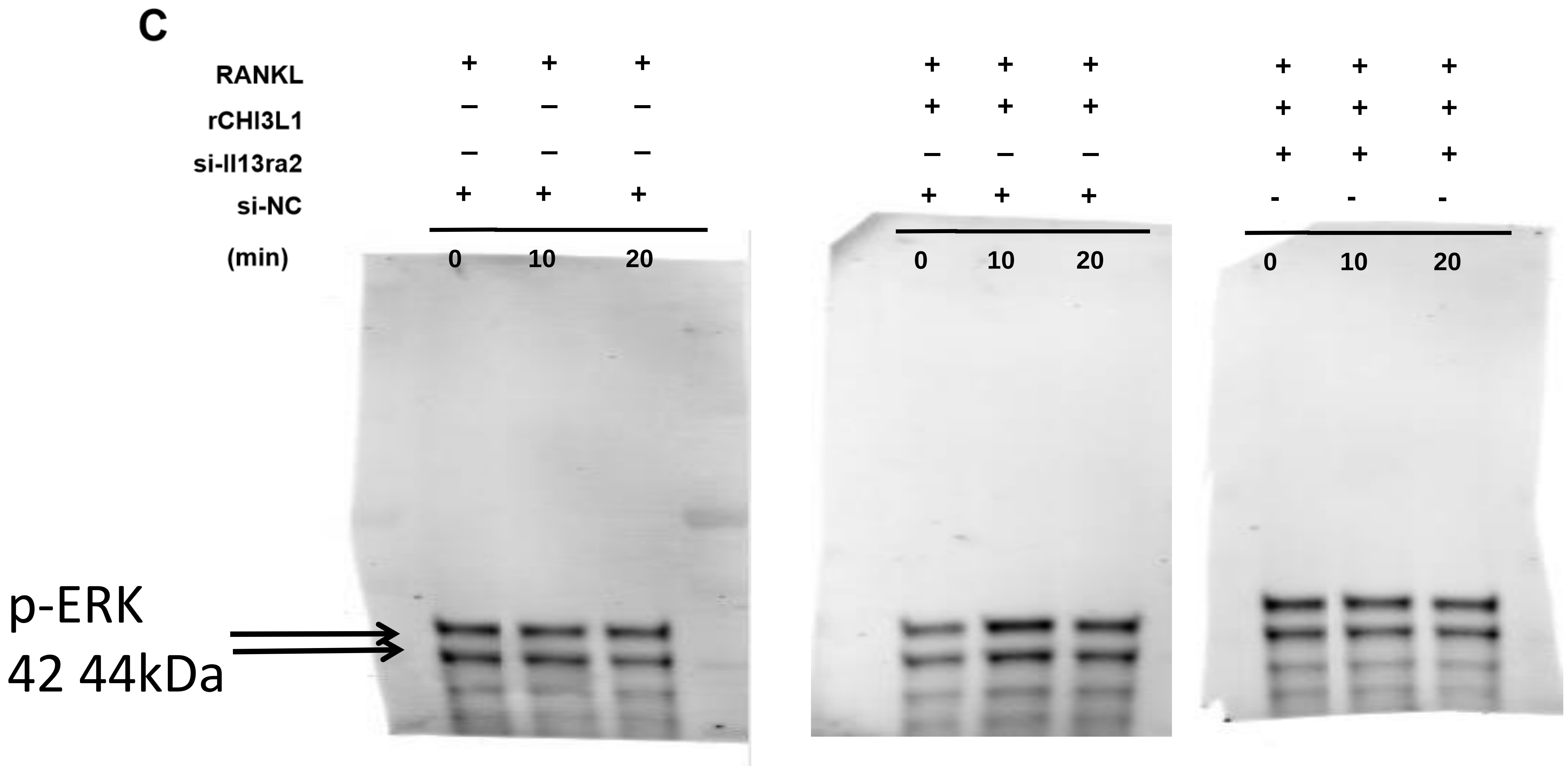
C
RANKL
rCHI3L1
si-Il13ra2
si-NC
(min)
+ + +
– – –
– – –
+ + +
0 10 20
+ + +
+ + +
– – –
+ + +
0 10 20
+ + +
+ + +
+ + +
- - -
0 10 20
p-ERK
42 44kDa

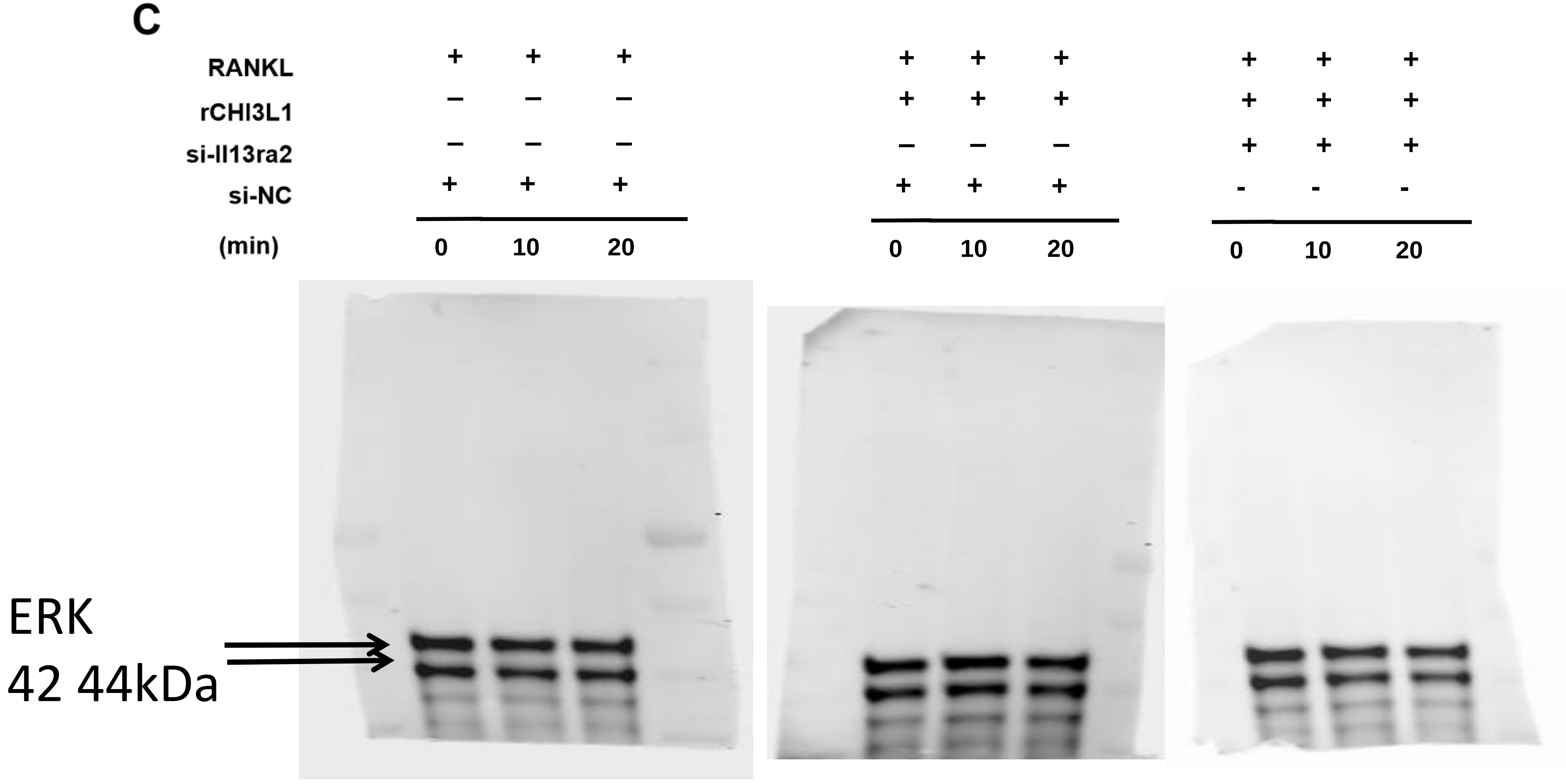
C
RANKL
rCHI3L1
si-Il13ra2
si-NC
(min)
0 10 20
0 10 20
0 10 20
ERK
42 44kDa

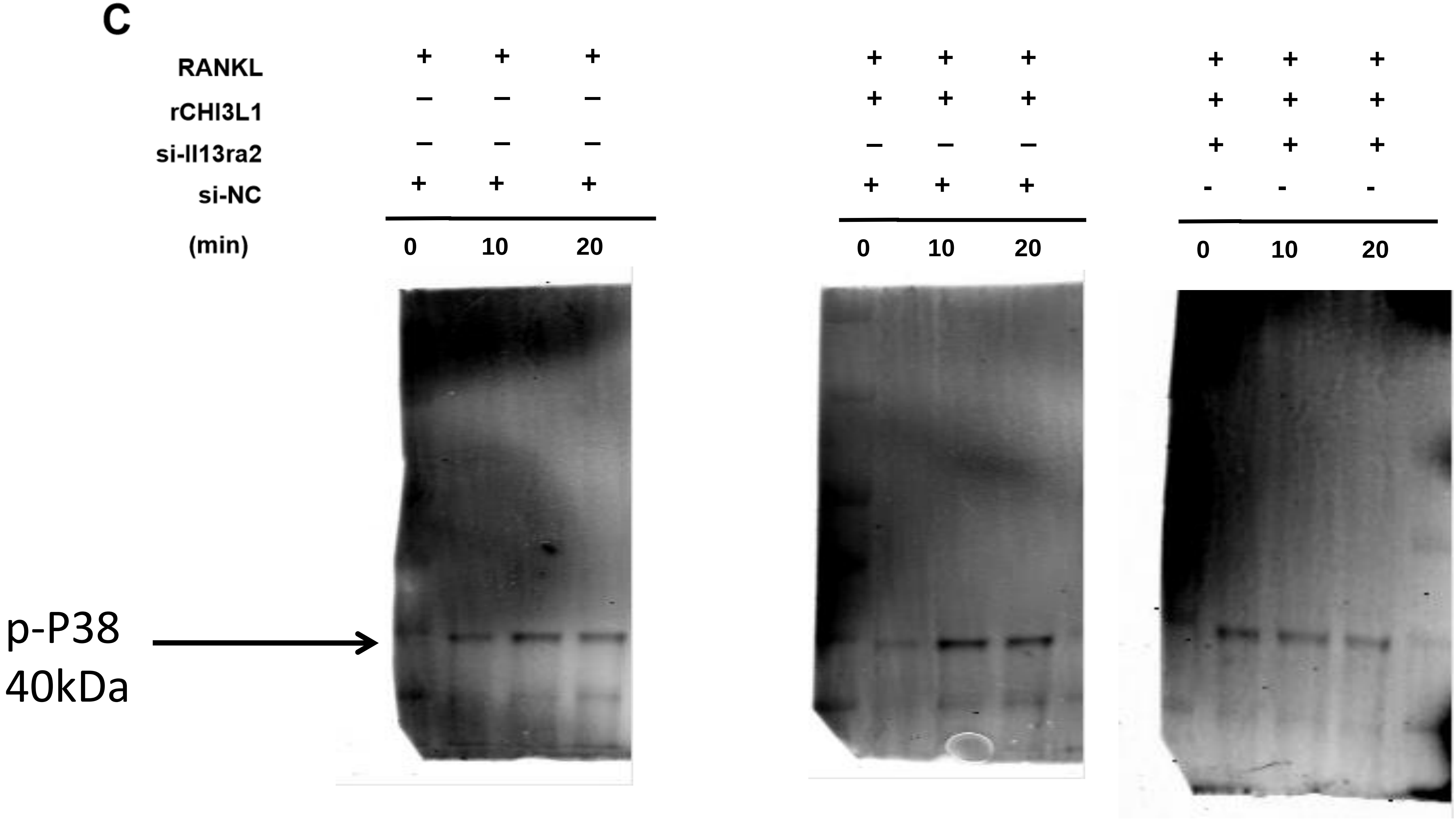

C
RANKL + + + + + + + + +
rCHI3L1 – – – + + + + + +
si-Il13ra2 – – – – – – + + +
si-NC + + + + + + - - -
(min) 0 10 20 0 10 20 0 10 20
p-P38
40kDa

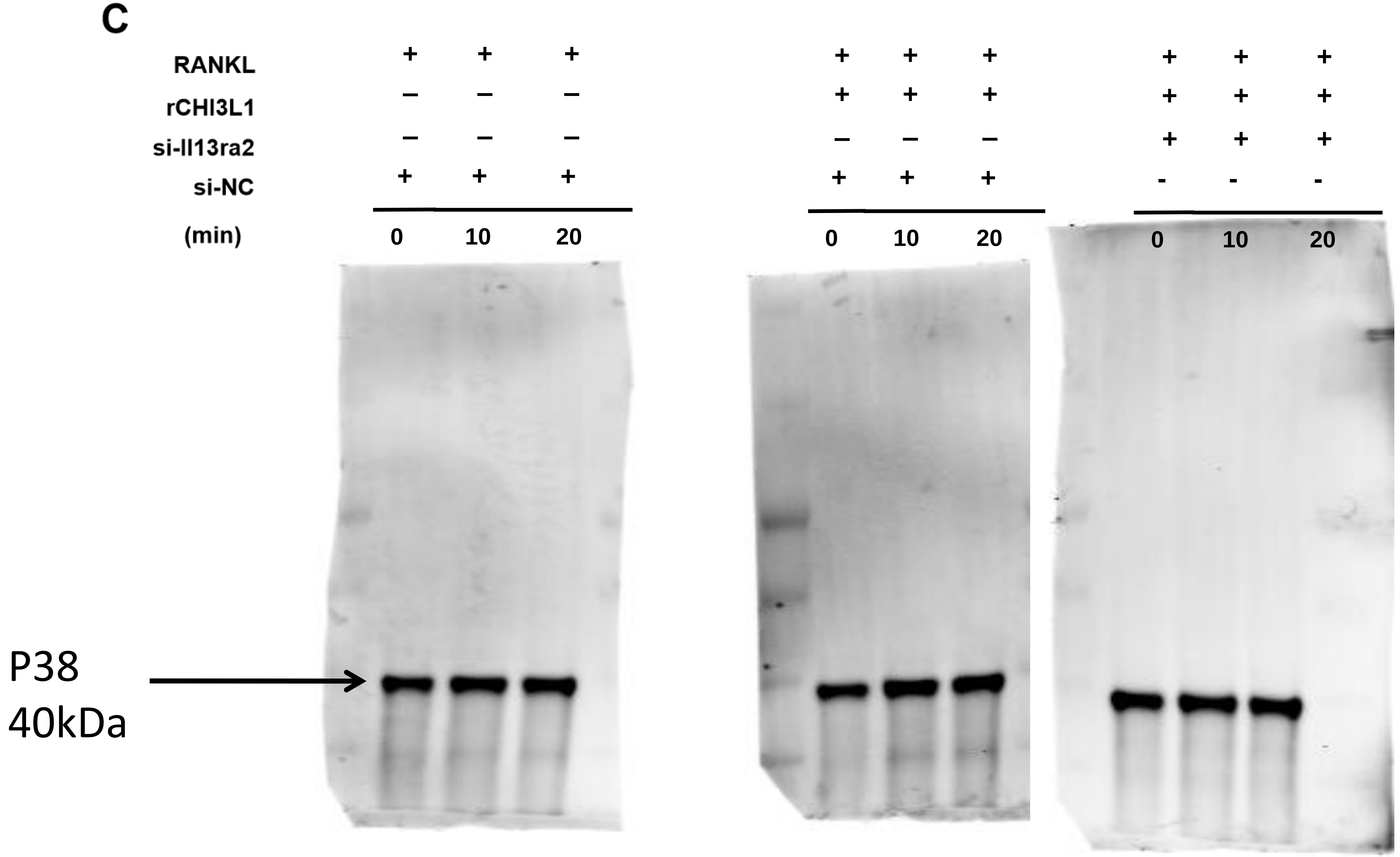
C
RANKL
rCHI3L1
si-Il13ra2
si-NC
(min)
0
10
20
0
10
20
0
10
20
P38
40kDa

C

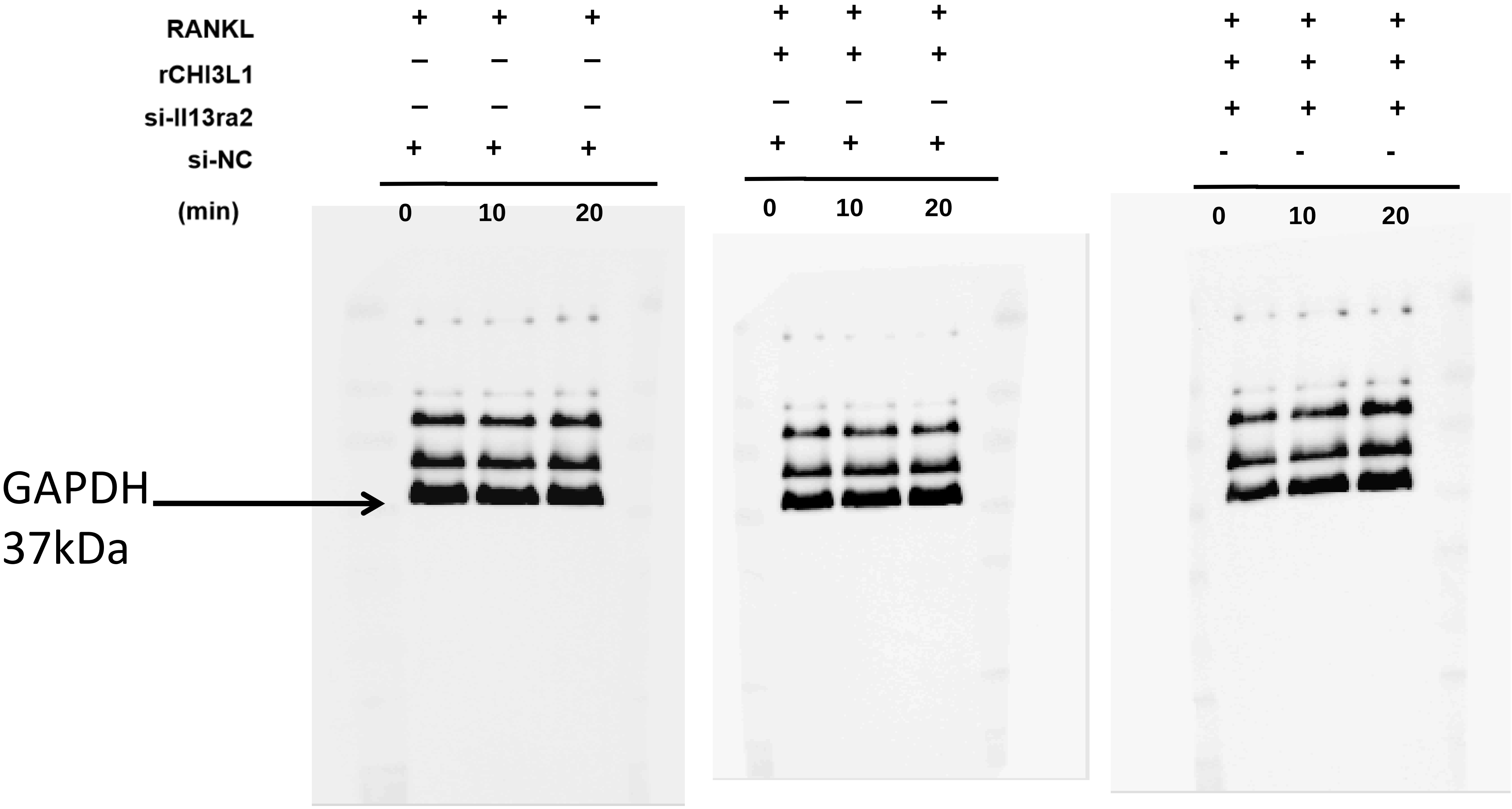

Supplement: Supplementary file 2 — Additional file 1: Supplemental Figure 1. Neither recombinant Chi3l1/sIL13Rα2protein nor Chi3l1/sIL13Rα2 silencing had any effect on the proliferative ability of BMMs. A BMMs were treated with M-CSF and various concentrations of chi31l recombinant protein (0, 125, 250, 500, 1000ng/ml) for 48h and 96h, then the proliferative ability of BMMs was determined by the CCK-8 experiment. B After silencing Chi3l1or IL13Rα2 expression in BMMs with siRNA for 48h, BMMs were treated with M-CSF for 48h and 96h, then the proliferative ability of BMMs was determined by the CCK-8 experiment. C BMMs were treated with M-CSF and various concentrations of rIL13Rα2 protein (0, 125, 250, 500, 1000ng/ml) for 48h and 96h, then the proliferative ability of BMMs was determined by the CCK-8 experiment. **P < 0.01. Results are expressed as means ± SE. [file 12964_2023_1423_MOESM1_ESM.zip › Supplementary/western blot.pdf]
