# Supplementary figures and images for: IL13Rα2 as a crucial receptor for Chi3l1 in osteoclast differentiation and bone resorption through the MAPK/AKT pathway
Source: Cell Commun Signal. 2024 Jan 30;22:81. doi: 10.1186/s12964-023-01423-7 (PMC10826115; doi:10.1186/s12964-023-01423-7)

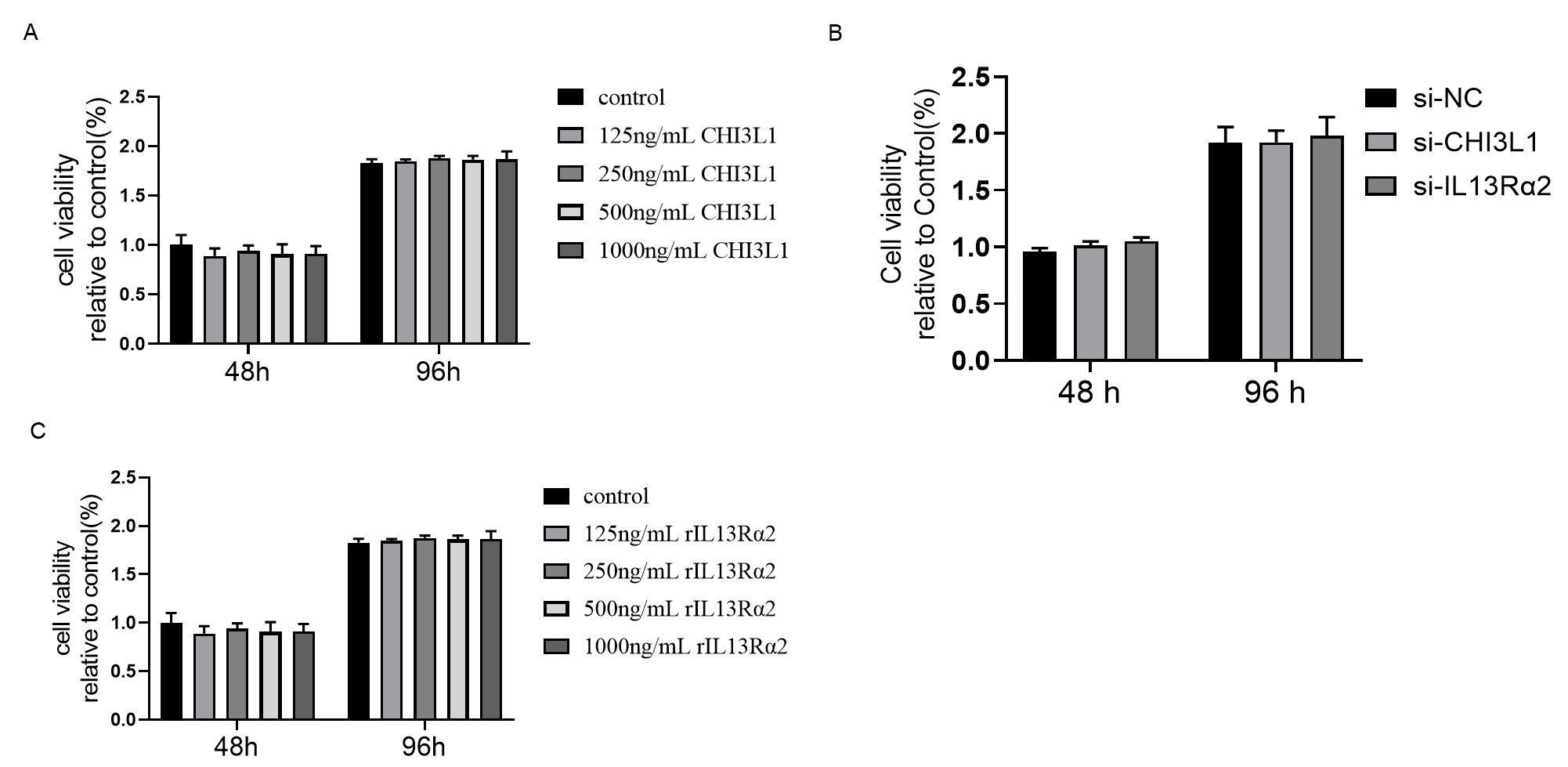

Supplement: Supplementary file 2 — Additional file 1: Supplemental Figure 1. Neither recombinant Chi3l1/sIL13Rα2protein nor Chi3l1/sIL13Rα2 silencing had any effect on the proliferative ability of BMMs. A BMMs were treated with M-CSF and various concentrations of chi31l recombinant protein (0, 125, 250, 500, 1000ng/ml) for 48h and 96h, then the proliferative ability of BMMs was determined by the CCK-8 experiment. B After silencing Chi3l1or IL13Rα2 expression in BMMs with siRNA for 48h, BMMs were treated with M-CSF for 48h and 96h, then the proliferative ability of BMMs was determined by the CCK-8 experiment. C BMMs were treated with M-CSF and various concentrations of rIL13Rα2 protein (0, 125, 250, 500, 1000ng/ml) for 48h and 96h, then the proliferative ability of BMMs was determined by the CCK-8 experiment. **P < 0.01. Results are expressed as means ± SE. [file 12964_2023_1423_MOESM1_ESM.zip › Supplementary/Supplemental Figure 1.TIF]
